# HIV & Hepatitis in the Americas 28–30 April 2016, Mexico City, Mexico

**DOI:** 10.7448/IAS.19.2.21083

**Published:** 2016-04-28

**Figure 1:**
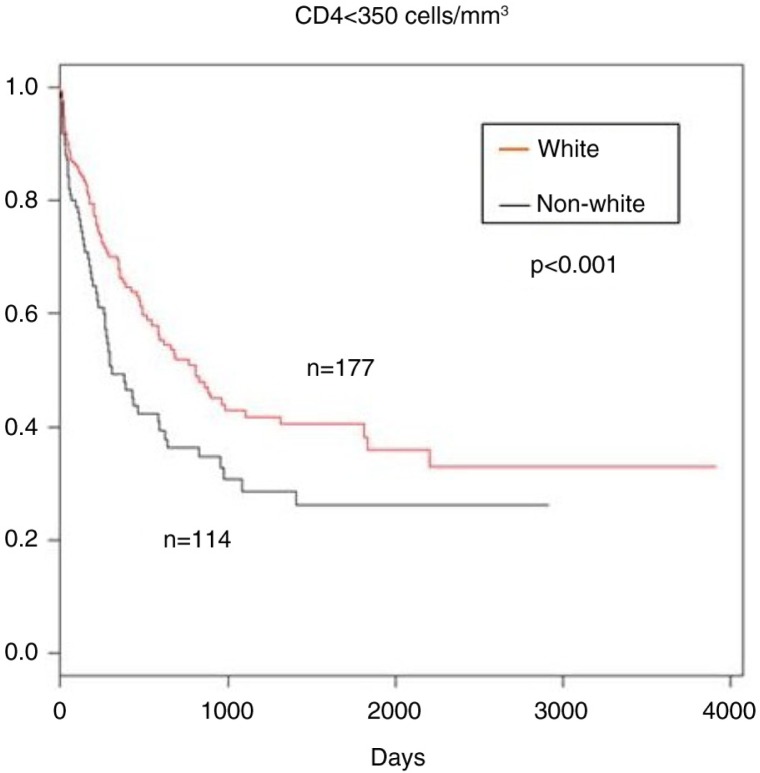
Kaplan–Meier survival curves: time in days from estimated date of seroconversion to CD4<350 cells/mm^3^, according to race (white vs. non-white).
