# Supplementary material for: HIV & Hepatitis in the Americas 28–30 April 2016, Mexico City, Mexico
Source: J Int AIDS Soc. 2016 Apr 28;19(2Suppl 1):21083. doi: 10.7448/IAS.19.2.21083 (PMC4851728; doi:10.7448/IAS.19.2.21083)
Supplement: Related texts

# Sub article: What about the new drugs for hepatitis?

**DOI:** 10.7448/IAS.19.2.21010

**Published:** 2016-04-28

**Authors:** Jordan Feld

**Affiliations:** Toronto Western Hospital Liver Center, Toronto, ON, Canada

## Abstract

The development of potent interferon-free regimens of direct-acting antivirals (DAAs) for the treatment of hepatitis C virus (HCV) infection has moved at a remarkable pace. Highly effective, extremely well-tolerated regimens are now available for most patient populations. Remarkably, advances have moved as quickly for those with HIV–HCV co-infection as for those with HCV monoinfection, with recent studies showing similar results with most regimens in both populations. This has raised the question of whether HIV co-infection continues to represent a “special” population among HCV-infected individuals. There are, however, some data suggesting that HIV may still impair responses in particularly difficult-to-cure populations. For example, attempts to shorten therapy to eight weeks with sofosbuvir/NS5A combinations have been at least somewhat less successful in HIV-co-infected patients than in those with HCV monoinfection. It is clear that innate and possibly adaptive immune function are still necessary for viral clearance even with highly effective DAA-based regimens, and thus, it is possible that particularly as therapy is pushed to shorter and simpler combinations, HIV may again emerge as an issue necessitating special consideration. The rationale behind DAA combinations will be reviewed with a focus on relevance for HIV-HCV co-infection. A general discussion of remaining issues to be addressed including drug interactions and treatment access will also be included.

# Sub article: Injection drug use and HIV/HCV epidemics

**DOI:** 10.7448/IAS.19.2.21009

**Published:** 2016-04-28

**Authors:** Nora D Volkow

**Affiliations:** National Institute on Drug Abuse (NIDA), Bethesda, MA, USA

## Abstract

Injection drug use (IDU) is an important vector in HIV and hepatitis C virus (HCV) transmission. Though the United States, Canada and western Europe have had major successes in controlling the HIV epidemic due to IDU, that is not the case in eastern Europe and Southeast Asia. Moreover, the recent increases of IDU in the United States fuelled by the epidemic of prescription opioid abuse were responsible for last year's HIV outbreak in Indiana and for the increases in HCV across the country and highlight the continued importance of IDU in fuelling these two epidemics. Injecting drug users infected with HIV or HCV can quickly lead to generalized HIV and HCV epidemics as they serve as bridges to other populations through sexual transmission. This highlights the importance of addiction treatment of IDU (particularly medication-assisted treatment or MAT) as prevention strategy for HIV and HCV infection as well as “antiretroviral therapy treatment as prevention strategy” to prevent HIV transmission. Addiction treatment also improves adherence to antiretroviral and clinical outcomes for HIV. The role of MAT in the prevention and outcomes for HCV has been much less studied. However, despite the effectiveness of MAT for the treatment of opioid use disorders, its use in IDU is very restricted. Strategies are required to expand its use within the healthcare system including infectious disease clinics as well as criminal justice settings. Success in its implementation will be necessary for achieving an AIDS-free generation and also for containing the growing HCV epidemic.

# Sub article: HIV cure research: update from CROI 2016

**DOI:** 10.7448/IAS.19.2.21011

**Published:** 2016-04-28

**Authors:** Daniel R Kuritzkes

**Affiliations:** Brigham and Women's Hospital, Harvard Medical School, Boston, MA, USA

## Abstract

The advent of potent combination antiretroviral therapy (ART) has led to a dramatic decrease in the incidence of AIDS and AIDS-related mortality worldwide. For most patients, full suppression of HIV type 1 (HIV-1) replication can be achieved by once-daily administration of an ART regimen available as a fixed-dose combination that is safe, convenient and well tolerated. Nevertheless, a treatment that led to durable drug-free remission or eradication (cure) of HIV-1 could reduce the burden, cost, toxicities and stigma associated with long-term ART and might lower immune activation and the associated risk of non-AIDS clinical events. The search for a cure therefore remains a high priority for clinicians, investigators and patients. To date, only a single individual (a Mr Timothy Ray Brown, known as the “Berlin” patient) has had apparent cure of HIV infection. In his case, the patient underwent allogeneic hematopoietic stem cell transplantation for treatment of acute myelogenous leukaemia using cells from a donor homozygous for a deletion in the CCR5 gene. Although ART was stopped at the time of transplantation, HIV did not rebound and has remained undetectable during more than seven years of follow-up. Attempts to repeat this approach in other patients have been unsuccessful to date. Current efforts at eradicating HIV infection or inducing long-term ART-free remission include activating HIV transcription in latently infected CD4+ T-cells, enhancing HIV-specific immunity in order to target and destroy cells harbouring latent infectious proviruses and employing cell-based therapies using genetically modified CD4+ T-cells or hematopoietic stem cells. Several exploratory pilot studies are underway with each of these approaches. Major challenges include the difficulty of quantifying the HIV reservoir, the uncertain safety of the experimental treatments under study and the need to balance the risk of these interventions against the generally well-tolerated and proven efficacy of long-term ART. Recent progress towards a cure presented at the 2016 CROI in Boston will be discussed.

# Sub article: Hepatitis C eradication: is it possible?

**DOI:** 10.7448/IAS.19.2.21012

**Published:** 2016-04-28

**Authors:** Michael Saag

**Affiliations:** Division of Infectious Disease, University of Alabama at Birmingham, Birmingham, AL, USA

## Abstract

Over the last five years, the release of multiple direct acting antiviral (DAA) agents has created a therapeutic revolution in the world of hepatitis C (HCV) treatment. These novel, short-course (12 week) all-oral regimens can routinely cure HCV infection in the vast majority of patients, regardless of genotype or liver fibrosis stage, in essence replacing completely the use of injectable peginterferon and its attendant side effects. Based on these striking developments, a clear message is emerging that virtually every patient with chronic HCV should be treated… Now! If such a policy is enacted, we can eradicate HCV within a decade or two, resulting in a marked reduction in disease burden and eliminating all new infections. Whether we achieve this aspirational goal depends on our collective will and creative solutions to access clinics prepared to treat HCV and solutions to access medicines, most of which are priced beyond the reach of many health systems. Several emerging concepts suggest we can begin to achieve our goal of eradication and the barriers can be overcome. The solutions are embedded in standard approaches of public health epidemiology, health policy, provider education and creative negotiations. Eradication is possible. It is up to us to make it so.

# Sub article: Where is the HIV pipeline going (and does it matter)?

**DOI:** 10.7448/IAS.19.2.21013

**Published:** 2016-04-28

**Authors:** Roy Gulick

**Affiliations:** Division of Infectious Diseases, Weill Cornell Medicine, New York, NY, USA

## Abstract

There are 29 antiretroviral drugs and five one-pill, once-daily oral formulations approved for the treatment of HIV infection. Despite their demonstrated benefits, some antiretroviral regimens may be inconvenient, toxic and/or have suboptimal virologic activity, particularly against drug-resistant strains. Thus, newer compounds and formulations are needed that continue to improve convenience and tolerability, reduce side effects and toxicity, and improve antiretroviral activity against drug-resistant viruses. There are a number of investigational antiretroviral agents currently in development, both in existing classes and new mechanistic classes. Tenofovir alafenamide is a new pro-drug of tenofovir that completed phase 3 testing and demonstrates potent virologic activity and reduced renal and bone toxicity. Doravirine is an investigational NNRTI currently in phase 3 clinical trials that has potential for fewer drug–drug interactions and activity against NNRTI-resistant viruses. Cabotegravir is an investigational integrase inhibitor in phase 3 clinical trials that can be given in a parenteral form with a long half-life supporting every 2 to 3 month dosing. Newer antiretroviral agents may exploit viral or cellular targets with new mechanisms of action. These include the investigational oral small-molecule CD4 attachment inhibitor, BMS-663068, that currently is in phase 3 clinical trials, and the investigational oral HIV maturation inhibitor, BMS-955176, that currently is in phase 2b clinical trials. Additional investigational agents, both in existing and newer antiretroviral mechanistic classes, and newer drug formulations of existing drugs are in development. Continued progress in HIV drug development will help improve the clinical care of patients living with HIV infection.

# Sub article: PrEP and implementation: a work in progress

**DOI:** 10.7448/IAS.19.2.21014

**Published:** 2016-04-28

**Authors:** Kenneth Mayer

**Affiliations:** The Fenway Institute, Fenway Health, Boston, MA, USA

## Abstract

Multiple randomized, controlled trials of oral tenofovir disoproxil fumarate with or without co-formulated emtricitabine (TDF/FTC) used for pre-exposure prophylaxis (PrEP) in high-risk people have demonstrated high levels of protective efficacy among those who were adherent. PrEP has been found to be protective in men who have sex with men (MSM), young heterosexuals, transgender women and injection drug users, although suboptimal medication adherence in some studies of young African women led to inconclusive results. TDF/FTC has been generally safe and well tolerated, though the potential for nephrotoxicity requires ongoing clinical monitoring. Statistically significant, reversible changes in bone mineral density have been observed, though the findings have not been associated with clinical disease. Concerns have been raised regarding the potential for behavioural disinhibition, but the reality is that most PrEP users have had long-standing low levels of condom use, so it is more accurate to conclude that PrEP is associated with ongoing risk maintenance. Highly adherent PrEP users are at increased risk for other sexually transmitted diseases (STDs) if condoms are not routinely used, so quarterly STD screening is warranted. Pharmacological studies and *post hoc* analyses of recent trials suggest that for men who have sex with men, there may be a level of pharmacological forgiveness, because of high concentrations of tenofovir and FTC in rectal mucosa, so that less than daily dosing can be highly effective. One study found that MSM who took two TDF/FTC doses within 24 h of condomless intercourse and a pill a day for two days after achieved 86% protection. However, because of relatively lower concentrations in cervicovaginal mucosa, it is advisable that women be highly adherent to a daily TDF/FTC PrEP regimen. Other approaches to antiretroviral chemoprophylaxis are under study. Studies of antiretroviral-containing vaginal rings and injectable PrEP are underway. Some regulatory bodies concerned about the robustness of the approach in the setting of suboptimal adherence, potential behavioural disinhibition, selection for drug resistance and the costs of prophylaxis in constrained economic settings. However, evidence from demonstration projects in multiple settings, including projects in Brazil, Australia, South Africa, Kenya and the United States, suggest that individuals who self-select to use PrEP are usually adherent and that the regimen is protective and well tolerated. PrEP will continue to evolve, but its importance as another tool to arrest HIV transmission continues to grow.

# Sub article: HAART for advanced patients. Why, what and how?

**DOI:** 10.7448/IAS.19.2.21015

**Published:** 2016-04-28

**Authors:** Omar Sued

**Affiliations:** Clinical Research, Fundación Huésped, Buenos Aires, Argentina

## Abstract

Late presenters represent a significant proportion of HIV-diagnosed patients. The factors that contribute to their late diagnosis might also contribute to poor adherence after initiating treatment, including lack of social support, active drug use or lack of knowledge about HIV and the benefits of highly active antiretroviral therapy. HIV diagnosed at advanced stage is associated with several adverse outcomes including clinical progression, blunted immune recovery after treatment, greater risk of drug toxicity and increased risk of immune reconstitution inflammatory syndrome. Randomized studies in patients presenting with opportunistic infections, including trials involving patients with pulmonary TB, have demonstrated the benefit of initiating treatment early, although controversy remains regarding the optimal time for initiating ART in patients with meningeal TB or meningeal cryptococcosis. A significant proportion of advanced patients will require intensive care. Maintaining or initiating ART during an intensive care unit admission presents distinct challenges related to drug distribution, drug doses, drug interactions and antiretroviral-associated toxic effects. Finally, randomized trials have evaluated the efficacy of different NNRTI and PI combinations in advanced patients, while studies exploring other combinations are underway.

# Sub article: Comprehensive care for elderly HIV-positive patients

**DOI:** 10.7448/IAS.19.2.21016

**Published:** 2016-04-28

**Authors:** Nestor Sosa

**Affiliations:** The Gorgas Memorial Institute for Health Studies, Panama City, Panama

## Abstract

With the success of antiretroviral therapy, more patients with HIV are living longer, and thus, a greater proportion of people living with HIV are older than 50 years of age. Besides, new HIV infections continue to occur among older adults, and late presentation, delayed diagnosis and rapid progression to AIDS have been reported in this age group. Healthcare providers should concentrate not only on screening for HIV, preventing the progression to AIDS and treating opportunistic infections, but also on the comprehensive management of this ageing patient population. There is evidence that older HIV-positive subjects have more comorbidity, are frequently treated with polypharmacy and are disproportionally affected by neurocognitive impairment. Conditions like osteoporosis, diabetes, cardiovascular disease, cancer, chronic renal disease and hyperlipidaemia are common among older adults living with HIV. Selection of antiretroviral therapy, attention to drug–drug interactions, anticipating and preventing the exacerbation of pre-existing comorbid conditions are issues that required special attention in the care of elderly HIV-positive individuals. We will review the evidence supporting some of the recommendations on how to treat older adults with HIV infection, and we will remark some of the peculiarities of the HIV infection in the elderly.

# Sub article: Best practices for monitoring of HIV virologically suppressed patients

**DOI:** 10.7448/IAS.19.2.21017

**Published:** 2016-04-28

**Authors:** Ricardo Sobhie Diaz

**Affiliations:** Infectious Diseases Division, Paulista School of Medicine, Federal University of São Paulo and Retrovirology Laboratory, São Paulo, Brazil

## Abstract

As the efficacy of antiretroviral treatment is maximized over time with the use of more potent, convenient and better tolerated drugs, and as a consequence of the test and treat era, there will be a growing number of virologically suppressed HIV-positive individuals over time. A number of virologically suppressed patients have been also under antiretroviral treatment for long periods, and individuals are growing older with emerging age-related comorbidities and polypharmacy. Monitoring laboratory tests for HIV antiretroviral treatment have been evaluated for this new era to come, and new questions naturally emerge. How frequently should HIV viral loads monitoring be performed for long-term suppressed patients? How to manage low-level viraemia among these individuals, and what is the real significance for low-level viraemia among those patients? Is CD4+ T-cell counts monitoring necessary among virologically suppressed individuals? Are genotyping or tropism tests on peripheral blood mononuclear cells of use as tool for antiretrovirals switch or antiretroviral de-intensification? Are cell activation markers promising to design strategies in order to mitigate chronic HIV-related inflammation? Blood tests for safety should also be carefully strategized in the future. Toxicities may emerge due to long-term use of antiretrovirals, age-related frailties and organ/tissues degeneration, and antiretroviral interactions with polypharmacy. It is also conceivable that long-term antiretroviral suppression may facilitate cancer emergency, specially lymphoma, due to the clonal expansion of lymphocytes with HIV integration in cancer genes [1,2].

## References

[1] Maldarelli F, Wu X, Su L, Simonetti FR, Shao W, Hill S (2014). HIV latency: Specific HIV integration sites are linked to clonal expansion and persistence of infected cells. Science.

[2] Wagner TA, McLaughlin S, Garg K, Cheung CY, Larsen BB, Styrchak S (2014). HIV latency: Proliferation of cells with HIV integrated into cancer genes contributes to persistent infection. Science.

# Sub article: Ageing and HIV: from bench to clinic

**DOI:** 10.7448/IAS.19.2.21018

**Published:** 2016-04-28

**Authors:** Peter Hunt

**Affiliations:** HIV/AIDS Division, University of California San Francisco, San Francisco, CA, USA

## Abstract

While HIV-positive individuals with access to modern antiretroviral therapy (ART) have experienced a dramatic improvement in life expectancy, they remain at higher risk than the general population for morbidity and mortality, particularly from non-AIDS complications typically associated with ageing. While lifestyle factors (e.g., smoking, illicit drug use and obesity) as well as ART toxicities likely play a role, it is now well recognized that abnormal immune activation and inflammation persist in many ART-suppressed individuals, including those that restore normal CD4+ T-cell counts, and that the extent of these immunologic defects strongly predicts morbidity and mortality from non-AIDS conditions. Interestingly, while some of the immunologic predictors of morbidity and mortality overlap with those of ageing, many are unique to HIV infection, suggesting distinct interventional targets. Multiple causes of the persistent inflammatory state in treated HIV infection have been proposed including HIV persistence, microbial translocation, cytomegalovirus and other prevalent co-infections. While earlier initiation of ART appears to be beneficial in reducing the inflammatory state and in reducing some morbidities, and some commonly used medications with anti-inflammatory properties (e.g., statins) have shown some promise in pilot studies, there is a clear need for effective interventions to reverse persistent immune activation in this setting. These issues will become increasingly important as the HIV epidemic gets older, particularly in resource-limited settings, where the vast majority of HIV-positive individuals live.

# Sub article: What the HIV-positive patients dying from? Data from across the region

**DOI:** 10.7448/IAS.19.2.21019

**Published:** 2016-04-28

**Authors:** Paco F Belaunzarán Zamudio

**Affiliations:** National Institute of Medical Sciences and Nutrition, Mexico City, Mexico

## Abstract

The expansion of universal access programmes worldwide has dramatically reduced morbidity and mortality, albeit in a different manner regionally. While the HIV epidemic in high-income countries has gone through a clear epidemiologic transition in which cardiovascular, non-AIDS neoplasias and chronic non-transmissible diseases have become the predominant cause of death among people living with HIV, limited data from Latin America and the Caribbean region suggest a more modest and apparently earlier phase of the transition in which most people with HIV still die of potentially preventable opportunistic infections early after diagnosis. Non-AIDS associated causes of death are increasing heterogeneously throughout the region but are not yet predominant. The information available about causes of death among people living with HIV at the national level in most countries in Latin America and the Caribbean is scarce. National and regional data for Brazil, and regional or single-centre published information for several countries in Latin America suggest that the impact of the expansion of universal access to antiretroviral therapies on mortality has been heterogeneous across the region and has decreased mortality the least among women. Evidence from Brazil shows that chronic non-communicable diseases have steadily increased as a cause of death in the last decade but tuberculosis and endemic transmissible diseases (e.g. histoplasmosis and visceral leishmaniasis) continue to kill a very high proportion of people living with HIV. Evidence from other countries appears to confirm this pattern happening at different rates regionally. The high prevalence of late diagnosis, shortcomings of health systems, maturity of expanded access programmes and ageing of the epidemic are most likely shaping this transition.

# Sub article: Viral load and CD4+ T-cell count dynamics in a large cohort of individuals with primary HIV-1 infection in Brazil

**DOI:** 10.7448/IAS.19.2.21020

**Published:** 2016-04-28

**Authors:** Patrícia Lima Hottz, Antonio Guilherme Fonseca Pacheco, Karine Milani da Silva, Natalia Cerqueira, Mariana Mellilo Sauer, Esper Georges Kallás, Mauro Schechter

**Affiliations:** 1 Hospital Universitário Clementino Fraga Filho/Universidade Federal do Rio de Janeiro, Infectious Diseases, Rio de Janeiro, Brazil; 2 Fundação Oswaldo Cruz (Fiocruz), PROCC, Rio de Janeiro, Brazil; 3 School of Medicine/University of São Paulo, Division of Clinical Immunology and Allergy, São Paulo, Brazil; 4 Projeto Praça Onze/Hospital Escola São Francisco de Assis/Universidade Federal do Rio de Janeiro, Infectious Diseases, Rio de Janeiro, Brazil

## Abstract

**Introduction:**

There is limited information about the natural history of HIV-1 infection in Latin American countries [1]. This study describes the trajectories of the plasma viral load and CD4+ T-cell (CD4) counts in individuals with documented primary HIV-1 infection in Rio de Janeiro and Sao Paulo, between 1989 and 2013. This is the largest study ever conducted in Brazil and one of the few in Latin America that have investigated the natural trajectories of these important predictors of HIV-1 disease progression.

**Methods:**

This retrospective cohort study analyzed viral load and HIV-RNA CD4 counts of 307 individuals with documented primary or recent HIV-1 infection (within 12 months) in Rio de Janeiro and Sao Paulo. Follow-up was censored at the last visit or at the start of antiretroviral therapy. The impact of demographic and clinical variables such as gender, age at seroconversion, race and occurrence of symptoms compatible with the acute retroviral syndrome on the dynamics of viral load and CD4 counts was analyzed. Generalized additive mixed effects models with Poisson response were used in the modelling of CD4 counts and viral load measures. The time between seroconversion and CD4 decline to less than 350 cells/mm^3^ was analyzed by Kaplan–Meier survival curves. The Cox model was used to estimate the effects of the various strata.

**Results:**

The median initial CD4 counts and viral loads were 512.5 (range 390.8 to 684.2) cells/mm^3^ and 4.31 (range 3.61 to 4.85) log_10_ cps/mL, respectively. The median time of CD4 decline to <350 cells/mm^3^ was 588 (interquartile range: 458 to 864) days. Non-white individuals were at an increased risk of a faster decline of CD4 counts (Figure 1). Older age was a risk factor for lower CD4 counts at seroconversion and to faster declines to CD4 counts to <350 cells/mm^3^. Additionally, individuals with the acute retroviral syndrome evolved with higher viral loads, which in turn were associated with a faster decline of CD4 counts. There were no differences between genders in the median time of CD4 decline to <350 cells/mm^3^.

**Conclusions:**

Due to the current recommendations for treatment of HIV-1 infection regardless of CD4+ T-cell count, it is unlikely that similar studies will be conducted in the future, which increases its importance, particularly for the discussion of the cost-effectiveness of various possible prioritization strategies for testing and initiation of antiretroviral therapy.

## References

[1] Djomand G, Duerr A, Faulhaber JC, Struchiner CJ, Pacheco AG, Barroso PF (2006). Viral load and CD4 count dynamics after HIV-1 seroconversion in homosexual and bisexual men in Rio de Janeiro, Brazil. J Acquir Immune Defic Syndr.

# Sub article: ART initiation rates and first-line regimens in Latin America remain a challenge for WHO recommendations

**DOI:** 10.7448/IAS.19.2.21021

**Published:** 2016-04-28

**Authors:** Isabel Cassetti, Pablo Parenti, William Lenis, Rosa Teran, Alberto Castillo, Ana Belen Arauz, Miguel Morales

**Affiliations:** 1 Latin American HIV Workshop Study Group, AIDS, Buenos Aires, Argentina; 2 Latin American HIV Workshop Study Group, AIDS, Rosario, Argentina; 3 Latin American HIV Workshop Study Group, AIDS, Cali, Colombia; 4 Latin American HIV Workshop Study Group, AIDS, Quito, Ecuador; 5 Latin American HIV Workshop Study Group, AIDS, Panama City, Panama; 6 Latin American HIV Workshop Study Group, AIDS, Caracas, Venezuela

## Abstract

**Introduction:**

WHO Consolidated Guidelines released in November 2015 recommend antiretroviral therapy (ART) initiation in all adults living with HIV regardless of clinical stage and at any CD4 count. The preferred first-line regimen is TDF+3TC (FTC)+EFV with NVP, EFV 400 mg and DTG as alternative regimens as well as AZT+3TC in place of TDF+3TC. Latin American countries follow local specific guidelines, each one recommending different approaches for time and ART combinations for ART initiation. The aim of this study is to determine how often ART is initiated in new HIV cases and what antiretroviral combinations are used in clinical practice in countries from Latin America.

**Materials and methods:**

The Latin American Workshop Study Group is an expanding network of 38 HIV Care Centers from 11 countries of South America, Central America, the Caribbean and Mexico with clinical data from 73,431 patients up to September 2015. We identified 9979 cases of ART initiation in 2013 to 2014 in eight countries. Statistical analysis by chi-square test and confidence intervals.

**Results:**

Globally, 81% of newly diagnosed HIV cases initiated ART during the first year of follow-up. Mexico, Dominican Republic and Argentina were the only countries with rates of ART initiation of 90% or higher, while rates of ART initiation were 64% to 74% in most South American countries. Only 16% of patients initiated TDF+3TC/FTC+EFV. AZT+3TC is still the preferred backbone (52.2%), while TDF+3TC/FTC is the second preferred backbone (24.1%) with large differences in NRTI use between countries: less than 25% in Costa Rica and Peru but over 90% in Ecuador and Mexico. EFV is the third drug in 65.4% of therapies initiated in 2013 to 2014, protease inhibitors in 18.3% of cases and Raltegravir in 2.4%.

**Conclusions:**

In Latin America, some countries adopted ART initiation regardless of CD4 count, while most South American countries show ART initiation rates around 70%. Regimens selected for first-line therapy are diverse between countries, and the WHO-recommended regimen is not preferred in most countries. AZT+3TC is still the preferred backbone, protease inhibitors are frequently used as third drug and a few patients initiate integrase inhibitors. Standardization of first-line regimens is warranted in Latin America.

# Sub article: Alarming increase of transmitted drug resistance to first-line HIV regimen in Aruba

**DOI:** 10.7448/IAS.19.2.21022

**Published:** 2016-04-28

**Authors:** L Marije Hofstra, Elena Sanchez Rivas, Leonie Bank, Tania Mudrikova, Tulio De Oliveira, Jaclyn de Kort, Karina Kelly, Annemarie Wensing

**Affiliations:** 1 University Medical Center, Medical Microbiology and Virology, Utrecht, Netherlands; 2 Horacio E. Oduber Hospital, Internal Medicine, Oranjestad, Aruba; 3 University Medical Center, Internal Medicine and Infectious Diseases, Utrecht, Netherlands; 4 University of KwaZulu-Natal, Africa Centre for Health and Population Studies, Mtubatuba, South Africa

## Abstract

**Introduction:**

The HIV epidemic in Aruba is growing. Recently, genotypic resistance testing in clinical practice showed a worrying increase of the resistance mutation K103N in reverse transcriptase, which generates high-level resistance to the current first-line NNRTI regimen in Aruba. We set out to investigate whether these patients were therapy-naïve or treated.

**Methods:**

HIV-1 pol genotypic sequence analysis was performed for 127 patients of the Horacio E. Oduber Hospital between 2010 and 2015. Baseline testing was performed in 100 patients for 27 patients, resistance testing was only done during therapy failure. Transmitted drug resistance was determined using the WHO list [1]. Phylogenetic analysis included 147 subtype-B sequences from Aruba (100 baseline, 47 failure), subtype-B baseline sequences from the Netherlands (*n*=220) and the top-10 most similar sequences selected via BLAST (*n*=145). A maximum-likelihood phylogenetic tree was constructed (GTR-model, 1000 replicates, MEGA6). Clinical and virological data were retrieved from patient records.

**Results:**

Most patients were male, with a median age of 40 years, from Aruba, who indicated to be infected in Aruba through sexual contact (Table 1). Twenty per cent of patients presented with an AIDS-defining illness. Transmitted drug resistance to NRTIs (*n*=2) or protease inhibitors (*n*=2) was low, but the prevalence of K103N in newly diagnosed patients who received baseline resistance testing was 32% overall and increased from 33% in 2014 (8/24) to 48% in 2015 (12/25). K103N was also detected in 60% (16/27) of patients who were only tested during therapy failure, although two patients were not exposed to NNRTI regimens. Phylogenetic analysis revealed that K103N was transmitted to therapy-naïve patients via at least six distinct introductions. One introduction resulted in a large cluster of 36 men. Within this cluster, 73% (24/33) of therapy-naïve patients had K103N. Of those in the cluster without K103N at baseline, four patients presented late, thus reversion of K103N to wild type in the absence of drug pressure cannot be excluded. The majority of this cluster was diagnosed in 2014/2015 (*n*=21), of which eight patients had a recent infection based on a recent negative test.

**Conclusions:**

The prevalence of the resistance mutation K103N in therapy-naïve patients in Aruba has increased to an alarming level. This is only partly explained by clustered transmission. Although baseline resistance testing has now been implemented routinely, detection of K103N may still fail due to reversion to wild type. In a setting with such high rates of transmitted resistance, it should be considered to adapt to the standard first-line NNRTI regimen.

**Abstract O225–Table 1:** Baseline characteristics of study population

		Total (*n*=127)	Naïve patients (*n*=100)	Treated patients (*n*=27)
Sex	Male	103 (81.1)	87 (87.0)	16 (59.3)
Age at diagnosis	Median (IQR)	40 (30 to 50)	47 (31 to 50)	34 (25 to 43)
Year of diagnosis	Median (IQR)	2012 (2009 to 2015)	2014 (2012 to 2015)	2005 (2000 to 2011)
Country of origin	Aruba	74 (58.3)	59 (59.0)	15 (55.6)
	Colombia	18 (14.2)	15 (15.0)	3 (11.1)
	Venezuela	10 (7.9)	5 (5.0)	5 (18.5)
	Netherlands	5 (3.9)	5 (5.0)	0
	Other	20 (15.7)	16 (16.0)	4 (14.8)
Route of transmission	MSM	69 (54.3)	59 (59.0)	10 (37)
	Heterosexual	46 (36.2)	23 (23.0)	13 (48.1)
Country of infection	Aruba	104 (81.9)	85 (85.0)	19 (70.4)
	Outside Aruba	11 (8.7)	7 (7.0)	4 (14.8)
	Unknown	9 (7.1)	6 (6.0)	3 (11.1)
Log HIV RNA load	Median (IQR)	5.0 (4.0–6.0)	5.0 (4.0–6.0)	4.08 (2.58–5.58)
CD4 count	Median (IQR)	310 (91–529)	305 (80–530)	315 (100–530)
AIDS at diagnosis		26 (20.5)	23 (23.0)	3 (11.5)
Subtype B		120 (94.5)	95 (95.0)	25 (92.6)

## References

[1] Bennett DE, Camacho RJ, Otelea D, Kuritzkes DR, Fleury H, Kiuchi M (2009). Drug resistance mutations for surveillance of transmitted HIV-1 drug-resistance: 2009 update. PLoS One.

# Sub article: High loss to follow-up among patients with HIV diagnosis during hospitalization

**DOI:** 10.7448/IAS.19.2.21082

**Published:** 2016-04-28

**Authors:** Mariana Kundro, Sofie Terwel, Guillermo Viloria, Javier Toibaro, Marcelo Losso

**Affiliations:** Hospital JM Ramos Mejia, HIV Unit, Buenos Aires, Argentina

## Abstract

**Introduction:**

There are limited data exploring the retention in care of patients who are diagnosed with HIV in circumstances of hospitalization. Our objective was to assess the rates and factors associated with lost to follow-up (LFU) in patients that were diagnosed in our centre during a hospitalization in comparison with individuals who came voluntarily for testing.

**Materials and methods:**

All adult patients with a new HIV diagnosis between January 2002 and July 2014 were included. LFU was defined as patients who did not attend any visit and retention in care as patients with two or more visits in the first year after HIV diagnosis. Moreover, we also examined the proportion of patients that attended only one visit within the year after diagnosis. Logistic regression models were used to evaluate factors associated with LFU.

**Results:**

A total of 1092 patients were included (27.7% female, 10.1% transgender), of whom 244 (22.3%) were diagnosed in circumstances of hospitalization (68.5% because of AIDS defining illness). About 10.2% (25/244) of patients died during hospital stay and 6.3% (14/219) were rehospitalized before six months from hospital discharge and were excluded from final analyses. Median CD4+ was 110 cell/mm^3^ (IQR 37–246) for hospitalized patients versus 372 (IQR 235–549) for ambulatory individuals. About 19.2.% of patients with HIV diagnosis during hospitalization were LFU, 13% had at least one visit after discharge and 67.1% were retained in care, whereas only 1.92% of ambulatory patients were LFU, 24.2% attended one visit and 73.8% were retained in care. Factors associated with LFU were cocaine use (OR 2.76; CI 95% 1.07 to 7.12; p: 0.03), alcohol abuse (OR 6.06; CI 95% 1.48 to 24.8; *p*=0.01) and HIV diagnosis under circumstances of hospitalization (OR 18.14; CI 95% 9.05 to 36.3; *p*<0.001), whereas sex, age and AIDS at diagnosis were not associated. In a multivariate model including sex, age, year of diagnosis, alcohol abuse, cocaine use, homosexual transmission and HIV diagnosis during hospitalization, only the last two factors remained as independent factors associated with LFU.

**Conclusions:**

The proportion of HIV-infected patients LFU after diagnosis during hospitalization is high. These results highlight the need to implement strategies focused to engage these individuals shortly after hospital discharge.

# Sub article: Elimination of MTCT in the Americas: where are we?

**DOI:** 10.7448/IAS.19.2.21023

**Published:** 2016-04-28

**Authors:** Massimo Ghidinelli

**Affiliations:** Pan American Health Organization, Washington, DC, USA

## Abstract

In 2010, PAHO Member States adopted, by resolution, the Strategy and Plan of Action for the Elimination of Mother-to-Child Transmission (EMTCT) of HIV and Congenital Syphilis (CS). Objectives of the plan are (a) to reduce mother-to-child HIV transmission rate to 2% or less; (b) to reduce the incidence of MTCT of HIV to 0.3 cases or less per 1000 live births; (c) to reduce the incidence of CS to 0.5 cases or less (including stillborn infants) per 1000 live births. The resolution called on Member States to give priority to the EMTCT of HIV and syphilis and to develop and execute national plans towards the elimination goals. Significant progress has been made by the region with 17 countries reporting data compatible with the dual elimination by the end of 2014. The rate of MTCT of HIV in Latin America and the Caribbean (LAC) decreased 50% between 2010 and 2014, from 14% to 7%. These data are in line with antiretroviral therapy coverage in HIV-positive pregnant women in LAC, which increased from 56% in 2010 to 81% in 2014. In 75% of the countries, reported MTCT transmission of HIV is under 4%. However, still 17,400 cases of CS were reported in 2014 in the Americas, at a rate of 1.3 cases per 1000 live births. On the other hand, services for HIV and syphilis are becoming firmly integrated in prenatal healthcare, and the EMTCT goals increasingly perceived as quality markers of maternal and child care services. PAHO is proposing that the well-established EMTCT platform for HIV and syphilis in the region could be used to leverage other MTCT infections relevant to public health, such as hepatitis B virus and Chagas disease in endemic areas.

# Sub article: Cuba EMTCT achievement: How Cuba got there first?

**DOI:** 10.7448/IAS.19.2.21024

**Published:** 2016-04-28

**Authors:** Maria Isela Lantero

**Affiliations:** National STI/HIV/AIDS Program, Havana, Cuba

## Abstract

The Cuban HIV/AIDS Prevention and Control Program was conceived based on well-known public health approaches applied in the country for disease prevention and control. Use was made of previous experiences in the control of sexually transmitted infections, and actions were implemented for the early diagnosis of cases, their epidemiological investigation, partner reporting, education of the population and availability of prevention, care and treatment services for those affected. Almost 30 years later, these strategies still exist and have been resized and adapted to each point in the evolution of the epidemic. Our Maternal and Infant Care programme constitutes the platform for the prevention of maternal and congenital syphilis and HIV transmission. Care comprises follow-up of the health status of pregnant women, including the performance of serological tests for syphilis and HIV for them and their sexual partners. Access to prenatal care that includes actions to prevent mother-to-child transmission of HIV, specialized HIV care, antiretroviral treatment and support for people living with HIV network is guaranteed. In the last four years, the annual rate of congenital syphilis has ranged between 0 and 0.04 per thousand live births and the annual rate of mother-to-child transmission of HIV under 2%. Considering these results, we officially requested validation by the Pan American Health Organization and the World Health Organization, and after completing each step established by the regional strategy, Cuba became the first country in the world to receive validation from the World Health Organization that it has eliminated mother-to-child transmission of HIV and syphilis.

# Sub article: Gender-specific issues for HIV-positive women

**DOI:** 10.7448/IAS.19.2.21025

**Published:** 2016-04-28

**Authors:** Sharon Walmsley

**Affiliations:** Clinical Research, Immunodeficiency Clinic, Toronto Hospital, and Department of Medicine, University of Toronto, Toronto, ON, Canada

## Abstract

More than half of the HIV-positive population are women. There is limited gender-specific research. In this lecture, I will discuss antiretroviral therapy and the special issues that women need to consider in terms of drug interactions with oral contraceptive agents, risk during pregnancies and comorbidities such as osteoporosis with age. I will also discuss some of the specific issues related to HIV and women, which will include human papillomavirus (HPV) infection, the role of the HPV vaccine and other considerations for women ageing with HIV and the impact of the menopause.

# Sub article: Gender-specific issues for HIV-positive women: the regional perspective

**DOI:** 10.7448/IAS.19.2.21026

**Published:** 2016-04-28

**Authors:** Alicia Piñeirúa

**Affiliations:** Clínica Especializada Condesa Iztapalapa, Mexico City, Mexico

## Abstract

According to UNAIDS, 31% of adults living with HIV in Latin America are women. HIV transmission among Latin American women occurs mostly through heterosexual contact (96.7%), and in the vast majority of cases, the source of infection is the woman's stable partner. The existing scientific literature on HIV in Latin American women has primarily focused on populations of women who are considered to be “high risk”: migrants, sex workers, drug users and women in prison. Despite evidence to the contrary, monogamous women with stable partners are often not considered to be at risk for HIV. Timely diagnosis among women is not only crucial in terms of their own prognosis, but it also implies the first step for prevention of HIV mother-to-child transmission. However, HIV testing coverage during pregnancy in the region is currently around 66%, and in some countries declines to proportions as low as 30%. On the other hand, some studies conducted in Latin American countries have shown that women are often diagnosed in advanced stages of HIV infection, many of them due to missed opportunities of timely diagnosis. Women's vulnerability to HIV infection involves diverse factors, including biological as well as socio-economic and policy related aspects. Some of these factors may also represent barriers to healthcare access, linkage to care and antiretroviral treatment. Many countries still lack tailored interventions and strategies for HIV prevention and care in female populations. Interdisciplinary approaches are needed to adequately take care of this particular epidemic.

# Sub article: Improving prevention of mother-to-child transmission of HIV outcomes in Haiti from 1999 to 2014: monotherapy to Option B+

**DOI:** 10.7448/IAS.19.2.21027

**Published:** 2016-04-28

**Authors:** Marie Marcelle Deschamps, Deanna Jannat-Khah, Jerry Bonhomme, Vanessa Rouzier, Pierrot Julma, Christian Perodin, Jean William Pape, Margaret McNairy

**Affiliations:** 1 Centres GHESKIO, Women's Health, Port Au Prince, Haiti; 2 Weill Medical College of Cornell University, International Medicine, New York, USA; 3 Centres GHESKIO, Maternal Health, Port Au Prince, Haiti; 4 Centres GHESKIO, Pediatrics, Port Au Prince, Haiti; 5 Centres GHESKIO, Data Management, Port Au Prince, Haiti; 6 Centres GHESKIO and Weill Medical College, International Medicine, Port Au Prince, Haiti

## Abstract

**Introduction:**

Haiti has one of the highest vertical transmission rates in the Americas. We assessed outcomes of prevention of mother-to-child transmission (PMTCT) care across changes in national guidelines including Option B+, at the largest PMTCT programme in Port-au-Prince over the past decade.

**Methods:**

Data from all HIV-positive women enrolling in PMTCT care at GHESKIO from January 1999 through July 2014 were included. PMTCT cascade steps included enrolment in PMTCT care, receipt of antiretroviral (ARV) prophylaxis or antiretroviral therapy (ART) prior to delivery, infant born alive, infant enrolment in PMTCT care, infant receipt of HIV diagnostic test and infant HIV diagnosis. Outcomes were compared across programme period 1 (1999 to 2004, monotherapy), 2 (2005 to 2009, dual therapy), 3 (2010 to 2012, triple therapy) and 4 (Oct 2012 to 2014, Option B+). Median time from PMTCT enrolment to care and lost to follow up were compared across programme periods.

**Results:**

A total of 3737 HIV-positive women accounted for 4665 unique pregnancies. Median age was 27 years; 66% were married, and 76% earned <$1/day. Median maternal CD4 at time of PMTCT enrolment was 494 cells/uL (IQR 328–691). 3501 pregnancies (75%) received ARV/ART prior to delivery, of whom the proportion receiving ART increased from 6% to 100% in periods 1 to 4. Median time to ARV/ART start after mother enrolment in PMTCT decreased from 24 days in periods 1 to 2 to 0 days in period 4. Among 4665 pregnancies, 3414 (73%) had an infant born alive, of whom 3218 (94%) enrolled in PMTCT care. A total of 2955 (92%) infants had complete HIV testing with 161 HIV-infected infants for a 5.5% transmission rate (varying from 9.9%, 4.6%, 5.8% and 3.6% in periods 1 to 4, respectively). Transmission during 2010, after the historic earthquake, was 8.5% as compared to 4.2% in 2009 and 4.6% in 2011. The greatest loss of patients across all periods was among women prior to infant delivery, with median time to loss decreasing from 105 to 31 days in periods 1 to 4. A total of 18% (178/1004) of these women who were lost to follow up had only one PMTCT visit prior to loss. Among 161 HIV-infected infants at 12 months after birth, 71 (44%) were alive in care, 38 died (23%) and 52 lost (32%).

**Conclusions:**

Transmission rates have dramatically declined in this PMTCT programme in Haiti over the past decade, particularly under the era of Option B+. Interventions are needed to retain women and their infants within and after delivery period in order to achieve HIV elimination.

# Sub article: Paediatric HIV cascade: from maternal PMTCT to infant ART initiation – improvements over 15 years of scale up in Haiti

**DOI:** 10.7448/IAS.19.2.21028

**Published:** 2016-04-28

**Authors:** Vanessa Rouzier, Marie Marcelle Deschamps, Deanna Jannat-Khah, Jerry Bonhomme, Pierrot Julma, Christian Perodin, Jean William Pape, Margaret McNairy

**Affiliations:** 1 GHESKIO Centers, Pediatrics, Port-au-Prince, Haiti; 2 GHESKIO Centers, Women's Health, Port-au-Prince, Haiti; 3 Weill Cornell Medical College, Global Health, New York, USA; 4 GHESKIO Centers, Obstetrics, Port-au-Prince, Haiti; 5 GHESKIO Centers, IT, Port-au-Prince, Haiti; 6 GHESKIO Centers, Executive Director, Port-au-Prince, Haiti

## Abstract

**Introduction:**

Haiti has one of the highest vertical transmission rates in the Americas. We assessed HIV-infected infant outcomes across the paediatric HIV care cascade over a decade of changes in national guidelines in the largest prevention of mother-to-child transmission (PMTCT) and paediatric HIV programme in Port-au-Prince.

**Materials and methods:**

Data from all HIV-infected infants born to women enrolling in PMTCT care at GHESKIO clinic from January 1999 through December 2014 were included. Prior to 2009, HIV infection was confirmed in <18-month olds by two positive HIV-RNA (EasyQ) assays, or one HIV-RNA plus one positive p24 antigen assay, or with a positive HIV ELISA in children >18 months old. Starting in 2009, HIV DNA PCR was diagnostic. Retention across the infant HIV care cascade was compared before and after changes in national guidelines for infant ART eligibility from 1999 to 2008 and from 2009 to 2014.

**Results:**

A total of 3737 HIV-infected women accounted for 4665 unique pregnancies, with 2955 infants completing HIV testing. A total of 161 (5.5% transmission) were HIV-infected. Sixty per cent were female; 12% weighed <2500 g at birth and 50% were delivered in a hospital setting. Median maternal age was 27 years; median maternal CD4 count at PMTCT enrolment was 399 cells/L (IQR 288–576); and 21% had WHO Stage III/IV symptoms. From 1999 to 2008, 71 HIV-infected infants were diagnosed and 69 (96%) returned for test results, of whom 44 (64%) had CD4 testing. In this period, 38 were eligible (86%) and 19 (50%) initiated ART. Seven eligible infants died prior to ART initiation. Among the 6 ART-ineligible patients, 3 remain alive in care with CD4 >1000 cells/L and 3 are lost to programme. From 2009 to 2014, 89 HIV-infected infants were diagnosed and 78 (88%) returned for test results, of whom 77 (99%) had CD4 testing. All 77 (100%) were ART-eligible and 76 (99%) initiated ART.

**Conclusions:**

Performance of the infant HIV care cascade since 2009 illustrates that improvements in infant diagnosis, prompt initiation on antiretroviral therapy, and retention in care can be achieved. Point of care infant diagnostics could help reduce loss prior to infant test results return, currently the largest gap in the cascade.

# Sub article: P001 Use of social networking applications (apps) and meeting sites in patients with acute HIV infection in a specialized clinic in Mexico City

**DOI:** 10.7448/IAS.19.2.21029

**Published:** 2016-04-28

**Authors:** Jeremy Bernardo Cruz, Adriana Harumi Hirata, Edgardo Hamid Vega, Valeria Daniela Ferreya, Carolina Rocabert, Victor Rodriguez, Andrea Gonzalez

**Affiliations:** 1 Clinica Especializada Condesa, Mental Health, Mexico City, Mexico; 2 Clinica Especializada Condesa, Coordination VIH Program Mexico City, Mexico City, Mexico

## Abstract

**Introduction:**

Acute phase of HIV infection is a crucial moment; from 2 to 3 weeks after infection, exponential growth of the virus occurs and the person is most infectious, knowing the risks involved [1,2]. We observed a significant impact of applications among men who have sex with men (MSM) and meeting places in sexual health, and use of these applications leads to a greater likelihood of having unprotected sex and spread of disease by sexual transmission [3].

**Materials and methods:**

Semistructured interview about using applications and meeting places in the last three months before diagnosis was applied. Data were collected from 2014 to 2016. Categorical variable frequencies and percentages for the means and standard deviations continuous variables were calculated.

**Results:**

Eighty subjects were reported with acute phase of HIV infection. The average age was 28.11 years (± 8.09), single 68.7% (N=55), with partner 31.3% (N=25) of which 48% had discordant couple (N=12). Years of study 13.5 (±3.04), employed 46.3% (N=37), student 20% (N=16) and 18.7% unemployed (N=15). On the sexual position, 72.5% (58) were active/passive, 15% (12) were active and 12.5% (10) were passive; 85% uncircumcised. First sexual intercourse was during 18.8 years (±17) and sexual partners (SP) along life 129.8 (±292). SP in the last three months was 8.3 (±21.2). 56.25% (N=45) of the total sample reported no condom use with sexual partners an average of 4.3 with no condom was used. The main cause of non-use was impulsiveness in 41.25% (N=33) followed by trust between partners 32.5% (N=26); 28.8% (N=25) reported using applications. Grindr was used in 60.8% (N=14), Manhunt and Facebook 13.4% (N=3) each; 52.1% (N=12) reported using more than one application and 17.4% (N=4) more than two. The average contact pairs obtained was 15.21 (±29.7). With regard to meeting places 37.5% (N=30) goes to meeting places; 50% (N=15) goes to more than one meeting place and 13.3 (N=4) are going to more than two. Attending vapours/sauna 50% (N=15) and orgies 26.6% (N=8).

**Conclusions:**

This is the first study using applications in a population of newly infected patients in Mexico City. Knowing risk sites allows preventive policies and procedures to risk behaviours.

## References

[1] Nicola Z, Pilcher C (2007). Diagnosis in management of acute HIV infection. Infect Dis Clin N Am.

[2] Van Kesteren N, Hospers H, Kok G (2007). Sexual risk behavior among HIV positive men who have sex with men: a literature review. Patient Educ Couns.

[3] Lehmiller J, Loerger M (2014). Social networking smartphone applications and sexual health outcomes among men who have sex with men. PLoS One.

# Sub article: P002 Sexual risk behaviours among MSM with acute HIV infection in a specialized clinic in Mexico City

**DOI:** 10.7448/IAS.19.2.21030

**Published:** 2016-04-28

**Authors:** Adriana Harumi Hirata, Jeremy Bernardo Cruz, Edgardo Hamid Vega, Valeria Daniela Ferreya, Carolina Rocabert, Victor Rodriguez, Andrea Gonzalez

**Affiliations:** 1 Clinica Especializada Condesa, Mental Health, Mexico City, Mexico; 2 Clinica Especializada Condesa, Coordination VIH Program Mexico City, Mexico City, Mexico

## Abstract

**Introduction:**

Acute phase of HIV infection is a crucial moment of infection to the spread of the virus [1,2]. Weeks 2–3 after infection exponential growth of the decrease in viral load and CD4 occurs. This reported in the literature that sexual risk behaviours as traumatic sex, receptive anal intercourse, active ulcerative disease, sex work, drug use and regular multiple sexual partners. In men, the risk factors have been associated with who are not circumcised [3].

**Methods:**

Semistructured interview about risky sexual behaviour and substance use self-report in the three months prior to HIV diagnosis is applied. Categorical variable frequencies and percentages for the means and standard deviations continuous variables were calculated.

**Results:**

Eighty subjects were reported with acute HIV. The average age was 28.11 years (±8.09), single 68.7% (N=55), with partner 31.3% (N=25) of which 48% had discordant couple (N=12). Years of study 13.5 (±3.04), employed 46.3% (N=37), student 20% (N=16) and 18.7% unemployed (N=15). On the sexual position 72.5% (N=58) were reported as active/passive, 15% (N=12) as active and 12.5% (N=10) as passive. 85% uncircumcised. First sexual intercourse was during 18.8 years (±17) and sexual partners (SP) along life 129.8 (±292). SP in the last three months 8.3 (±21.2). STIs were reported in 35% (N=28), the first urinary tract infection 32.1% (N=9) and syphilis 29% (N=8). Anal bleeding during intercourse 11.2% (N=9). Intoxication with substances in coitus 23.7% (N=19), with ethanol being the first cause with 52.3% (N=11) followed by cannabis and poppers with 19% (N=4) each. 56.25% (N=45) of the total sample use no condoms with sexual partners an average of 4.3 with no condom was used. The main cause of non-use was impulsiveness in 41.25% (N=33) followed by trust between partners 32.5% (N=26).

**Conclusions:**

This is the first report in Mexico of sexual behaviours in persons with acute infection. Knowing the sexual behaviours that acute patients are having will allow us to make strategies to prevent the spread of HIV.

## References

[1] Nicola Z, Pilcher C (2007). Diagnosis in management of acute HIV infection. Infect Dis Clin N Am.

[2] Van Kesteren N, Hospers H, Kok G (2007). Sexual risk behavior among HIV positive men who have sex with men: a literature review. Patient Educ Couns.

[3] Zou H, Prestage G, Fairley CK, Grulich AE, Garland SM, Hocking JS (2014). Sexual behaviors and risk for sexually transmitted infections among teenage men who have sex with men. J Adolesc Health.

# Sub article: P003 A randomized, double-blind comparison of tenofovir alafenamide (TAF) versus tenofovir disoproxil fumarate (TDF), each co-formulated with elvitegravir, cobicistat and emtricitabine (E/C/F) for initial HIV-1 treatment: week 96 results

**DOI:** 10.7448/IAS.19.2.21031

**Published:** 2016-04-28

**Authors:** David Wohl, Shinichi Oka, Nathan Clumeck, Amanda Clarke, Cynthia Brinson, Karen Tashima, José Arribas, Antoine Chéret, Jason Brunetta, Paul Sax, Lijie Zhong, Moupali Das, Marcelo Laurido, Marshall Fordyce

**Affiliations:** 1 Infectious Diseases, University of North Carolina, Chapel Hill, NC, USA; 2 National Center for Global Health and Medicine, Tokyo, Japan; 3 Division of Infectious Diseases, Saint-Pierre University Hospital, Brussels, Belgium; 4 Brighton and Sussex Medical School, Brighton & Sussex University Hospitals NHS Foundation Trust, Brighton, UK; 5 Central Texas Clinical Research, Austin, TX, USA; 6 Alpert Medical School of Brown University, Providence, RI, USA; 7 Hospital La Paz, Madrid, Spain; 8 Tourcoing Hospital, Paris, France; 9 University of Toronto, Toronto, Canada; 10 Harvard Medical School, Boston, USA; 11 Biostatistics, Gilead Sciences, Foster City, CA, USA; 12 HIV Clinical Research, Gilead Sciences, Foster City, CA, USA; 13 Public Health and Medical Affairs, Gilead Sciences, Foster City, CA, USA

## Abstract

**Introduction:**

Two international, randomized, double-blinded, phase 3 trials in distinct regions directly compared TAF versus TDF, each co-formulated with elvitegravir/cobicistat/emtricitabine (E/C/F). At week 48, E/C/F/TAF met the primary objective of non-inferior efficacy with improved renal and bone secondary safety endpoints compared with E/C/F/TDF. We describe longer-term follow-up of efficacy, safety and tolerability endpoints through week 96.

**Materials and methods:**

Antiretroviral (ARV) naïve participants were randomized 1:1 to receive E/C/F/TAF (TAF) or E/C/F/TDF (TDF). Week 96 viral suppression (HIV-1 RNA <50 c/mL) by FDA snapshot analysis, pre-defined bone and renal safety, and tolerability endpoints are reported.

**Results:**

A total of 1733 subjects were randomized and treated: 15% women, 43% non-white and 23% viral load (VL) >100,000 c/mL. Median baseline characteristics: age 34 years, CD4 count 405 cells/µL and VL 4.58 log10 c/mL. Virologic suppression (HIV-1 RNA <50 c/mL) was 86.6% (TAF) and 85.2% (TDF) (difference 1.5%; 95%CI [−1.8, 4.8%], *p*=0.36). Viral outcomes did not vary by age, sex, race, geography or baseline CD4/VL. Mean [SD]% decrease in BMD was significantly less in the TAF group for both lumbar spine (−0.96 [3.72] vs. −2.79 [3.92], *p*<0.001) and total hip (−0.67 [3.89] vs. −3.28 [3.97], *p*<0.001). As shown in Table 1, renal safety endpoints favoured TAF. There were greater increases in lipids in the TAF arm versus TDF but no difference in rate of initiation of lipid-modifying agents (TAF: 3.8% vs. TDF: 4.4%). There were no cases of renal tubulopathy in the TAF arm versus two on TDF, including one that led to discontinuation.

**Conclusions:**

Through week 96, rates of virologic suppression were high and similarly maintained in both the TAF and TDF groups. E/C/F/TAF continued to have a statistically superior bone and renal safety profile compared with E/C/F/TDF. These longer-term data support the use of E/C/F/TAF as a safe, well-tolerated and durable regimen for initial and ongoing HIV-1 treatment.

**Abstract P003–Table 1:** Renal safety endpoints

Median (Q1,Q3) per cent change from baseline (unless otherwise noted)	E/C/F/TAF	E/C/F/TDF	Significance
eGFR Cockcroft–Gault (mL/min) (change from baseline)	−2.0 (−12.4, 9.4)	−7.5 (−17.4, 2.9)	*p*<.0001
Urine protein/Cr	−9.1 (−39.6, 36.0)	16.2 (−22.5, 81.5)	*p*<.0001
Urine albumin/Cr	−5.2 (−35.7, 30.1)	4.9 (−32.7, 60.0)	*p*<.0001
β-2 Microglobulin/Cr	−32.1 (−61.0, 4.2)	33.5 (−27.8, 230.7)	*p*<.0001
Retinol-binding protein/Cr	13.8 (−18.8, 66.1)	74.2 (10.4, 192.3)	*p*<.0001

# Sub article: P004 Influence of age on outcomes in HIV-positive adults initiating tenofovir alafenamide fumarate (TAF) versus tenofovir disoproxil fumarate (TDF) with elvitegravir, cobicistat and emtricitabine (E/C/F/TAF vs. E/C/F/TDF)

**DOI:** 10.7448/IAS.19.2.21032

**Published:** 2016-04-28

**Authors:** Jaime Andrade Villanueva, Eric Daar, Benoit Trottier, Amanda Clarke, David Parks, Cynthia Brinson, Hal Martin, Susan Guo, Sandra Friborg, Marcelo Laurido, Marshall Fordyce

**Affiliations:** 1 Hospital Civil de Guadalajara, Guadalajara, Mexico; 2 Los Angeles Biomedical Research Institute at Harbor-UCLA Medical Center, Torrance, CA, USA; 3 Centre D'excellence VIH-Its-Hepatites, Clinique Medicale L'Actuel, Montreal, QC, Canada; 4 Royal Sussex County Hospital, HIV/Gum and Clinical Trials, Brighton, UK; 5 Central West Healthcare, St. Louis, MO, USA; 6 Central Texas Clinical Research, Austin, TX, USA; 7 Clinical Research, Gilead Sciences, Foster City, CA, USA; 8 Biostatistics, Gilead Sciences, Foster City, CA, USA; 9 Clinical Operations, Gilead Sciences, Foster City, CA, USA; 10 Public Health and Medical Affairs, Gilead Sciences, Foster City, CA, USA; 11 HIV Clinical Research, Gilead Sciences, Foster City, CA, USA

## Abstract

**Introduction:**

As the HIV-infected population ages and has increasing prevalence of co-morbidities, efficacy and safety of antiretrovirals in older patients is of heightened importance. In the general population, E/C/F/TAF demonstrated similar efficacy and improved bone and renal safety compared with E/C/F/TDF.

**Materials and methods:**

Treatment-naïve HIV-positive adults were randomized 1:1 to a single-tablet regimen of E/C/F/TAF or E/C/F/TDF once daily in two phase 3 double-blind studies. Pre-specified pooled week 48 analyses of efficacy, renal safety and bone mineral density (BMD) in those ≥50 years are described in this analysis.

**Results:**

Of 1733 patients treated, 203 were ≥50 years of age; 89 randomized to E/C/F/TAF and 114 to E/C/F/TDF. Baseline viral load, CD4 counts, renal laboratories and BMD were similar. Virologic success by FDA snapshot algorithm at week 48 was 94.4% for E/C/F/TAF versus 91.2% for E/C/F/TDF, similar to efficacy in the entire study population. Adverse events leading to discontinuation were 1.1% for E/C/F/TAF versus 3.5% for E/C/F/TDF. Mean change in estimated glomerular filtration rate at week 48 was −5.8 mL/min for E/C/F/TAF versus −11.7 mL/min for E/C/F/TDF (*p*=0.010). As shown in Figures 1 and 2, subjects ≥50 years had significantly improved renal tubular profiles with E/C/F/TAF versus E/C/F/TDF. Mean decrease in BMD was significantly less in the E/C/F/TAF versus E/C/F/TDF arm; spine (−1.47% vs. −2.77%, *p*=0.011); hip (−0.31% vs. −2.52%, *p*<0.001).

**Conclusions:**

Among subjects ≥50 years of age, those receiving 48 weeks of E/C/F/TAF had comparable efficacy and improved bone and renal safety compared with those on E/C/F/TDF, as previously demonstrated in the overall study population. These findings demonstrate an important safety improvement of TAF relative to TDF in patients 50 years and older, which is of particular importance as the population living with HIV ages and experiences more non-AIDS-related co-morbidities.

**Abstract P004–Figure 1:** Changes in quantitative proteinuria in subjects ≥50 years.

**Abstract P004–Figure 2:** Changes in tubular proteinuria in subjects ≥50 years.

# Sub article: P005 Causes of mortality and time to death in people living with HIV in Mexico City

**DOI:** 10.7448/IAS.19.2.21033

**Published:** 2016-04-28

**Authors:** Steven Diego-Diaz, Yanink Caro-Vega, Rocío Carrasco-Hernández, Pablo Belaunzarán Zamudio, Samuel Ponce de León-Rosales, Juan Sierra-Madero

**Affiliations:** 1 Centro para la Prevención y Atención Integral del VIH/SIDA del Distrito Federal, Subdirección de Prevención e Información, Mexico City, Mexico; 2 Instituto Nacional de Ciencias Médicas y Nutrición Salvador Zubirán, Infectología, Mexico City, Mexico; 3 Facultad de Medicina, Universidad Nacional Autónoma de México, División de Investigación, Mexico City, Mexico

## Abstract

**Introduction:**

Despite expansion of universal access to antiretroviral therapy (ART) in Mexico, AIDS mortality has remained constant. Recent data show heterogeneity in AIDS mortality by state and gender pointing to health inequities within the country. Here we describe the causes and time to death in HIV-positive subjects in Mexico City.

**Materials and methods:**

We obtained data from death certificates (2012–2014) from Mexico City's Ministry of Health (MoH). We included certificates in which HIV infection was listed. Causes of death were ICD-coded. We obtained all-cause mortality by age groups from the National Institute for Statistics, Geography and Informatics. We evaluated socio-demographic characteristics, causes of death and time to death after HIV diagnosis by gender. We estimated the proportion of deaths from all-deaths by age group, gender and borough. We fit logistic models to determine factors related to early mortality (EM, deaths occurring the first year after diagnosis).

**Results:**

There were 1602 registered deaths in HIV-positive subjects in Mexico City (2012–2014). Sixty-six per cent were early deaths. Women accounted for 27% of deaths, were older at death than men (57 years vs. 41 years), were less frequently single (22% vs. 60%) and were less educated. Twenty-four per cent of women died in the first month after diagnosis in contrast to men (50%). Twenty-six per cent of women died more than five years after the HIV diagnosis, while 12% of men did. Most deaths (74%) among men were early deaths and concentrated in a few boroughs. Boroughs with the highest number of deaths showed a north–south geographic pattern that did not follow the size of the population or its overall mortality. The most common causes of death were AIDS-defining opportunistic infections (OI), respiratory illnesses, liver disease and non-AIDS-related infections. Male gender, younger age (<20 years), single and lack of social insurance were independent risk factors associated with EM.

**Conclusions:**

A large proportion of deaths in HIV-positive individuals occur within the first year of diagnosis, which might be explained by late diagnosis and poor linkage to care. We observed differences in socio-demographic, geographic and clinical profiles across age groups and gender in HIV-positive individuals that died in Mexico City in 2012–2014. Most deaths occur early in young, uninsured, single men in two city areas. More deaths among women occurred due to chronic diseases late after diagnosis. EM accounts for most of the deaths among uninsured people. Addressing late diagnosis and lack of linkage to care could contribute to reduce AIDS-associated deaths in Mexico City even further.

# Sub article: P006 Co-morbidities in a sample of HIV-positive adults in Puerto Rico

**DOI:** 10.7448/IAS.19.2.21034

**Published:** 2016-04-28

**Authors:** Carlos E Rodriguez-Diaz, Jorge Santana, Edda I Santiago-Rodriguez, Gerardo G Jovet-Toledo, Lourdes Irizarry-Gonzalez, Yemile Ron-Suarez, Juan Carlos Orengo, Felipe Arbelaez, Homero Monsanto

**Affiliations:** 1 University of Puerto Rico-Medical Sciences Campus, School of Public Health, Social Sciences Department, San Juan, Puerto Rico; 2 University of Puerto Rico-Medical Sciences Campus, School of Medicine, AIDS Clinical Trial Unit, San Juan, Puerto Rico; 3 University of Puerto Rico-Medical Sciences Campus, School of Public Health, Center for Sociomedical Research and Evaluation, San Juan, Puerto Rico; 4 Merck & Co., Inc., Merck, Carolina, Puerto Rico

## Abstract

**Introduction:**

Puerto Rico (PR) is among the areas with the highest estimated rates of people with HIV in the United States. Despite the availability of epidemiologic data, there is a lack of real-world information that can help in understanding the co-morbidities of people with HIV in PR. The objective of this study was to describe common co-morbidities among HIV-positive adults who attend treatment clinics in PR.

**Methods:**

An exploratory, retrospective, cross-sectional study was conducted at five clinics that provide services to people with HIV in PR. A random sample of medical records was reviewed from HIV-positive adult patients. Patient with HIV-related illness and pregnant women were excluded from the sample. Descriptive statistics were used to summarize patient demographics, morbidity and clinical characteristics. Multivariate analyses were conducted to explore differences by age and sex.

**Results:**

A total of 250 (179 men and 71 women) medical records were reviewed. The mean age was 47.9 years, and in average, they have been living with HIV for nine years. Most (97.6%) had at least one co-morbidity. The most common co-morbidities were dyslipidaemia (men: 60.8%; women: 69.0%) and hypertension (men: 39.6%; women: 46.5%). The most common co-morbidity treatments and supplements were multivitamins, lipid lowering therapy and antihypertensive agents. These remain as the most common treatments after stratifying by sex. Men were more likely to have been diagnosed with alcohol misuse (OR=4.08), while women were more likely to have been diagnosed with obesity (OR=2.95), human papillomavirus (OR=2.80), hypothyroidism (OR=3.46) and osteoporosis (OR=9.68). Younger participants (<50 years) were more likely to have alcohol misuse (OR=2.04), while the older individuals (>50) were more likely to have been diagnosed with dyslipidaemia (OR=3.21), hypertension (OR=2.94) and diabetes mellitus (OR=3.40). Controlling by sex and age, women were more likely to have been diagnosed with obesity (OR=3.20) and depression (OR=1.99). Co-morbidities associated with the >50 age group were dyslipidaemia (OR=3.08), hypertension (OR=2.85), HPV (OR=2.55) and diabetes mellitus (OR=3.33).

**Conclusions:**

This is one the first studies to assess co-morbidities among people with HIV in PR, and one of few among Latino populations within the United States. Consistent with other studies, cardiovascular diseases are common among people with HIV in PR. Findings support the need for awareness and real-world evidence about the necessity of considering the co-morbidities of people with HIV when implementing screening and preventive tests and prescribing drug therapy.

# Sub article: P007 Baseline characteristics of a prospective cohort of adult HIV-positive patients in Latin America (LATINA)

**DOI:** 10.7448/IAS.19.2.21035

**Published:** 2016-04-28

**Authors:** Gabriela Rodriguez Loria, Marcelo Losso, Luis Mosqueda, Guillermo Viloria, Jorge Alave, Jaime Andrade Villanueva, Mariana De Paz, Hector Laplume, Waldo Belloso

**Affiliations:** 1 Fundacion Ibis, Research, Buenos Aires, Argentina; 2 Cical, Research, Buenos Aires, Argentina; 3 Hospital General Ramos Mejia, Immunocompromised Patient, Buenos Aires, Argentina; 4 Hospital de Barranco, Infectology, Lima, Peru; 5 Hospital Civil de Guadalajara, Infectology, Buenos Aires, Argentina; 6 Hospital Italiano de Buenos Aires, Infectology, Buenos Aires, Argentina; 7 Hospital Posadas, Infectology, Buenos Aires, Argentina

## Abstract

**Introduction:**

Though HIV epidemics are not decreasing in most Latin American countries, there is little information about the baseline demographics, clinical status at diagnosis and therapeutic course of patients initiating follow-up. LATINA Cohort has already provided information about these issues in its retrospective component. We present here the first analysis of its prospective component based upon sites from three countries.

**Material and methods:**

The cohort is built by a multinational Executive Committee and National Coordinating Centers in charge of local logistics, identification and inclusion of contributing sites. From initial involvement of sites who participated in the retrospective cohort, we moved forward to include new sites specializing in HIV ensuring data quality. The prospective cohort started gathering data in September 2013 from six sites and progressed to the current 12 distributed in Argentina (nine sites), Mexico (two sites) and Peru (one site). Data presented here correspond to patients enrolled until March 2015. Inclusion criteria were recent HIV diagnosis (within one year) and at least two prior visits to the site in order to ensure commitment. Data collection was performed through a website (www.latina.cical.org) including data obtained in standard of care visit. Diagnosis for AIDS defining diseases was based on CDC criteria, and standardized criteria were developed by the Executive Committee for serious non-AIDS events. Database was periodically checked for incompleteness and consistency. All data were consolidated in an Access database and analyzed using Statistics V17.

**Results:**

A total of 748 patients were included in this analysis of baseline visit; 578 (77%) were from Mexican sites, 158 (21%) from Argentinian and 12 (2%) from Peruvian Site. Characteristics of our cohort were provided in Table 1.

**Conclusions:**

Data presented reflects the situation of HIV epidemics, with mostly young males, predominantly men who have sex with men and with a significant proportion of late presenters and self-reported users of alcohol and tobacco. A major limitation of this cohort is selection bias due to convenience sampling. Data quality is acceptable among all participating sites present and constitutes a quite diverse and interesting data set for both continue to follow-up and expansion of its regional representativity.

**Abstract P007–Table 1:** Baseline characteristics (N=752)

Characteristics (N=752)	Median	IQR
**Age (years)**	34	27 to 42
**Education (years)**	11.1	8 to 15
**CD4 count (cells/mL)**	443	218 to 614
**Viral load (copies/mL)**	32509	2590 to 138000
	**Mean**	**SD**

**Weight (kg)**	67.4	14.84
**Height (cm)**	168.72	21
**Blood pressure (mm Hg)**		
-Systolic blood pressure	111.31	13.51
-Diastolic blood pressure	70.46	9.9
	**Percentage**	**95% CI**

**Male at birth**	86	84 to 89
**White race**	41	37 to 44
**Health coverage**		
-Public	88.1	85.8 to 90.4
-Private	6.8	5.0 to 8.6
-Social security	5.1	3.5 to 6.7
**Mode of transmission**		
-MSM/bisexual	60.6	57.11 to 11.09
-Heterosexual	33.8	30.42 to 37.18
-Missing data	3.9	2.5 to 5.28
-Parenteral	0.7	0.1 to 1.3
-Unknown	0.9	0.23 to 1.57
-Vertical	0.1	0 to 0.33
**Symptoms at diagnosis (N=276)**		
-Weight loss	14.6	10.43 to 18.77
-Fever >2 weeks	8.4	5.13 to 11.67
-Chronic diarrhoea	6.5	3.59 to 9.41
-Lymphadenopathies	2.9	0.92 to 4.88
-Herpes	2.7	0.79 to 4.16
-Oral candidiasis	1.5	0.07 to 2.93
-Oral leucoplakia	0.1	0 to 0.47
**Patients showing progression of disease at baseline**	23.8	20.76 to 26.84
**Other conditions**	18.1	15.35 to 20.85
**Current habits (self-reported)**		
Smoking	45	39.13 to 50.87
Drugs	8.6	5.29 to 11.91
Alcohol consumption	28.3	22.99 to 33.61

Age was significantly lower in Mexican population (34.71 vs. 38.27, mean difference 3.5795%CI: −5.46 to 1.66) as well as systolic blood pressure and diastolic blood pressure (108.32 vs. 120.58, *p*=0.04 and 68.87 vs. 75.43, *p*=0.01). The most frequent non-HIV-related conditions found were sexually transmitted disease (N=44) and hypertension (N=33). Co-infection with HCV was found in 11 patients (1.1%; 0.07 to 2.93). HBV was found in 14 patients (1.9%; 0.29 to 3.51).

# Sub article: P008 Ledipasvir/sofosbuvir is safe and effective for the treatment of patients with genotype 1 chronic HCV infection in both HCV mono-and HCV/HIV co-infected patients

**DOI:** 10.7448/IAS.19.2.21036

**Published:** 2016-04-28

**Authors:** Jorge Santana, Mark Sulkowski, Curtis Cooper, Paul Kwo, Kris Kowdley, Sarah E Kleinstein, Nelson Cheinquer, Sarjita Naik, John Wolf, Macky Natha, Polina German, Luisa Stamm, Diana Brainard, Susanna Naggie

**Affiliations:** 1 University of Puerto Rico, School of Medicine, San Juan, Puerto Rico; 2 Johns Hopkins University, School of Medicine, Baltimore, USA; 3 The Ottawa Hospital, Ottawa Hospital and Regional Hepatitis Program, Ottawa, Canada; 4 Indiana University, Department of Medicine, Indianapolis, USA; 5 Swedish Medical Center, Liver Care Network, and Organ Care Research, Seattle, USA; 6 Columbia University, Institute for Genomic Medicine, New York, USA; 7 Public Health and Medical Affairs, Gilead Sciences, Sao Paulo, Brazil; 8 Medical Affairs, Gilead Sciences, Foster City, CA, USA; 9 Clinical Pharmacology, Gilead Sciences, Foster City, CA, USA; 10 Clinical Research, Gilead Sciences, Foster City, CA, USA; 11 Department of Medicine, Duke University, Durham, USA

## Abstract

**Introduction:**

Current AASLD/IDSA Hepatitis C Guidance states that HCV/HIV-co-infected persons should be treated the same as persons without HIV infection, after recognizing and managing interactions with antiretrovirals (ARVs). We compared the safety and efficacy of the single-tablet regimen of ledipasvir/sofosbuvir (LDV/SOF) in HCV genotype (GT) 1 patients co-infected with HIV-1 in the Phase III ION-4 study with HCV monoinfected GT1 patients in the Phase III ION 1–3 studies.

**Methods:**

In the ION-4 study, 327 GT1 HCV/HIV-co-infected patients received LDV/SOF 90/400 mg daily for 12 weeks. In the ION 1–3 studies, 538 GT1 HCV monoinfected patients received LDV/SOF 90/400 mg daily for 12 weeks. This pooled analysis will assess safety and sustained virologic response at week 12 (SVR12). LDV, SOF and GS-331007 exposures in HCV/HIV-co-infected subjects were also compared across ARV regimens, race, SVR12 and to subjects with HCV monoinfection. A genome-wide association study (GWAS) was conducted to identify host genetic determinants of HCV relapse after LDV/SOF therapy in HCV/HIV-co-infected individuals on ARVs.

**Results:**

A total of 865 patients were treated with LDV/SOF for 12 weeks in the Phase III ION programme. In ION-4, the overall SVR12 was 96% and relapse rate was 3%. In ION 1–3, the overall SVR12 was 97% and the relapse rate was 2%. SVR12 by treatment history, cirrhosis status, race and GT1 subtype is reported in Table 1. Most common adverse events (>10% reported in any arm) were fatigue, headache, diarrhoea and nausea. There were no clinically relevant differences in pharmacokinetics (PK) of LDV/SOF in HCV/HIV-co-infected subjects compared with HCV monoinfected subjects. GWAS did not reveal significant associations with HCV relapse.

**Conclusions:**

In this pooled analysis, the once daily, single-tablet regimen of LDV/SOF for 12 weeks provided high rates of SVR regardless of the presence of HIV infection and is a safe, well-tolerated option for patients with both HCV monoinfection and HCV/HIV co-infection.

**Abstract P008–Table 1:** SVR12 by treatment history, cirrhosis status, race and GT1 subtype

Study	ION 1	ION 2	ION 3	ION 1–3 (combined)	ION 4 (HIV)
Treatment-naïve SVR12 (%)	210/213 (99)		208/216 (96)	418/429 (97)	138/146 (95)
Treatment-experienced SVR12 (%)		102/109 (94)		102/109 (94)	175/181 (97)
Cirrhosis SVR12 (%)	32/34 (94)	19/22 (86)		51/56 (92)	63/67 (94)
Non-cirrhotic SVR12 (%)	179/179 (100)	83/87 (95)	208/216 (96)	470/482 (98)	250/260 (96)
Blacks SVR12 (%)	24/24 (100)	24/24 (100)	40/42 (95)	89/90 (99)	103/115 (90)
Non-blacks SVR12 (%)	188/190 (99)	78/85 (92)	165/173 (95)	431/448 (96)	215/217 (99)
GT 1a	142/145 (98)	82/86 (95)	165/172 (96)	389/403 (97)	239/250 (96)
GT 1b	67/67 (100)	20/23 (87)	43/44 (98)	130/134 (97)	74/77 (96)

# Sub article: P009 Ledipasvir/sofosbuvir for 12 weeks in patients co-infected with HCV and HIV-1

**DOI:** 10.7448/IAS.19.2.21037

**Published:** 2016-04-28

**Authors:** Michael Saag, Jorge Santana, Mark Sulkowski, Curtis Cooper, Douglas Dieterich, Sarah E Kleinstein, Nelson Cheinquer, Sarjita Naik, John Wolf, Macky Natha, Polina German, Luisa Stamm, Diana Brainard, Susanna Naggie

**Affiliations:** 1 Division of Infectious Diseases, University of Alabama, Birmingham, USA; 2 University of Puerto Rico, School of Medicine, San Juan, Puerto Rico; 3 Johns Hopkins University, School of Medicine, Baltimore, USA; 4 Division of Infectious Diseases, University of Ottawa, Ottawa, Canada; 5 Department of Medicine, The Mount Sinai Hospital, New York, USA; 6 Columbia University, Institute for Genomic Medicine, New York, USA; 7 Public Health and Medical Affairs, Gilead Sciences, Sao Paulo, Brazil; 8 Medical Affairs, Gilead Sciences, Foster City, CA, USA; 9 Clinical Pharmacology, Gilead Sciences, Foster City, CA, USA; 10 Clinical Research, Gilead Sciences, Foster City, CA, USA; 11 Department of Medicine, Duke University School of Medicine, Durham, USA

## Abstract

**Introduction:**

Historically, HIV co-infection was considered a negative predictor of HCV response to treatment with interferon/ribavirin (IFN/RBV). For sofosbuvir-based regimens, HIV/HCV patients have achieved similar sustained virologic response (SVR) rates as HCV monoinfected patients. We evaluated the safety and efficacy of the IFN-free, RBV-free, single-tablet regimen of ledipasvir/sofosbuvir (LDV/SOF) in HCV genotype 1 or 4 patients co-infected with HIV-1 in the Phase 3 ION-4 study. Co-infection is no longer considered a special population, and HIV/HCV-co-infected persons should be treated the same as persons without HIV infection.

**Materials and methods:**

HCV treatment-naïve and experienced HIV co-infected patients on stable, antiretroviral (ARV) regimens were enrolled and received LDV/SOF (90 mg/400 mg) once daily for 12 weeks. Permitted concomitant ARVs included tenofovir and emtricitabine (TDF+FTC) with raltegravir (RAL), efavirenz (EFV) or rilpivirine (RPV). Safety evaluations included adverse event (AE) and laboratory monitoring including renal parameters, CD4 count and HIV-1 RNA levels. The primary efficacy endpoint was SVR12. The pharmacokinetics (PK) of LDV, SOF, GS-331007 (SOF predominant circulating metabolite) and tenofovir (TFV) were evaluated in all subjects with measurable plasma concentrations using previously established population PK models. A genome-wide association study (GWAS) was conducted to identify common host genetic determinants of HCV relapse after LDV/SOF therapy in HIV/HCV individuals on antiretrovirals (ARVs).

**Results:**

In the ION-4 study, 335 patients with GT1a (75%), GT1b (23%) and GT4 (2%) were enrolled; 82% were male, 61% were white, mean age was 52 (range 26–72), mean baseline HCV RNA was 6.7 log10 IU/mL (range 4.1–7.8), median baseline CD4 count was 662 cells/µL (Q1, Q3=469, 823), 20% had cirrhosis, 24% were IL28B CC genotype and 55% had not responded to prior HCV treatment. Overall, the SVR12 rate was 96% (322/335); two patients had on-treatment virologic failure likely due to non-compliance and 10 had virologic relapse after discontinuing treatment. SVR12 was similar among non-cirrhotic (96%) and cirrhotic (94%) patients and also among treatment-naïve (94%) and treatment-experienced (97%) patients. SVR12 by ARV regimen is shown (Table 1). AEs occurring in ≥10% of patients were headache (25%), fatigue (21%) and diarrhoea (11%). Exposures of LDV, SOF and GS-331007 were comparable across ARV regimens, race and treatment outcome. GWAS did not reveal significant associations with HCV relapse.

**Conclusions:**

The IFN-free, RBV-free, single-tablet regimen of LDV/SOF administered once daily for 12 weeks is highly effective and well tolerated in treatment-naïve and experienced, genotype 1 or 4 HCV-infected patients with HIV-1.

**Abstract P009–Table 1:** Efficacy and reasons for not achieving SVR12 by ARV regimen

Virologic Response	TDF+FTC+EFV (*n*=160)	TDF+FTC+RAL (*n*=146)	TDF+FTC+RPV (*n*=29)	Overall (*n*=335)
SVR12, *n* (%)	151 (94)	141 (97)	28 (97)	320 (96)
On-Treatment Failure, *n* (%)	1 (<1)	0	1 (3)	2 (<1)
Relapse, *n* (%)	8 (5)	2 (1)	0	10 (3)
Other, *n* (%)	0	1 (<2)	0	2 (<1)

# Sub article: P010 Improved safety and efficacy of TAF versus TDF single-tablet regimen in HIV-1 treatment-naïve women through week 48

**DOI:** 10.7448/IAS.19.2.21038

**Published:** 2016-04-28

**Authors:** Ellen Koenig, Chloe Orkin, Amanda Clarke, Daniel Podzamczer, Bonaventura Clotet, Christian Callebaut, Devi Sengupta, Moupali Das, Anita Silva, Marshall Fordyce

**Affiliations:** 1 Infectious Diseases, Instituto Dominicano de Estudos Virologicos, Santo Domingo, Dominican Republic; 2 Royal London Hospital, HIV and HIV/hepatitis C Research Unit, London, UK; 3 Royal Sussex County Hospital, NA, Brighton, UK; 4 Hospital Universitari de Bellvitge-IDIBELL, NA, Barcelona, Spain; 5 IrsiCaixa, Hospital Universitari Germans Trias i Pujol, NA, Barcelona, Spain; 6 Clinical Virology, Gilead Sciences, Foster City, CA, USA; 7 Gilead Sciences, Clinical Research, Foster City, CA, USA; 8 Gilead Sciences, Public Health & Medical Affairs, São Paulo, Brazil

## Abstract

**Introduction:**

Women represent half of all HIV-infected individuals globally, but current treatment practices are based largely on data from male populations. The objective of this analysis was to determine whether there was a difference in safety or efficacy between a tenofovir alafenamide (TAF) -based and a tenofovir diphosphate (TDF)-based regimens in treatment-naïve women.

**Materials and methods:**

Treatment-naïve HIV-1-infected adults with eGFR ≥50 mL/min were randomized 1:1 to a single-tablet regimen of elvitegravir/cobicistat/emtricitabine/TAF (E/C/F/TAF) or elvitegravir/cobicistat/emtricitabine/TDF (E/C/F/TDF) in two controlled, double-blind, randomized phase 3 studies. A pre-specified analysis of efficacy and a post hoc analysis of safety of TAF versus TDF at week 48 by gender were conducted.

**Results:**

Of the 1733 subjects treated, 260 (15%) were women. Baseline HIV viral load and CD4 count were balanced between women randomized to receive either TAF or TDF. At week 48, rates of virologic success (defined as HIV viral load <50 copies/mL) were higher for women receiving TAF versus TDF [95% vs. 87%; 95% CI (0.2,15.6); *p*=0.023]. Both regimens were well-tolerated, and proximal renal tubulopathy was not observed. The percentages of subjects experiencing treatment-emergent grade 3 or 4 adverse events (AEs) were comparable in men and women (TAF: men 8%, women 9%; TDF: men 8.1%, women 11.8%). Individual AEs were generally similar in men and women for both treatment groups. Among women, decline in median eGFR was significantly less (*p*<0.001) in the TAF group, while median changes in proteinuria and albuminuria were comparable between treatment arms. Women receiving TAF experienced significantly less spine, and hip bone mineral density (BMD) declines compared with those receiving TDF (*p*<0.001).

**Conclusions:**

In treatment-naïve women, E/C/F/TAF demonstrates higher rates of virologic success and significant improvements in renal and bone safety compared to E/C/F/TDF at week 48. These results support the use of E/C/F/TAF for the initial treatment of HIV-1 in women.

**Abstract P010–Table 1:** TAF versus TDF single-tablet-regimen in HIV-1-infected women

	TAF (*n*=133)	TDF (*n*=127)	*p*-value
Median baseline HIV viral load (log10 copies/mL)	4.50	4.46	0.70
Median baseline CD4 (/µL)	358	367	0.75
Virologic success at W48, %	95%	87%	0.023
Median change in CD4 (/µL) at W48	216	181	0.37
Discontinuation due to treatment-emergent adverse events (AEs), %	0	1.6	0.24
Treatment-emergent grade 3 or 4 AEs, %	9	11.8	0.54
PRT (proximal renal tubulopathy), *n*	0	0	–
Median changes in eGFR (mL/min), W48	−5.4	−12.6	<0.001
Median % changes in UPCR (urine protein/creatinine mg/g), W48	−9.7	−1.2	0.31
Median % changes in UACR (urine albumin/creatinine mg/g), W48	−4.1	+8.3	0.40
Mean % changes in spine BMD, W48	−0.959	−2.912	<0.001
Mean % changes in hip BMD, W48	−1.255	−3.475	<0.001

# Sub article: P011 Pregnancies in adolescents infected with HIV in the state of São Paulo, Brazil: challenges to avoid mother-to-child transmission of HIV and keep these adolescents alive

**DOI:** 10.7448/IAS.19.2.21039

**Published:** 2016-04-28

**Authors:** Maria Aparecida Silva, Carmen Silvia B Domingues, Angela Tayra

**Affiliations:** São Paulo State Program for STDs and AIDS, STD and AIDS Referral and Training Center, São Paulo State Department of Health, São Paulo, Brazil

## Abstract

**Introduction:**

Adolescence is the transition between childhood and adulthood. Primary care health units concentrate visits in antenatal care (AN), child health and chronic diseases, which may prevent adolescents from using these services. Adolescents may only access services when pregnant or with symptoms of a disease, including sexually transmitted diseases (STDs). The aim of this study was to describe AN, time of HIV diagnosis and pregnancy outcome in HIV-positive adolescents in the state of São Paulo (SSP) between 1999 and 2015.

**Materials and methods:**

Descriptive study of 12 to 19 year-old HIV-positive pregnant teenagers, living in SSP, diagnosed with HIV between 1 January 1999 and 30 June 2015. Data source: cases reported to the Disease Information System (Sinan).

**Results:**

1793 pregnancies were reported in HIV-positive adolescents, about 8% (1793/21,662) of total of the SSP. Pregnancies occurred in 1591 adolescents: 88.3% (1405/1591) had one pregnancy, 10.7% (171/1591) two, and 0.9% (15/1591) three or more. In 43.9% (788/1793) of pregnancies, HIV diagnosis was before AN, 44.3% (795/1793) during AN and 9.0% (162/1793) during or after childbirth; AN occurred in 91.2% (1636/1793) of pregnancies, with 56.8% (929/1636) starting in the 2nd or 3rd trimester; in 74.5% (1335/1793) antiretrovirals were used during AN, in 7.3% (131/1,793) not used, and in 18.2% (327/1793) not specified; 56.0% (1004/1793) evolved to caesarean delivery and 26.9% (482/1793) to vaginal delivery; miscarriages and stillbirths accounted for 2.3% (41/1793). Excluding miscarriages, stillbirths and ongoing pregnancies, in 73.3% (1263/1722) intravenous zidovudine was used during childbirth, in 9.9% (171/1722) not used, and in 16.7% (288/1722) not specified.

**Conclusions:**

In this population, the high proportion of pregnancies diagnosed with HIV before AN suggest adolescents are infected by perinatal transmission or with unprotected early sexual activity. The care network must discuss specific strategies of access to services for adolescents, prevention inputs, sexual and reproductive healthcare, including access to long-acting contraceptives. It is important to link and retain this population in HIV services, to improve coverage and adherence to antiretrovirals, reduce viral load and maintain virologic suppression, prevent mother-to-child transmission of HIV and keep these adolescents alive.

# Sub article: P012 Case investigation protocol and committee as strategies to reduce and eliminate mother-to-child transmission of HIV: experience of the state of São Paulo, Brazil

**DOI:** 10.7448/IAS.19.2.21040

**Published:** 2016-04-28

**Authors:** Carmen Silvia B Domingues, Maria Aparecida Silva, Angela Tayra, Maria Clara Gianna, Surveillance of Maternal and Infant Mortality

**Affiliations:** 1 Sao Paulo State Program for STDs and AIDS, STD and AIDS Referral and Training Center, Sao Paulo State Department of Health, Sao Paulo, Brazil; 2 Department of Health, Sao Paulo State Department of Health, Sao Paulo, Brazil

## Abstract

**Introduction:**

Although intervention measures to prevent mother-to-child transmission (MTCT) of HIV are available in healthcare services for pregnant women, post-partum women and children, several social, political, economic and individual factors may hinder access of this population to these measures, contributing to the occurrence of cases. In 2014, the National STD/AIDS Department recommended the implementation of state and municipal committees to investigate, discuss and propose measures to reduce and eliminate MTCT of HIV, using a pre-established protocol to identify determinants of the disease. The committees are intra-institutional, inter-institutional, and multidisciplinary bodies with confidential technical performance and educational function, essential for monitoring and evaluating healthcare policies. The state of Sao Paulo (SSP) proposed using Mother and Child Mortality Committees, given they have already been established and are operating regularly. MTCT has declined in SSP – 538 cases in 1996 and 21 in 2013, by year of birth. The aim of this study was to describe MTCT research results in children under 5 years of age, born in the SSP between 2007 and 2013.

**Materials and methods:**

Descriptive study using research protocol with 67 questions in 3 groups: antenatal, delivery and postpartum/child monitoring [1]. At the conclusion of the protocol, determinants are classified into types of vulnerability: individual-social maternal and program (service or management) for decision making.

**Results:**

The 90 protocols studied 53 cases (58.9%) by individual-social maternal vulnerability, 8 (8.9%) programme-services, 17 (18.9%) in both areas, 9 (10%) under investigation, and 3 (3.3%) despite infection, followed prophylactic measures correctly. Main categories of individual and social vulnerability: 20 cases of drug use (“crack,” alcohol and others), 7 living in the streets and drug use, 17 with low-income and 10 immigrants; for program-service: gaps in prevention measures, search for absences, and improving adherence to antiretroviral drugs. Median maternal age of 30 years, 37.8% were single and 41% housewives.

**Conclusions:**

The success of the national HIV/AIDS policy has reduced MTCT. Currently, determinants of this disease are mainly related to individual and social components of this population and their accessibility to services. Committees can contribute to improve healthcare activities and quality of public policies to overcome these barriers.

## References

[1] http://www.aids.gov.br/sites/default/files/anexos/publicacao/2014/56592/tv_2_pdf_18693.pdf.

# Sub article: P013 Long-term follow-up of HIV-infected mothers from perinatal and LILAC studies in Buenos Aires, Argentina

**DOI:** 10.7448/IAS.19.2.21041

**Published:** 2016-04-28

**Authors:** Danielle Gladstone, Silvina Ivalo, Sharon Nachman, Marcelo Losso

**Affiliations:** 1 Stony Brook University Hospital, Pediatric Infectious Disease, Stony Brook, New York, USA; 2 Hospital J. M. Ramos Mejia, Immunocompromised, Buenos Aires, Argentina

## Abstract

**Introduction:**

HIV treatment guidelines updates following the results of the Strategic Timing of AntiRetroviral Treatment (START) study and WHO B+ guidelines have changed antiretroviral (ARV) treatment practices for HIV-infected post-partum women. Factors significant to predicting treatment abandonment (ABND) among these patients differ globally. Our study aim was to evaluate the factors that are associated with ABND in this cohort.

**Methods:**

Retrospective review of charts from all women that participated in either or both the BA perinatal study (2002–2007) and the BA LILAC: The longitudinal study in Latin American countries (2008–2012) was included. Data collected included patient status at the study completion visit through 31 July 2015, medical indication for ARV treatment, ABND, alcohol (ETOH), smoking or substance use, CD4+ nadir and most recent count, hospitalizations, hepatitis co-infection (B/C), opportunistic infections (OIs), change in CDC status, sex industry employment and year last seen in clinic. Associations with the defined outcome were estimated using chi-square analysis.

**Results:**

Of the 150 women (178 pregnancies), charts were available on 108 women. Only 72 out of 150 were still in care through 2015. Of the 51 women recommended to continue ARV post-perinatal/LILAC; age 32, (range 26.2–43.7 years, SD 3.75), 27% had B/C, 6% were sex workers, 27% were drug abusers, 13% ETOH, 33% smoked cigarettes and 29% were AIDS defined (41% CDC A). Thirty-seven (72%) abandoned ARV treatment at some point with 40% of those women developing an OI while off-treatment; 57 women did not have an end of study indication for ARVs. On average, they were 27.6 years of age (range 18.49–37.38, SD 4.96); 12% had B/C and 5% were sex workers; 23% were drug abusers, 9% ETOH and 36% smoked cigarettes. At the time of completion of their prior study, 94% were CDC A. Forty-one were later recommended to start therapy, of which 71% abandoned therapy. 46% of those recommended to start therapy (post-perinatal/LILAC) but who ABND, developed an OI. Maternal ARV ABND was not predicted by smoking status, ETOH/substance use, hepatitis B/C status, HIV status of infant, or prior parity. Being a sex worker (*p*=0.03) and CDC C (*p*<0.001) were associated with predicting ARV ABND.

**Conclusions:**

In this long-term follow-up study, many women continued in intermittent care despite not taking ARVs; 71–72% of these women stopped ARV treatment at least once during the follow-up period despite having an indication to continue treatment. Barriers to retention in care required further evaluation.

# Sub article: P014 Implementation gaps for interventions to prevent mother-to-child transmission of HIV in Mexico during 2014

**DOI:** 10.7448/IAS.19.2.21042

**Published:** 2016-04-28

**Authors:** Adriana Villafuerte Garcia, María del Pilar Rivera Reyes, Patricia Uribe Zúñiga, Carlos Magis Rodríguez

**Affiliations:** 1 Centro Nacional para la Prevención y el Control del VIH y el sida, Atención Integral, Mexico City, Mexico; 2 Centro Nacional para la Prevención y el Control del VIH y el sida, Investigación Operativa, Mexico City, Mexico; 3 Centro Nacional para la Prevención y el Control del VIH y el sida, Dirección General, Mexico City, Mexico

## Abstract

**Background:**

Since the commitment to eliminate the mother-to-child transmission (MTCT) [1,2] of HIV by 2018, the Mexican Health Ministry has generated evidence that contributes to the design of strategies for HIV testing promotion in pregnant women and their linkage to HIV health services. As part of these actions, the current study gives an overview of the gap between the detection and the antiretroviral treatment (ART) access to prevent the MTCT (PMTCT) of the HIV; also, it shows the need to strengthen the monitoring of cases considering the moment of transmission and the timeliness of diagnosis.

**Methods:**

We estimated the number of pregnant women with HIV in 2014 through the UNAIDS Spectrum model. The total detections were obtained from the percentage of screening coverage [3]. The number of pregnant women with HIV who received ARV for PMTCT was obtained from the reports of the HIV Sector Information Group (part of the National Council for the Prevention and Control of AIDS). The perinatal cases from the National Register Cases of AIDS (NRC) were analyzed. We classified the cases according to the year of birth and the diagnosis age. Finally, an estimate of the expected number of cases was performed.

**Results:**

From an estimation of 1,450 pregnant women with HIV in 2014, 59% (*n*=856) were detected and 38% (*n*=555) received ARV for PMTCT. Likewise, based on the NRC, between 2014 and 2015, 36 children that born in 2014 were diagnosed with vertical HIV transmission. The cases of vertical HIV transmission review from 2004 to 2014 show that on average 32% of these cases were timely diagnosed. The trend was similar in the past 10 years (between 21% and 39%), except in 2014, where there was an increase compared to 2013 (48% vs. 25%). The analysis of the cases according to the birth year showed that the decrease observed, based on the number of diagnostics reported annually, is a reflection of the delay diagnoses but not a reduction in transmission because according to the estimation, 113 cases are finally expected in 2014.

**Conclusions:**

In Mexico, the low coverage of detection and connection to the continuum of care results in a low proportion of pregnant women having access to ART. The Pan American Health Organization recommended surveillance based on case reports to facilitate longitudinal tracking of infected people [4]; however, the gaps make the surveillance partially retrospective, limiting, for now, the possibility of preventing the transmission.

## References

[1] Pan American Health Organization, United Nations Children's Fund (2010). Regional initiative for the elimination of mother-to-child transmission of HIV and congenital syphilis in Latin America and the Caribbean.

[2] Mexican Health Ministry (2013). Specific Action Program in response to HIV, AIDS and STIs 2013–2018.

[3] National Council for the Prevention and Control of AIDS, Monitoring and Evaluation Committee, Sector Information Group, Bulletin No. 12, consulted on: http://www.censida.salud.gob.mx/descargas/comites/monitoreo/Bol12_2014_21122015_VER_S_T.pdf.

[4] Pan American Health Organization, World Health Organization (2014). Field guide for implementation of the strategy and plan of action for elimination of mother-to-child transmission of HIV and congenital syphilis in the Americas.

# Sub article: P015 Antiretroviral resistance among pregnant women in São Paulo, Brazil

**DOI:** 10.7448/IAS.19.2.21046

**Published:** 2016-04-28

**Authors:** Giselle Ibette Silva López Lopes, Gabriela Bastos Cabral, Jaqueline de Souza Cavalcanti, Geovana Rafaela Silva, Norberto Camilo Campos, Camila Rodrigues, Elaine Monterio Matsuda, Luis Fernando de Macedo Brígido

**Affiliations:** 1 Adolfo Lutz Institute, Virology, São Paulo, Brazil; 2 NAIC, Clinical Medicine, Carapicuiba, Brazil; 3 ARMI Santo Andre, Clinical Medicine, Santo Andre, Brazil

## Abstract

**Introduction:**

Pregnant women living with HIV, either diagnosed during prenatal care or on regular follow-up, may benefit from viral suppression, also essential for reduction of vertical transmission. The aim of the study was to access ARV resistance during pregnancy and the immunological and virological outcome at the time of delivery.

**Methods:**

The study included 86 pregnant women living with HIV, genotyped from 2013 to 2015. Resistance profile was compared to that of 145 women at reproductive age failing ARV in that period. Partial pol was obtained from RNA (nested RT-PCR followed by big dye, Life) analyzed at Stanford-HIVdb. CD4 cell counts (flow cytometry, BD, San Jose, CA, USA), viremia (RealTime, Abbott, Abbott Park, Illinois, USA) and clinical information obtained from electronic database or medical records. Epi Info6 and Prisma (Mann–Whitney or Yates corrected) were used for data storage and analysis. Results expressed as medians or proportions.

**Results:**

At genotyping, these pregnant women were at a 22 gestational weeks, age 27 years and older, exposed to two ART regimens, viremia 3.39 Log10 and CD4 384 cells/mm^3^. Only 74% (64/86) had treatment information, 34% (22/64) naïve (age 26 years and older, CD4 354 cells/mm^3^, viraemia 3.73 Log10). Transmitted resistance was observed in 4.5% (1/22) of naïve cases. At delivery, 50% were aviraemic, and overall viraemia had significantly lowered to 1.82 (*p*=0.003). Comparing treated pregnant women to other women failing ARV, they tended to be younger (28 vs. 38 years and older, *p*<0.0001), less exposed to ART (2 vs. 3 regimens, *p*=0.003), had a lower viraemia (3.20 vs. 4.43 Log10, *p*<0.0001) and a higher CD4 (403 vs. 233 cells/mm^3^, *p*=0.0002). Most pregnant women on treatment showed no resistance mutations (65% vs. 25%, *p*<0.0001). At delivery, 46% were with undetectable viral load and viraemia was also significantly lowered to 1.83 Log10 (*p*<0.0001).

**Conclusions:**

Resistant test may have helped to guide ART use during pregnancy, improving viral suppression. Levels of transmitted resistance are low but the limited number evaluated warrants testing of all pregnant women, especially when tested only near delivery.

# Sub article: P016 HIV testing, prenatal care and diagnosis in pregnant women hospitalized in a public hospital in Buenos Aires

**DOI:** 10.7448/IAS.19.2.21043

**Published:** 2016-04-28

**Authors:** Silvina Ivalo, Manuela Bulló, Alejandro Hakim, Claudia Scalise, Marcelo Losso, Sofie Terwel

**Affiliations:** 1 Hospital General de Agudos J. M. Ramos Mejía, Inmunocomprometidos, Buenos Aires, Argentina; 2 Hospital General de Agudos J. M. Ramos Mejía, Obstetrics, Buenos Aires, Argentina; 3 Wageningen University, Biology, Wageningen, Netherlands

## Abstract

**Introduction:**

Prenatal care (PC) and HIV testing in pregnant women have a high impact on prevention of mother-to-child transmission (MTCT) of HIV. Hospital J. M. Ramos Mejía is a public institution where most of the well-known strategies to prevent MTCT are available; nonetheless there have been 15 cases of HIV vertically infected children between 2003 and 2015. The primary aim of this study was to evaluate conditions of PC and HIV testing in pregnant women hospitalized in the maternity ward of this institution. Outcomes included: percentage of women with term pregnancies (TP); an HIV test done and results available; adequate PC (≥5 visits); knowledge of their partner's HIV status; and receiving HIV counselling (pre- or post-test). In addition, the number of prenatal visits and results of HIV testing as reported was compared with the information available in the clinical histories. Also, the results of this study were compared with a similar study performed in 2007.

**Methodology:**

During an 8-week period between April and June 2015, all pregnant women hospitalized in the maternity ward were invited to participate in a structured interview regarding socio-demographic data, obstetrical information, PC and HIV testing. For qualitative variables frequency values (absolute/relative) were calculated, and for quantitative variables, mean (SD) or median (25th and 75th percentile) was calculated.

**Results:**

Two hundred and nine women participated, three were HIV+ and two of them were diagnosed during their pregnancy. Of the 146 (70%) women with TP, only 77% reported to have done an HIV test and to know the test result; 51% did not know their partner's HIV status; 17% with TP had inadequate PC. This group was more likely to have attained lower education and not to know their own and/or their partner's HIV status; 59% reported not to have received either pre-or post-test counselling. For only 58% of the women, HIV test results were available in their medical histories. No improvement was seen in the provision of PC and HIV testing and counselling in comparison with 2007 findings.

**Conclusions:**

The rate of MTCT in the Hospital J.M. Ramos Mejía is above what is potentially achievable. The results of the present study highlight the need to explore better understand the causes of this performance and the opportunities to improve the PC and the quality of HIV testing and counselling in order to achieve better results in reducing vertical transmission of HIV.

# Sub article: P017 HIV/STI testing among MARPs in Mexico City: a key first step to improve the cascade of care

**DOI:** 10.7448/IAS.19.2.21047

**Published:** 2016-04-28

**Authors:** Veronica Ruiz, Yazmin Medina, Patricia Iracheta, Israel Macias, Luis Juarez, Eduardo Rodriguez, Ricardo Niño, Steven Díaz, Andrea Gonzalez

**Affiliations:** 1 Condesa Specialized Clinic, Mexico City Public Health Services, Mexico, Mexico; 2 Center for HIV Preventio and Comprehensive Care, Mexico City Ministry of Health, Mexico, Mexico; 3 Center for HIV Prevention and Comprehensive Care, Mexico City Ministry of Health, Mexico, Mexico

## Abstract

**Introduction:**

Timely HIV diagnosis is key to controlling the spread of the epidemic among “Most at Risk Population” groups (MARPs) in Mexico City. Given that the HIV epidemic is concentrated in men who have sex with men, transgender women and male sex workers, groups that traditionally do not seek care at public health facilities, it is imperative to implement HIV diagnosis strategies that not only detect positive cases but also retain them for care.

**Materials and methods:**

The HIV diagnosis strategy implemented by the Condesa Specialized Clinics (CEC) laboratory includes a comprehensive approach to detection of not only HIV but also of other sexually transmitted infections (STIs) such as syphilis, hepatitis B and C, CD4 count and viral load. Rapid testing is applied to HIV and syphilis detection and basal CD4 count, while all the other results are available in 5 days. All blood samples are tested using HIV antigen testing (P24) to identify all recent HIV infections. The counselling service is staffed by personnel that are sensitive, and some belong, to the MARP groups. Prevalences of HIV and STIs were estimated, and risks of HIV infection and late diagnosis were calculated.

**Results:**

In 2015 from January to October, over 2,300 persons per month requested an HIV test in the Counselling and Testing service. Of the over 27,000 persons that got tested, 12.7% received a positive HIV test result – 18.7% of men, 3.3% of women and 22.6% of transgender women tested positive for HIV. This is a 915% increase in HIV testing since 2008 (Figure 1).

There has been an important rise in the diagnosis of persons during the acute stage of the infection (recent infections). Overall most people were diagnosed in a timely manner or were diagnosed early. Nevertheless those detected at an advanced stage of the infection (<200 CD4 cells) comprised 34.8% of all new detections. The prevalences of the different groups detected at CEC show the stark difference of vulnerability and risks of HIV infections. (Table 1)

**Conclusions:**

An important increase in HIV testing among MARPs can be achieved if the diagnosis process is patient-centred, results are provided quickly and the service is sensitive to the needs of the population. Further work needs to be done in order to reduce the proportion of HIV patients diagnosed in the later stages of infection.

**Abstract P017–Figure 1:** Yearly HIV testing at Condesa Specialized Clinic.

**Abstract P017–Table 1:** Rates of HIV among MARPs diagnosed at Condesa Specialized Clinic

Population Group	HIV Prevalence (%)	Rate HIV+/# of tests
Transgender Women *n*=1.300	41	1 in 2.5
Male Sex Workers *n*=627	37	1 in 2.5
MSM *n*=15.547	19	1 in 5
Women partners of HIV positive men* *n*= 7,110	3	1 in 33
Male Prison Inmates* *n*=33.000	1	1 in 100
Female Prison Inmates* *n*=3,000	1.10	1 in 91
Pregnant Women- D.F. Subway Clinics *n*=6,096 Public Health Clinics *n*=8,173 HIV tests *n*=14.269	0.22	1 in 455
Drug Users*** *n*=1.615	1	1 in 100
Homeless Population*** *n*=155	4.50	1 in 22
**Mexican Women** (ENSANUT 2012**)	**0.07** **0.23**	**1 in 1,428** **1 in 435**

* Prevalence of Project “Ponte a Prueba” 2010–2014.

** Seroprevalence of general population in Mexico between 15 yand 49 yars: reasults from Ensanut 2012. Article published at Salud Pública de México/vol. 56, no. 4, julio-agosto de 2014.

*** Prevalences from Jefatura de Unidad Departamental de Programas Específicos

# Sub article: P018 Implementation of new strategies for timely HIV diagnosis in Mexico City's Prison System and its impact on the continuum of care

**DOI:** 10.7448/IAS.19.2.21044

**Published:** 2016-04-28

**Authors:** Hugo Vargas González, Nathalie Gras Allain, Hector Cid Vazque, Jesús Casillas Rodríguez, Luis Manuel Jiménez Munguia, Alicia Piñeirua Menéndez, Florentino Badial Hernández, Andrea Gonzalez Rodríguez

**Affiliations:** 1 Clínica Especializada Condesa, Programa VIH/Reclusorios, Mexico City, Mexico; 2 Clínica Especializada Condesa, Subdirectora de Atención a Poblaciones Vulnerables, Mexico City, Mexico; 3 Clínica Especializada Condesa, Direccion/Consulta Externa, Mexico City, Mexico; 4 SSA Servicios Medicos Penitenciarios, Direccion, Mexico City, Mexico; 5 Clínica Especializada Condesa-Iztapalapa, Consulta Externa, Mexico City, Mexico; 6 Clínica Especializada Condesa-Iztapalapa, Direccion, Mexico City, Mexico; 7 Clínica Especializada Condesa, Directora Ejecutiva del programa VIH/SIDA, Mexico City, Mexico

## Abstract

**Introduction:**

In Mexico City, about 35,000 men are in prison, distributed throughout eight centres, with an HIV prevalence of 1.0% [1]. Prison inmates that are diagnosed with HIV are then transferred to the Santa Martha Acatitla (SMA) Prison, where they are offered highly active antiretroviral therapy (HAART) and specialized medical care. During the beginning of 2015, there were 192 patients at SMA, 54.9% of the total of estimated cases (*N*=350). During 2014, 4559 HIV tests were performed in all detention centres in Mexico City.

**Material and methods:**

During 2015, several innovative strategies were implemented aimed at prison inmates: in the two largest centres, voluntary HIV testing was offered to inmates by the correctional facility medical staff when they entered the prison. In one centre (the second largest), there was an HIV screening of all the prison population, while in another there was a health fair where HIV testing was offered to inmates as well as their family members. All prison centres received periodic visits of the HIV Mobile Diagnosis Team. The data were collected until 31 October 2015 in order to analyze the impact in the continuum of care of all new diagnosis.

**Results:**

A total of 20,535 HIV tests were performed from 1 January 2015 through 31 October 2015 in all the prison centres in Mexico City (4.5 times of all tests performed in 2014). Fifty-five new cases were diagnosed, along with 22 previously diagnosed cases, with an average CD4 count of 327 cell/mL (SD=258) (Figure 1). During this time, 56 patients were freed and two died. By 31 October, 216 inmates with HIV were aware of their HIV positive status (61.7% of the total estimated cases), 211 (60.3%) were incorporated to care, 210 (60.0%) retained in care and 204 (58.3%) received HAART. 190 (54.3%) patients had a viral load (VL) of <200 copies/mL and 175 (50.0%) had a VL of <40 copies/mL. 96.7% of the newly diagnosed patients are receiving HAART and 90% of patients on HAART have a VL of <40 copies/mL (83% with VL<200 copies/mL) (Figure 2).

**Conclusions:**

The HIV care programme in prisons has achieved two of the goals set by the WHO 90-90-90 initiative. The implementation of new diagnosis strategies has been translated into a significant increase in the number of HIV detections in prisons. The widespread implementation of these strategies in all the prison system of Mexico City may allow us to diagnose 90% of HIV cases among prison inmates.

**Abstract P018–Figure 1:** Immune level at HIV diagnosis. 59.8% with CD4>200

**Abstract P018–Figure 2.** The number and percentage of incarcerated people with HIV in different stages of care at the Santa Martha Acatitla Penitentiary in Mexico City during January – October 2015 with 50.0% in virology control (VL<40).

## References

[1] Gras AN, Badial HF, González RA Salud pública, VIH/SIDA y derechos humanos en los centros de reclusión.

# Sub article: P019 Incidence and time-varying predictors of HIV and sexually transmitted infections among male sex workers in Mexico City

**DOI:** 10.7448/IAS.19.2.21048

**Published:** 2016-04-28

**Authors:** Karla Y Ganley, Andrew R Zullo, Sandra G Sosa-Rubi, Carlos J Conde-Glez, Mark N Lurie, Brandon DL Marshall, Don Operario, Kenneth Mayer, Omar Galarraga

**Affiliations:** 1 Brown University, School of Public Health, Providence, USA; 2 Brown University School of Public Health, Health Services, Policy, and Practice, Providence, USA; 3 National Institute of Public Health, Center for Health Systems Research, Cuernavaca, Morelos, Mexico; 4 National Institute of Public Health, Center for Population Health, Cuernavaca, Morelos, Mexico; 5 Brown University School of Public Health, Epidemiology, Providence, USA; 6 Brown University School of Public Health, Behavioral and Social Science, Providence, USA; 7 Fenway Health and Harvard University, Global Health, Boston, USA

## Abstract

**Introduction:**

Male sex workers (MSWs) in Mexico are a particularly vulnerable population for acquisition of HIV/sexually transmitted infections (STIs). We estimated HIV/STI incidence rates and identified time-varying predictors of STI acquisition among MSWs in Mexico City.

**Methods:**

MSWs recruited from Clínica Condesa HIV Testing Clinic and community sites in Mexico City were tested and treated for STIs (chlamydia, gonorrhoea, syphilis and HIV) and viral hepatitis (hepatitis B and C) at a baseline, 6-month follow-up and 12-month follow-up clinic visits. Participants completed surveys at all visits to document socio-demographic characteristics and health behaviours. We estimated incidence rates and calculated 95% confidence limits using a bias-corrected and accelerated bootstrap method with 1000 replicates. We used mixed effects logistic regression with individual fixed effects to examine unadjusted and multivariable adjusted time-varying predictors of incident STIs (excluding HIV in order to retain participants with prevalent HIV infection).

**Results:**

Among 227 eligible participants, the median age was 24 and baseline HIV prevalence was 32%. Incidence rates were as follows: HIV (5.28 per 100 person-years (PY); 95% 2.25, 11.75), chlamydia (4.63 per 100 PY; 95% 2.10, 9.09), gonorrhoea (3.92 per 100 PY; 95% 1.88, 7.58), active syphilis (12.44 per 100 PY; 95% 8.17, 18.51), hepatitis B (2.13 per 100 PY; 95% 0.52, 5.01), hepatitis C (0.96 per 100 PY; 95% 0, 4.02) and any STI except HIV (21.00 per 100 PY; 95% 15.80, 30.69). In unadjusted mixed effects models, risk of incident STIs did not vary by older age, marriage, engagement in a stable romantic relationship, offering services to five or more clients, alcohol use, polysubstance use, sexual assault perpetration, condom use, provision of penetrative sex or provision of receptive sex (Table 1). However, risk of STIs did differ by educational attainment and was lower among those who completed high school (odds ratio (OR)=0.28, 95% 0.09, 0.85) or college/post-grade education (OR=0.13, 95% 0.03, 0.53) compared with those who had only completed primary or secondary schooling. The association between risk of STI infection and completion of high school (adjusted OR (AOR)=0.23; 95% 0.06, 0.93) or college/post-graduate education (AOR=0.17; 95% 0.03, 0.93) persisted in the multivariable adjusted model.

**Conclusions:**

Our findings suggest that HIV/STI incidence is high among MSW in Mexico City and should be a priority population for treatment and prevention interventions. Education appears to be an important potential predictor of HIV/STI infections and may be an important component of economic and structural interventions to prevent infections.

**Abstract P019–Table 1:** Unadjusted associations between selected time-varying factors and incident sexually transmitted infections

Characteristics	Odds ratio	95% confidence limits
Age greater than 24 years	0.76	0.30, 1.90
Educational attainment		
Primary or secondary school	**Reference**	**Reference**
High school	0.28	0.09, 0.85
College or post-graduate school	0.13	0.03, 0.53
Married	1.34	0.18, 10.17
Had stable romantic partner during last follow-up period	1.06	0.53, 2.13
Had vaginal, anal or oral sex with five or more clients last week	0.71	0.40, 1.27
Used alcohol while having sex with any of three most recent clients	0.81	0.33, 1.99
Used a substance (drugs or alcohol) while having sex with any of three most recent clients	1.45	0.54, 3.88
Perpetrated assault of any of three most recent clients	0.20	0.03, 1.36
Frequently used condoms during sex in the past month	0.77	0.30, 1.94
Had penetrative anal sex with any of 3 most recent clients	0.70	0.32, 1.55
Had receptive anal sex with any of 3 most recent clients	1.04	0.47, 2.30

# Sub article: P020 Changes in renal laboratory parameters and bone mineral density in treatment-naïve HIV-1-positive adolescents initiating therapy with INSTI-based single-tablet regimens containing tenofovir alafenamide (TAF) or tenofovir disoproxil fumarate (TDF)

**DOI:** 10.7448/IAS.19.2.21049

**Published:** 2016-04-28

**Authors:** Ellen Koenig, Aditya Gaur, Hilda Kizito, Wasana Prasitsuebsa, Natella Rakhmanina, Kulkanya Chokephaibu, Jan Fourie, Linda-Gail Bekker, Yongwu Shao, Sean Bennett, Anita Silva, Erin Quirk

**Affiliations:** 1 Infectious Disease, Instituto Dominicano de Estudios Virologicos, Santo Domingo, Dominican Republic; 2 Department of Infectious Disease, St. Jude Children's Research Hospital, Memphis, USA; 3 Joint Clinical Research Centre, NA, Kampala, Uganda; 4 Thai Red Cross AIDS Research Centre (HIV-NAT), NA, Bangkok, Thailand; 5 Children's National Medical Center, Infectious Diseases, Washington, USA; 6 Faculty of Medicine Siriraj Hospital, Mahidol University, NA, Bangkok, Thailand; 7 Dr Jan Fourie Medical Practice, NA, Dundee, South Africa; 8 The Desmond Tutu HIV Centre, University of Cape Town, The Desmond Tutu HIV Centre, Mowbray, South Africa; 9 Gilead Sciences, Biostatistics, Foster City, CA, USA; 10 Gilead Sciences, Clinical Research, Foster City, CA, USA; 11 Gilead Sciences, Public Health & Medical Affairs, São Paulo, Brazil

## Abstract

**Introduction:**

EVG/COBI/FTC/TAF [E/C/F/TAF] and EVG/COBI/FTC/TDF [Stribild, STB] are integrase inhibitor (INSTI)-based single-tablet regimens (STRs) in clinical development for HIV-1-positive adolescents. Exposures of all components have been shown to be within the range associated with antiviral activity in adults. Preliminary comparative safety data through 24 weeks are reported.

**Materials and methods:**

Treatment-naïve 12- to <18-year-olds weighing ≥35 kg with HIV-1 RNA >1000 copies/mL, CD4 >100 cells/µL and eGFR >90 mL/min/1.73 m^2^ received E/C/F/TAF or STB once daily in two ongoing 48-week, single-arm, open-label trials. Adverse events (AEs), laboratory tests, bone mineral density (BMD) by dual X-ray absorptiometry and height-age adjusted (HA) *Z*-scores were assessed through week 24.

**Results:**

The E/C/F/TAF and STB trials enrolled 50 and 33 adolescents, respectively (median age 15 vs. 16 years, 56% vs. 30% female, 88% vs. 76% black, 22% vs. 27% with baseline HIV-1 RNA >100,000 copies/mL, median CD4 count 456 vs. 407 cells/µL, median eGFR 156 vs. 143 mL/min/1.73 m^2^). Most AEs in both trials were mild and unrelated to treatment, with no deaths or AEs leading to treatment discontinuation. At week 24, the median increase in serum creatinine was +0.08 mg/dL in E/C/F/TAF participants and +0.10 mg/dL in STB participants, with median eGFR decreases of −17.0 and −18.0 mL/min/1.73 m^2^, respectively, consistent with COBI's inhibition of renaltubular creatinine secretion. Proteinuria (any grade) occurred in 26% of E/C/F/TAF participants versus 52% of STB participants, with Grade 2 or higher proteinuria occurring in 4% versus 21% of participants, respectively. Of those participants with BMD measurements at week 24, the median increase in spine BMD was +1.98% in E/C/F/TAF participants, with a decrease of ≥4% in 3/41 participants (7%), versus a median decrease of −1.29% in the STB cohort, with a decrease of ≥4% in 6/20 participants (30%). Spine HA *Z*-scores decreased by −0.02 and −0.21, respectively.

**Conclusions:**

Compared with STB, E/C/F/TAF exhibited similar effects on eGFR, a lower incidence and severity of proteinuria, and a median increase in spine mineralization. Both STRs were well-tolerated through 24 weeks. These findings support INSTI-based STRs as initial HIV-1 treatment in adolescents and suggest that TAF could offer safety advantages in paediatric populations.

# Sub article: P021 Salud a la Vida: developing HIV and hepatitis C prevention through community collaboration between a Hispanic-serving institution and its surrounding community

**DOI:** 10.7448/IAS.19.2.21050

**Published:** 2016-04-28

**Authors:** Britt Rios-Ellis, Mara Bird, Melawhy Garcia-Vega, Miguel Angel Ortiz Valenzuela, Dilia Ortega, Luis Cendejas

**Affiliations:** 1 Dean, College of Health Sciences and Human Services, Center for Latino Community Health, Seaside, USA; 2 NCLR/CSULB Center for Latino Community Health, Center Director, Long Beach, USA; 3 NCLR/CSULB Center for Latino Community Health, SDSU/UCSD Doctoral Student, Long Beach, USA; 4 NCLR/CSULB Center for Latino Community Health, CSULB, Long Beach, USA; 5 NCLR/CSULB Center for Latino Community Health, Bienestar, Long Beach, USA; 6 NCLR/CSULB Center for Latino Community Health, MPH Program Health Science, Long Beach, USA

## Abstract

**Introduction:**

HIV and hepatitis C (HCV) represent two of the greatest viral health challenges facing Latinos in the United States. While comprising 17% of the U.S. population, Hispanics account for 21% of HIV diagnoses (CDC, 2016). Furthermore, HCV rates among Latinos rose to 21.4% in 2011 (CDC, 2016). Another alarming trend can be observed in the age of HIV diagnosis with 24% of new infections seen in Latinos aged 13 to 24 compared with only 16% of whites (CDC, 2012).

**Materials and methods:**

As access to higher education increases for Latinos, Hispanic Serving Institutions (HSIs), in particular, provide a welcome venue to conduct HIV and HCV screening and prevention education. Furthermore, because HSIs are more likely to be populated with commuter students and more often located in regions wherein Latinos comprise a substantive portion of the population, the opportunity to create community-campus prevention partnerships has broad potential for substantive education. To optimize this possibility, the U.S. Department of Health and Human Studies Substance Abuse and Mental Health Services Administration (SAMHSA) initiated a community-campus programme development initiative linking HIV and HCV. Salud a la Vida was developed to create a lasting University-Community Based Organization partnership designed to build capacity on the California State University Long Beach (CSULB) campus and within the Long Beach community to deliver effective, integrated HIV and hepatitis C, alcohol and substance use intervention targeting Latino young adults (aged 18 to 24 years). Through implementation of a culturally relevant intervention, youth partners from both community-based organizations and the campus environment worked to conduct a needs assessment regarding community–campus HIV/HCV knowledge, access to screening and prevention needs.

**Results:**

Ten focus groups (*n*=53) involving Latino heterosexual males, females, transgender male-to-females and gay males aged 18 to 24 in both university and community settings were held to assess HIV and HCV knowledge, attitudes, testing history, community needs and risk. Transcript analysis revealed basic HIV prevention knowledge but a general inability to distinguish HCV and HIV. Furthermore, baseline HCV knowledge was extremely low and youth had little to no previous knowledge regarding screening or risk. Alcohol and marijuana use was common, and 93% of participants had not used a condom at last sexual intercourse.

**Conclusions:**

Focus group results in collaboration with peer community health worker recommendations were used to establish the Salud a la Vida HIV/HCV outreach education programme. Focus group findings and preliminary programme results will be presented.

## References

[1] (2014). Latinos and HIV/AIDS.

[2] Centers for Disease Control and Prevention (2012). HIV Surveillance Supplemental Report, Vol. 17, No. 4. Centers for Disease Control and Prevention.

[3] Centers for Disease Control and Prevention (2014). Hispanics/Latinos.

# Sub article: P022 Late diagnosis and adherence problems as determinants of AIDS mortality in Argentina: profile of people who died from AIDS in the Metropolitan area of Buenos Aires in 2010

**DOI:** 10.7448/IAS.19.2.21051

**Published:** 2016-04-28

**Authors:** Adriana Durán, Eduardo Perez, Ariel Adaszko, Valeria Levite, Sebastián Nardi, Marcelo Vila, Clarisa Brezzo, Carlos Guevel, Mercedes Fernández

**Affiliations:** 1 HIV-AIDS and STD Program, Ministry of Health, Buenos Aires, Argentina; 2 PAHO, WHO, Buenos Aires, Argentina; 3 UNAIDS, UNAIDS, Buenos Aires, Argentina; 4 Health Statistic and Information Program, Ministry of Health, Buenos Aires, Argentina

## Abstract

**Introduction:**

The early diagnosis of HIV as well as the access to the highly active antiretroviral therapy (HAART) of people living with HIV is key to avoid AIDS mortality. In our country, in spite of the fact that HAART is available for free, and active policies to promote testing have been implemented, 1400 people die from AIDS every year. It is essential to know the characteristics of people who die due to causes related to AIDS in order to define strategic interventions in the most vulnerable groups.

**Materials and methods:**

A retrospective observational study was carried out among people over 18 years old who died from a basic cause related to AIDS during 2010 in the Metropolitan Area of Buenos Aires. A collection tool was used to gather information about medical records of hospitalized people and outpatients. Absolute and relative frequency rates were obtained and tests of statistical significance in the analyzed variables were calculated whenever appropriate.

**Results:**

A total of 331 deaths were studied, out of which 64% took place in the Autonomous City of Buenos Aires. The average age of the people studied was 40 years and 64% were men; 71.3% lived in areas that belong to the province of Buenos Aires. The main mode of transmission was unprotected opposite-sex sexual intercourse, both in men and women. 31% of the deaths were of people who had a late diagnosis, and 40% of them were diagnosed while in the same hospital in which death occurred. The median period of time since the diagnosis and death was of five years, and it was shorter in men than in women (*p*<0.05). The main causes of deaths in confirmed diagnoses were tuberculosis, both pulmonary and disseminated, and meningeal cryptococcosis. The presence of a great number of defining diseases and the clinical and immune deterioration was a common result in all the hospitalizations that were analyzed. The prevalence of hepatitis B and C infections was of 28.4% and 39.4%, respectively.

**Conclusions:**

A high percentage of people who died from AIDS in The Metropolitan Area of Buenos Aires during 2010 had been late diagnosed, which appears to be one of the main characteristics of the deaths. The studied population had irregular contact with the health system, as to receive an early diagnosis as well as a proper outpatient's treatment.

# Sub article: P023 Prevalence of HIV infection among imprisoned women in Brazil: a cross-sectional survey in the State Prison System of São Paulo

**DOI:** 10.7448/IAS.19.2.21052

**Published:** 2016-04-28

**Authors:** Tânia Regina Correa Souza, Alberto Novaes Ramos Jr, Wedja de Almeida Sparinger, Márcia Teresinha F Santos, Maria Aparecida Silva, Anna Luiza Placco, Samantha Moreira Lamastro, Solange Medeiros Pongelupi, Carmen Silvia B Domingues, Maria Clara Gianna

**Affiliations:** 1 São Paulo State Program for STDs and AIDS, STD and AIDS Referral and Training Center, São Paulo State Department of Health, São Paulo, Brazil; 2 School of Medicine, Federal University of Ceara, Department of Community Health, Fortaleza, Brazil; 3 Health Coordination of the Prison System, São Paulo State Department of Penitentiary, São Paulo, Brazil

## Abstract

**Introduction:**

The increase of 78.3% of the female prison population in Brazil in the period 2005 to 2013 has brought great challenges, especially those related to the vulnerability to sexually transmitted infections [1]. Gender inequality, stigma and discrimination increase imprisoned women's vulnerability to HIV infection [1,2]. The State of São Paulo (SSP) accounts for 30% of this Brazilian population and has sought to develop actions within the National Policy for Integral Women's Health [3]. The aim of this study was to estimate the prevalence of HIV infection in women deprived of freedom in SSP, to establish parameters for monitoring and evaluation.

**Materials and methods:**

Cross-sectional seroepidemiological survey for HIV infection was conducted in 19 female prisons of SSP. This study was coordinated by the State Program of STD/AIDS in São Paulo and conducted from August 2012 to December 2013. The estimated population basis was 11,530 women in all prison units.The stages of the study include counselling, information about the intervention, guidance on the sexually transmitted diseases, free and informed consent to data collection and the offer of testing for HIV and syphilis. For the definition of HIV infection status, we used rapid diagnostic tests. Data analysis was based on the estimated prevalence with calculation of confidence intervals.

**Results:**

Of the total 8821 (76.5%) addressed women, 8740 (99.1%) underwent rapid diagnosis for HIV testing. The estimated prevalence of HIV infection from this evaluation was 2.84% (95% confidence interval [CI]: 2.46% to 3.16%), 248 infected women. HIV infection by prison unit in São Paulo varied from 0% (CRF and CRF Araraquara Rio Claro) to 10.20% (PF Capital – 95% CI: 7.64% to 12.76%). The integrated analysis identified co-infection (HIV and *Treponema pallidum*) in 36 women, estimated prevalence of 0.47% (95% CI: 0.33% to 0.61%).

**Conclusions:**

This is the first study-based action in this extension in SSP. This population-based survey reinforces the status of great vulnerability to HIV infection for women in the State penal system, both in urban and rural realities. This study brings as possibilities to be assigned: establishment of flowcharts and indicators for monitoring and evaluation of preventive and therapeutic strategies to this population, establishment of appropriate flowcharts to the State Epidemiological Surveillance System, definition of elements for interventions in the prison system addressing public health policies and scientific knowledge production from this and other nested publications to the project [3,4]. These initiatives could ultimately contribute to improving the quality of life of women with a neglected and vulnerable condition [2,3,4].

## References

[1] Ramos NA Jr AN, Placco AL, Santos MTF, Silva MA, Lattari MCT, Matida LH (Organizadora) O HIV e a Sífilis no Sistema Prisional Feminino do Estado de São Paulo. São Paulo, Secretaria de Estado da Saúde, 2015.v 64 pgs.

[2] Brasil (2007). Política Nacional Integral à Saúde da Mulher – princípios e diretrizes. http://conselho.saude.gov.br/ultimas_noticias/2007/politica_mulher.pdf.

[3] São Paulo (2013). Secretaria da Administração Penitenciária do Estado de São Paulo. Coordenadoria de Reintegração Social e Cidadania. Departamento Penitenciário Nacional do Ministério da Justiça. Manual de Diretrizes de Atenção à Mulher Presa, São Paulo.

[4] São Paulo (2012). Centro de Referência e Treinamento em DST/Aids. Planos Estratégicos – Programa Estadual DST/AIDS/SP. http://www.saude.sp.gov.br/resources/crt/publicacoes/publicacoes-crt/plano2012.pdf.

# Sub article: P024 HIV cascade of care and viral load suppression in trans-women and men who have sex with men populations in the Dominican Republic

**DOI:** 10.7448/IAS.19.2.21053

**Published:** 2016-04-28

**Authors:** Robert Paulino, Mayra Rodriguez-Lauzurique, Ricardo Domingo, Leandro Tapia, Paola Peña, Jose A Duran

**Affiliations:** 1 Universidad Iberoamericana, Research Department, School of Medicine, Santo Domingo, Dominican Republic; 2 Centro de Orientacion e Investigacion Integral-COIN, Psychology, Santo Domingo, Dominican Republic; 3 Universidad Iberoamericana, School of Medicine, Santo Domingo, Dominican Republic

## Abstract

**Introduction:**

The overall rate of new HIV infections appears to be in decline worldwide; however, among key populations, men who have sex with men (MSM) and trans-women (TGW), new HIV infections continue growing [1]. Since the introduction of highly active antiretroviral therapy (HAART), people living with HIV live longer than ever before. The HIV cascade of care is a comprehensive monitoring tool to evaluate the HIV continuum of care and to evaluate “leakage points” along the points of attention [2]. The objective of this study was to evaluate the HIV cascade along the services in MSM and TGW and compare this with the general HIV population in an outpatient clinic in Santo Domingo, Dominican Republic.

**Materials and methods:**

We used a retrospective database of one outpatient clinic for HIV patients to assess our population-based cascade. Paper-based data were collected from clinical files. Characterization of target populations was based on past clinical history or self-identification as exclusively MSM or TGW and compared with the HIV-positive with unknown risk (+WUR). Treatment access and administration of HIV drugs was based according with the Dominican Republic Ministry of Health criteria (<350 cell/µL) and viral suppression below 20 copies/mL.

**Results:**

We identified 405 HIV diagnosed individuals during the study period; 60% were male. Of these, 25.18% (*n*=102) were MSM and TGW. Overall, 64.44% in the +WUR was retained in care versus 65.68% in MSM/TGW (Figure 1). HAART access in +WUR was 56.04%, while in MSM/TGW 38.29%. Viral suppression in +WUR was 43.70% and in MSM/TGW 24.50%.

**Conclusions:**

We found a significant difference between access to HAART and viral suppression in both groups. It is necessary to note that higher viral loads among these groups will be of significant influence for HIV persistence in social and sexual networks. Drops in the proportion to be achieved in each step may be a reflection of challenges specific to MSM/TGW access to care. It is necessary to evaluate the potential role of antiretroviral and hormone replacement therapy interactions.

**Abstract P024–Figure 1:** Cascade of Care for MSM/TGW living with HIV in the Dominican Republic.

## References

[1] PEPFAR (2012). Blueprint for Creating an AIDS-Free Generation. http://www.pepfar.gov/documents/organization/201386.pdf.

[2] Hogg L, Lima V, Sterne JA, Grabar S, Battegay M, Bonarek M (2008). et al. Life expectancy of individuals on combination antiretroviral therapy in high-income countries: a collaborative analysis of 14 cohort studies. Lancet.

# Sub article: P025 Dyslipidaemia in transgender women before and after cross-sex hormone treatment and ART

**DOI:** 10.7448/IAS.19.2.21054

**Published:** 2016-04-28

**Authors:** Esmeralda Román, Armando Sánchez, Edgardo Hamid Vega, Jeremy B Cruz, Andrea Gonzalez Rodríguez, Pablo Belaunzarán Zamudio

**Affiliations:** 1 Clínica Especializada Condesa, Clínica Trans, Mexico City, Mexico; 2 Clínica Especializada Condesa, Salud Mental, Mexico City, Mexico; 3 Centro para la Prevención y Atención Integral del VIH/SIDA del Distrito Federal, Dirección, Mexico City, Mexico; 4 UNAM, División de Investigación, Mexico City, FM, Mexico

## Abstract

**Introduction:**

Transgender women (TGW) are a key population in the HIV epidemic. Most of these women use hormones, which may lead to a higher cardiovascular risk. It is unknown whether the use of hormonal therapy (HT) in combination with antiretroviral drugs (ARTs) potentiates even greater changes in lipids. In this study, we describe the prevalence and cumulative incidence of dyslipidaemia in TGW receiving HT, ARTs or both at Clinica Condesa in Mexico City.

**Methods:**

This is an observational cohort study. We retrieve data on total cholesterol (TC), triglycerides (TG), high-density cholesterol (HDL) and low-density cholesterol (LDL) of TGW receiving care at Clínica Condesa (CC), at baseline and the last available measurement.

**Results:**

We included 697 TGW receiving care in the Transgender Clinic of CC between January 2009 and January 2016 with available baseline lipid profile (60% of all TGW receiving care); 236 (33%) were HIV positive. At baseline, 38 (5.5%) had hypercholesterolaemia (>239 mg/dL): six (2.5%) HIV positive and 32 (7%) HIV negative (*p*=0.02); 142 (20%) had hypertryglyceridemia (>200 mg/dL): 92 (39%) HIV positive and 50 (11%) HIV negative (*p*<0.001). Also, 265 (38%) had low-HDL levels (<40 mg/dL): 114 (48%) HIV positive and 151 (33%) HIV negative (*p*=0.008); and 34 (4.9%) had high-LDL (>160 mg/dL): 4 (1.7%) HIV positive and 30 (6%) HIV negative (*p*=0.007). There were 341 (49%) subjects with follow-up lipid profile measurements at their last visit, of whom 83 (24%) were HIV positive; 236 of 258 HIV-negative TGW (91%) were receiving HT. Among HIV-negative TGW, 13 (5%) had hypercholesterolaemia, 51 (20%) hypertriglyceridemia, 89 (34%) low-HDL cholesterol and 12 (5%) high-LDL. Eighty (96%) HIV-positive subjects were receiving ART and 73 (88%) HT. Four (5%) HIV-positive TGW presented hypercholesterolaemia during follow-up, 22 (26%) hypertriglyceridemia, 34 (41%) low-HDL and 2 (2.4%) high-LDL. The proportion of TGW with hypertriglyceridemia was not different by HT group.

**Conclusions:**

The most common dyslipidaemia in TGW receiving care at CC before and during HT and ART were low levels of HDL followed by hypertriglyceridemia. HIV-infected TGW had a higher baseline prevalence of hypertriglyceridemia and low HDL. The proportion of patients with hypertriglyceridemia and low-HDL increased in HIV-negative TGW during follow-up and decreased in HIV-infected TGW, possibly due to the timely intervention with dietary treatment and effect of ARTs. There was no increase in lipid abnormalities in patients using HT, ART or both.

# Sub article: P026 Prevalence of elevated depression symptoms and key associated factors among female sex workers and women living with HIV in the Dominican Republic

**DOI:** 10.7448/IAS.19.2.21055

**Published:** 2016-04-28

**Authors:** Christine Rael, Alissa Davis

**Affiliations:** Columbia University, HIV Center for Clinical and Behavioral Studies, New York, USA

## Abstract

**Introduction:**

Little is known about the mental health of female sex workers (FSW) and women living with HIV (WLWH) in the Dominican Republic (DR), which may impede the effectiveness of HIV prevention, testing and treatment programming. This is important, since studies across multiple sociocultural contexts show that the prevalence of depression in FSW ranges from 50% to 80%. Depression is also prevalent in WLWH, ranging from 25.8% to 81.0%. There is almost no scientific work about depression in FSW and WLWH in the DR. This is problematic, since the DR is home to a generalized HIV epidemic (0.8% to 1.5%) and a concentrated HIV epidemic for FSW (3.3% to 8.4%), making this a high priority area for study.

**Materials and methods:**

This study estimated the prevalence of elevated depression symptoms and identified key correlates in FSW, WLWH and a comparison group. Participants were FSW (*N*=349), WLWH (*N*=213) and a comparison group of HIV-negative women who were not sex workers (*N*=314) from San Felipe de Puerto Plata, DR. Participants completed questionnaires assessing demographics and depression. FSW and WLWH completed items ascertaining HIV or sex work-related internalized stigma. Descriptive statistics summarized the demographic characteristics of the sample, and chi-square (categorical variables) and *t*-tests (continuous variables) detected significant differences between the comparison group and FSW or WLWH. Unadjusted and adjusted logistic regressions identified significant relationships between covariates and the outcome variable, elevated depression symptoms (EDS). Interaction terms were calculated to determine the strength of association of significant relationships with (1) FSW and the comparison group and (2) WLWH and the comparison group.

**Results:**

EDS were prevalent across all three populations under study: FSW (70.2%), WLWH (81.1%) and the comparison group (52.2%). Internalized stigma increased the odds of EDS for FSW (AOR=2.73; 95% CI=1.95 to 3.84) and WLWH (AOR=3.06; 95%CI=1.86 to 5.05). Higher permanent income lowered the odds of this outcome for FSW (AOR=0.08; 95%CI=0.01 to 0.80) and comparison group women (AOR=0.04; 95%CI=0.00 to 0.45). Interaction terms revealed that being a member of the FSW or WLWH group did not strengthen or weaken the relationships between covariates and EDS.

**Conclusions:**

The high prevalence of EDS across all three groups suggests that interventions to treat depression and its causes could benefit all women, regardless of HIV status or HIV risk. Interventions focused on addressing internalized stigma for FSW and WLWH could be particularly effective in reducing EDS in these two groups. Additionally, future depression-related interventions in the DR should address poverty, since this appears to play an important role in mental health.

# Sub article: P027 Trends of the HIV/AIDS epidemic in men who have sex with men in the state of São Paulo, Brazil

**DOI:** 10.7448/IAS.19.2.21056

**Published:** 2016-04-28

**Authors:** Mariza Vono Tancredi, Carmen Silvia B Domingues, Angela Tayra, Marcia Cristina Polon, Maria Clara Gianna

**Affiliations:** 1 CRT DST/AIDS - SP, Epidemiology, São Paulo, Brazil; 2 CRT DST/AIDS - SP, Director, São Paulo, Brazil

## Abstract

**Introduction:**

Trends study allows us to perform future projections and anticipate results on the HIV/AIDS epidemic trends. The objectives were to describe and analyze the AIDS and the HIV-positive cases trends in the state of São Paulo, in teenagers and adults, during the period of 2004 to 2013, according to age groups and men who have sex with men (MSM).

**Materials and methods:**

Trends study was performed with AIDS and HIV-positive notification data compared by age group and exposure categories. It was considered as a dependent variable (Y), the annual number of HIV-positive patients and the number of AIDS cases, in each of the studied categories, and the independent variable (X) was the time, represented by the calendar years, concerning the study period. The goodness of fit via R^2^ and *p*<0.05 were used to determine which models and data were most appropriate.

**Results:**

Among the MSM, 15,810 cases of AIDS and 16,138 cases of HIV-positive patients were analyzed. The obtained modelling was of first order to all age groups. The trends of the HIV-positive patients were growing in all age groups during the period of 2004 to 2013. The trends were linearly growing in the age groups of 13 to 19 years (Y=5+16X, R^2^=0.78, *p*=0.032); 20 to 29 years (Y=13+63X, R^2^=0.89, *p*=0.001); 30 to 39 years (Y=177+49X, R^2^=0.86, *p*=0.002); 40 to 49 years (Y=60+22X, R^2^=0.92, *p*<0.001) and 50 years and more (Y=17+8X, R^2^=0.82, *p*<0.001). However, in the AIDS cases, growing trends were observed only in the age groups of 13 to 19 years (Y=13+3X, R^2^=0.52, *p*=0.050) and in the 20 to 29 years group (Y=63+22X, R^2^=0.93, *p*<0.001). In the age groups of 30 to 39 years, 40 to 49 years and 50 years and more, the trend analysis of the AIDS cases revealed stability along the historical series.

**Conclusions:**

During the period of 2004 and 2013, it was observed among the HIV-positive patients that the higher growth rate occurred among the MSM in the age group of 20 to 29 years, followed by the age group of 30 to 39 years. Among the AIDS cases, it was observed that the MSM in the age group of 20 to 29 years also presented the highest growth rate. This analysis points to the importance of this most vulnerable group that are the young MSM and the need to prioritize them, planning actions of the epidemic control programmes.

# Sub article: P028 Patient-centred HIV care in Mexico City: basis for a primary care model and its potential impact on the cascade of care

**DOI:** 10.7448/IAS.19.2.21057

**Published:** 2016-04-28

**Authors:** Jesus Casillas Rodriguez, Diana Molina-Martinez, Andrea Gonzalez Rodríguez

**Affiliations:** 1 Clinica Condesa, Internal Medicine, Mexico City, Mexico; 2 Mexico City HIV Program, Executive Direction, Mexico City, Mexico

## Abstract

**Introduction:**

Mexico has a concentrated epidemic with an estimated 180,000 HIV-positive individuals in 2014, which translates to a prevalence of 0.3%. Among Mexico's 32 states, Mexico City has consistently had the highest cumulative HIV-AIDS incidence, being the oldest epidemic so far. Considering HIV a chronic disease, public health strategies should be implemented to optimize medical care programmes in ambulatory settings.

**Materials and methods:**

Condesa Clinic is Mexico City's ambulatory HIV unit that assists more than 10,000 active patients up to January 2016. We describe a new medical protocol to detect, evaluate and incorporate new HIV patients to the clinic. This protocol has been implemented since 2012 and is updated yearly according to results. We started an early incorporation to medical assistance and a time reduction strategy to start HAART, which potentially will reduce the number of lost patients (Figure 1).

**Results:**

As of January 2016, 10,459 patients are assisted in Condesa Clinic, 95.5% on treatment (*n*=9,990) and 4.48% (*n*=469) on follow-up. With more than 2000 new HIV-positive diagnosed every year from 2013 to 2015, we evaluated the effects of implementing the new strategy on retention and therapy rate. From 2208 new HIV detections in 2013, 30.1% patients were lost (30.1%, 666), half of them before medical evaluation. There were 1839 medical evaluations and incorporation to medical assistance (57.3%) and 841 initiated HAART in the following week (79.7%); 478 patients were referred to other centres (25.9%). The strategy also includes 8-week viral load to detect early virological failures. With this strategy, the comparative time to undetectability in those starting HAART was reduced from 12.9 (2009) to 5.9 months (2015). Of those on treatment, 99.7% have had a viral load/CD4 determination in the last year, and those with more than 6 months of therapy (*n*=9,056), 89.2% (8,079) and 98.6% had viral load of <50 and <200 copies/mL, respectively.

**Conclusions:**

The new HIV Care algorithm implemented allows to detect, evaluate and retain more patients, reducing the time to undetectability and the potential of HIV transmission. This is the first approach to early incorporation of HIV patients into medical care and early therapy starting time.

**Abstract P028–Figure 1:** Integrated Care Model to detect, evaluate, treat and retain new HIV patients in Mexico City.

# Sub article: P029 Assessing mental health among recently diagnosed HIV patients at Condesa Clinic in Mexico City

**DOI:** 10.7448/IAS.19.2.21058

**Published:** 2016-04-28

**Authors:** E Hamid Vega-Ramirez, Victor Rodriguez, Jeremy B Cruz, Valeria Daniela Ferreya, Carolina Rocabert, Harumi Hirata, Andrea Gonzalez Rodríguez

**Affiliations:** 1 Mental Health Programme, Condesa Specialized Clinic, Mexico City, Mexico; 2 Condesa Specialized Clinic, Mexico City, Mexico

## Abstract

**Introduction:**

Several factors, such as psychosocial and uncontrolled HIV infection in the central nervous system, contribute to the onset of a mental disorder. Common mental disorders are more prevalent in people with HIV (i.e. depressive disorders, cognitive impairment and substance use disorders) [1]. The aim of this study is to describe the mental healthcare process and some preliminary results of recently diagnosed HIV patients at the Condesa Clinic that have been assessed by the Mental Health Programme (MHP).

**Materials and methods:**

We describe the model of care of the MHP incorporated into the HIV treatment cascade at the Condesa Clinic for recently diagnosed patients (Figure 1). We used univariate analysis for preliminary data.

**Results:**

On 2012, the MHP was created. Based on the international evidence [2] and the needs of the Condesa Clinic patients, the MHP developed a process that included early detection and treatment for mental disorders. Within the first week of initial medical assessment, patients recently diagnosed with HIV had an evaluation to detect any mental disorder that could impair adherence to HAART or impact patients’ quality of life. A clinical psychologist through a clinical interview based on DSM-5/ICD-10 criteria and valid psychometric instruments [3] performs this evaluation. If the patient had an adjustment disorder, he/she received a brief intervention. If the patient had a moderate or severe common mental disorder, he/she received psychopharmacological treatment by a psychiatrist; and if the patient had acute psychosis or suicidal ideation, he/she was referred to a psychiatric hospital. MHP has evaluated 2158 patients, most of whom were men (89.0%), middle aged (30.9±9.3), with 11.8 (±3.9) years of formal education and single (76.7%). The 37.9% did not have any mental disorder at the moment of the mental assessment, but 35.3% had an adjustment disorder, 8.8% a depressive disorder, 4.3% an alcohol use disorder, 2.7% an anxiety disorder and 11.0% other disorders. They also had a mean viral load of 293,922 (±903,286) copies/mL, and a mean CD4 count of 308 (±229) cell/µL.

**Conclusions:**

Incorporation of mental healthcare services to the HIV treatment cascade at first-level facilities could be feasible and probably effective in order to help retention, to adhere to HAART and to get undetectable viral loads in patients. Further analysis to detect mental health factors related to unfavourable outcomes in HIV patients should be performed.

**Abstract P029–Figure 1:** Healthcare flowchart of Mental Health Programme at Condesa Clinic.

## References

[1] Bing EG, Burnam MA, Longshore D, Fleishman JA, Sherbourne CD, London AS (2001). et al. Psychiatric disorders and drug use among human immunodeficiency virus-infected adults in the United States. Arch Gen Psychiatry.

[2] Sherr L, Clucas C, Harding R, Sibley E, Catalan J (2011). HIV and depression – a systematic review of interventions. Psychol Health Med.

[3] Ramasubbu R, Taylor VH, Samaan Z, Sockalingham S, Li M, Patten S (2012). et al. The Canadian Network for Mood and Anxiety Treatments (CANMAT) task force recommendations for the management of patients with mood disorders and select comorbid medical conditions. Ann Clin Psychiatry.

# Sub article: P030 High rate of linkage to care and antiretroviral therapy initiation in Haiti and improvements over time

**DOI:** 10.7448/IAS.19.2.21059

**Published:** 2016-04-28

**Authors:** Nancy Dorvil, Kelly Hennessey, Colette Guiteau, Vanessa Rivera, Jessy Devieux, Margaret McNairy, Patrice Severe, Adias Marcelin, Pierrot Julma, Sidney Attwood, Serena Koenig, Jean William Pape

**Affiliations:** 1 Les Centres GHESKIO, Clinical Trials Unit, Port-au-Prince, Haiti; 2 Clinical Research, Analysis Group, Boston, MA, USA; 3 Les Centres GHESKIO, Adult ARV Clinic, Port-au-Prince, Haiti; 4 Weill Cornell Medical College, Center for Global Health, New York, USA; 5 AIDS Prevention Program, Florida International University, Miami, FL, USA; 6 Les Centres GHESKIO, Data Management, Port-au-Prince, Haiti; 7 Division of Global Health Equity, Brigham and Women's Hospital, Boston, MA, USA; 8 Les Centres GHESKIO, Port-au-Prince, Haiti

## Abstract

**Introduction:**

High attrition during the period from HIV testing to antiretroviral therapy (ART) initiation is widely reported. Though treatment guidelines have changed to broaden ART eligibility and services have been widely expanded over the past decade, data on the temporal trends in ART initiation rates are limited.

**Materials and methods:**

We evaluated temporal trends and predictors of retention for each step from HIV testing to ART initiation over the past decade at the GHESKIO clinic in Port-au-Prince Haiti. The 22,638 patients >17 years of age who received a positive HIV test at GHESKIO from 1 March 2005 to 28 February 2015 were included. Patients were followed to determine if they received all steps for ART staging and initiated ART within 6 months after HIV testing.

**Results:**

A total of 22,638 patients (60% female, median age 35 years) were included. 15,756 patients (70%) had blood drawn for CD4 count – this increased from 31% in 2005 to 82% in 2014 (*p*<0.0001). The time from HIV test to blood draw decreased over time, from a median of 79 days (IQR: 30 to 182) in 2005 to 0 days (IQR: 0 to 14) in 2014 (*p*<0.0001). A total of 14,482 patients (92%) returned for CD4 count results; this increased from 93% in 2005 to 96% in 2014 (*p*=0.61). The time from blood draw to return for CD4 count result decreased over time, from a median of 8 days (IQR: 3 to 15) in 2005 to 6 days (IQR: 2 to 14) in 2014 (*p*=0.04). A total of 5,885 patients who were eligible for ART at presentation initiated treatment; this increased from 24% in 2005 to 95% in 2014 (*p*<0.001). The median time from return for CD4 count result to ART initiation decreased from 3 days (IQR: 0 to 19) in 2005 to 0 days (IQR: 0 to 0) in 2014 (*p*<0.001) (see Figure 1 for a bar chart of retention at different time points). Predictors of ART initiation included later year of HIV testing, older age and higher educational status.

**Conclusions:**

The proportion of patients newly diagnosed with HIV who initiate ART has increased over the last decade in Haiti. Over the same time period, services have been delivered more rapidly. ART is now provided to most patients on the day that CD4 count results are provided, with very high rates of treatment initiation.

**Abstract P030–Figure 1:** Bar chart of CD4 count completion and ART initiation rates by year.

# Sub article: P031 Median CD4 count increase at ART initiation in Ministry of Health of Mexico between 2010 and 2015

**DOI:** 10.7448/IAS.19.2.21060

**Published:** 2016-04-28

**Authors:** Marisol Valenzuela-Lara, Carlos Magis Rodríguez, Eduardo Becerril-Vargas, Eddie Antonio León Juárez

**Affiliations:** CENSIDA, DAI, Mexico City, Mexico

## Abstract

**Introduction:**

The global scale-up of antiretroviral therapy (ART) over the past decade represents one of the great public health and human rights achievements of recent times. Both the standardization and simplification of prescribing practices have been critical to ART scale-up. Actually, an antiretroviral regimen for a treatment-naïve patient generally consists of two nucleoside reverse transcriptase inhibitors in combination with a third active antiretroviral drug. These guidelines are supported by studies of effectiveness, durability, optimal tolerance and comfort.

**Materials and methods:**

The objective of this study was to assess trends in the median CD4 count, number of people living with HIV initiating ART and the proportion of people in each regimen according to the third ARV, between 2010 and 2015 in Mexico Ministry of Health (MoH). We examined transversal data from 63,970 adults (aged ≥15 years) who initiated HIV treatment programmes at 138 HIV care clinics in Mexico between 2010 and 2015. The clinics included are part of Mexico MoH. Patient information is routinely collected in the “Antiretroviral Management, Logistic and Surveillance System” (SALVAR in Spanish). We defined CD4 count “at ART initiation” as any measurement three months before or one month after ART initiation. Statistical analyses were carried out using STATA, version 11.1.

**Results:**

Between 2010 and 2015, the median ART initiation age was 33 years. The median CD4 count at ART initiation increased from 183 to 242 cells/µL, *k*-sample test on the equality of medians was performed, *p<*0.0001, and an increase of 9.8 cell/year was observed; sex disparities were reduced from a male–female difference of 52 cells in 2010 to 13 cells in 2015 (Figure 1). The number of persons initiating ART each month has increased from 720 in 2010 to 1,174 in 2015, year with the higherst annual changes observed in the last five years (Figure 2), (Table 1). In 2010, 52.9% of ART initiation schemes had efavirenz, in 2015 this figure was 80.1%.

**Conclusions:**

Guidelines in Mexico have been in change, especially in the last couple of years, and the impact of this change is observed in the increase of CD4 at ART initiation. According to national guidelines, efavirenz has been the preferred third ARV, and the increase in the use of this ARV shows a more standardized prescription. Still intensified efforts are needed to initiate ART more opportunely.

**Abstract P031–Figure 1:** Median CD4 count at ART initiation.

**Abstract P031–Figure 2:** ART initiation of adult patients by trimester.

**Abstract P031–Table 1:** ART initiation of people living with HIV

Year of ART initiation	2010	2011	2012	2013	2014	2015
*N*	8645	9002	10,181	10,593	11,457	14,092
Monthly average	720	750	848	883	955	1174
Annual change	–	4.13%	13.10%	4.05%	8.16%	23.00%

# Sub article: P032 Early diagnosis and continuity of care of people with HIV in Argentina: gaps to achieve the “90-90-90” goals

**DOI:** 10.7448/IAS.19.2.21061

**Published:** 2016-04-28

**Authors:** Adriana Durán, Eduardo Perez, Ariel Adaszko, Marcelo Vila, Clarisa Brezzo, Carlos Falistocco, Sebastián Nardi, Vanesa Kaynar, Marysol Orlando, Andrea Ayma

**Affiliations:** 1 HIV-AIDS and STD Program, Ministry of Health, Buenos Aires, Argentina; 2 OPS, WHO, Buenos Aires, Argentina; 3 UNAIDS, UNAIDS, Buenos Aires, Argentina

## Abstract

**Introduction:**

The strategies implemented in response to the HIV epidemic in Argentina have impacted positively on the incidence of new cases and on AIDS mortality. However, it is estimated that 30% of people are undiagnosed and coverage of antiretroviral therapy (ART) reaches only 60%. These data demonstrate gaps with the national goals. To analyze these gaps, the Office of AIDS of the Ministry of Health, with support from UNAIDS and PAHO, developed a study to assess the conditions of linkage and retention in care and the effectiveness of ART regimens in newly diagnosed people or in naïve patients.

**Materials and methods:**

In 2014, a concurrent cohort study was conducted with a convenience sample that was based on the jurisdictional distribution of new diagnoses of 2010. The data for each patient were gathered at the beginning of the study and 12 months later. For this work, the inclusion criterion was having had a new diagnosis in 2014.

**Results:**

At the time of submission of this abstract, 80% of the sample had been monitored (549 of the 682 patients); 471 (86%) were included as recently diagnosed and 78 (14%) as naïve patients. Nearly 72% of the patients with recent diagnosis were still on treatment one year after the follow-up in the same health care facility. Of the remaining 28%, 51% were lost to follow-up, 33% had changed facilities and 16% were deceased. Excluding deaths and considering that referrals to other centres are not lost to follow-up, it can be concluded that after a year, 82% of patients were still monitored. The overall percentage of deaths was 4.7%; 22 of the 26 deaths occurred within a year of diagnosis. In 14 cases, the cause of death was related to an HIV-AIDS event. Treatment was indicated to 434 patients but only 409 (94%) actually started it, 308 (71%) sustained it one year later and only 54% achieved undetectable viral load values within the year. High viral load was associated with difficulties in adherence (45%), resistance development (17%) and toxicity (4%). Only 70% regularly visited the pharmacy for medication.

**Conclusions:**

These results show that while over 90% of people diagnosed with HIV effectively initiated ART, the gap increases when assessing the sustainability and effectiveness of treatment after one year of follow-up. To achieve the universal access goals, problems linked to adherence should be addressed.

# Sub article: P033 Care model for HIV-positive pregnant women

**DOI:** 10.7448/IAS.19.2.21062

**Published:** 2016-04-28

**Authors:** Teresita de Jesús Cabrera López, Ubaldo Ramos Alamillo, Elena Langarica Naves, Florentino Badial Hernandez, Alicia Piñeirúa, Jeremy B Cruz, Steven Díaz, Andrea Gonzalez Rodríguez

**Affiliations:** 1 Clinica Especializada Condesa, Ginecología y Obstetricia, Mexico City, Mexico; 2 Clínica Especializada Condesa, Director Médico de la Clínica Especializada Condes, Mexico City, Mexico; 3 Clinica Especializada Condesa, Trabajo Social, Mexico City, Mexico; 4 Clinica Especializada Iztapalapa, Director Médico de la Clinica Especializada Iztapa, Mexico City, Mexico; 5 Clínica Especilizada Iztapalapa, Infectología, Mexico City, Mexico; 6 Clinica Especializada Condesa, Salud Mental, Mexico City, Mexico; 7 Centro de Prevención y Atención Integral del VIH/sida de la Ciudad de México, Subdirector de Prevención e Información, Mexico City, Mexico; 8 Centro de Prevención y Atención integral del VIH/sida de la Ciudad de México, Directora Ejecutiva, Mexico City, Mexico

## Abstract

**Introduction:**

Condesa Specialized Clinic's (CEC) OB/GYN area offers free diagnosis and treatment for women with HIV. In Mexico, HIV prevalence in pregnant women is 0.06% [1], and in CEC, it is 0.22%. The objective of this study is to describe a cohort of pregnant patients that received care at CEC.

**Materials and methods:**

Transversal, descriptive study with a cohort of pregnant women with HIV at CEC, from August 2013 through October 2015. Patients were referred from public health centres with some clinics located in the subway stations who had a reactive rapid HIV test. At CEC, other diagnostic tests for HIV confirmation, HBV, HCV, syphilis, viral load (VL) and CD4 count were performed. Patients with reactive tests are immediately evaluated by OB/GYN for HAART; they have their VL measured weekly until an undetectable VL (<40 copies/mL) and then are evaluated monthly. At the 37th week, they are referred to a hospital for determination of birthing technique.

**Results:**

Of 69 pregnant women, 59 gave birth to a live baby. Of these, 38 were diagnosed with HIV during pregnancy (group 1) and 21 already had an HIV-positive diagnosis prior to becoming pregnant (group 2). In group 1, HIV diagnosis was established at week 25.7 (SD=8) of pregnancy. In group 2, 42.8% had a VL<40 when received notification of pregnancy; 42.1% of group 1 received RAL+TDF/FTC; 31.5% received ATV/r+TDF/FTC. About 58.6% of group 2 received ATV/r+ or LPV/r, and 23.5% received TDF/FTC/EFV. Nearly 73.6% and 100% of group 1 and group 2 had a VL<40 at the end of the pregnancy, respectively; 94.7% of group 2 had a VL CV<1000 copies; in two patients, virologic control was not achieved due to a late HIV diagnosis during pregnancy, and 97.3% of group 1 had a C-section birth versus 80.9% in group 2. The rest were natural births. 18.4% of group 1 and 23.8% of group 2 were lost to follow-up after birth. In those who continued treatment, there have not been any cases of vertical transmission of HIV reported. 89.4% of patient in follow-up have some kind of birth control method (Table 1).

**Conclusions:**

HIV diagnosis in women in group occurs at advanced stages of pregnancy increasing the risk of vertical transmission. In Mexico, coverage of HIV testing during pregnancy is about 58% [2]. Better strategies must be implemented to increase the coverage of HIV testing in pregnant women.

**Abstract P033–Table 1:** Care model for HIV pregnant women

Variable	Without prior HIV diagnosis (*N*=38)	With prior HIV diagnosis (*N*=17)	Vertical transmission of HIV (*N*=4)
**Socio-demographic characteristics**			
*Age* (average)	24.1 years	25.4 years	19 years
*Education*	*N*%	*N*%	*N*%
Illiteracy	(5) 13.1	(0) 0	(0) 0
Primary	(12) 31.5	(3) 17.6	(0) 0
Secondary	(14) 38.8	(7) 41.1	(2) 50
High school	(4) 10.5	(5) 29.4	(2) 50
University	(3) 7.8	(2) 11.7	(0) 0
*Occupation*	*N*%	*N*%	*N*%
Housewife	(26) 68.4	(11) 64.7	(3) 75
Employed	(9) 23.6	(4) 23.4	(0) 0
Other	(2) 5.2	(2) 11.7	(0) 0
Student	(1) 2.6	(0) 0	(1) 25
*Marital status*	*N*%	*N*%	*N*%
Common marriage	(29) 76.3	(9) 52.9	(2) 50
Single	(8) 21	(6) 35.2	(1) 25
Married	(1) 2.6	(2) 11.7	(1) 25
*Partner*	*N*%	*N*%	*N*%
Serodiscordant	(12) 31.5	(9) 52.9	(4) 100
Concordant	(21) 55.2	(2) 11.7	(0) 0
Unknown	(5) 13.1	(6) 35.2	(0) 0
*Drug use*	*N*%	*N*%	*N*%
Yes, during pregnancy	(4) 10.5	(1) 5.8	(0) 0
**Characteristics of HIV infection**			
Initial viral load (copies/mL)	Median 10,490 c/mL (SD 141,677)	52.9% detectable Median 17,000 c/mL (SD 459,179) Only 47% undetectable (*n*=8)	100% detectable Median 11,749 c/mL (SD 13,276)
Initial CD4 (c/µL)	Median 336 c/µL (SD 189.5)	Median 366 c/µL (SD 277.7)	Median 316 c/µL (SD 99.9)
*HAART*	*N*%	*N*%	*N*%
RAL+TDF/FTC	(16) 42.1	(3) 17.6	(0) 0
ATV/R+TDF/FTC	(12) 31.5	(6) 35.2	(0) 0
LPV/R+3TC/AZT	(8) 21	(2) 11.7	(2) 50
TDF/FTC/EFV	(0) 0	(4) 23.5	(0) 0
LPV/R+ TDF/FTC	(2) 5.2	(2) 11.7	(2) 50
Weeks on HAART after HIV diagnosis (median)	Median 9 weeks (SD 8.1)	Median 18 weeks (SD 9.7)	Median 24 weeks (SD 6.7)
Final VL c/mL	73.6% undetectable (*n*=28), 26.3% detectable (*n*=10), median 429 (D.E. 10,361)	64.7% undetectable (*n*=11), 35.2% detectable (*n*=6), median 124.5 (D.E.189)	75% (*n*=3) undetectable, 25% detectable (57 c/mL)
Final CD4 c/uL	Median 352 (SD 168)	Median 418 (SD 207)	Median 185 (SD 180)
Quit treatment after giving birth	18.4% (*n*=7)	23.5% (*n*=4)	25% (*n*=1)
**OB/GYN results**			
*Number of pregnancies*	*N*%	*N*%	*N*%
First time	(13) 34.2	(10) 58.8	(4) 100
Second time	(10) 26.3	(3) 17.6	(0) 0
Third or more	(15) 39.4	(4) 10.5	(0) 0
*Pregnancy weeks at time of HIV diagnosis*	Median 25.7 weeks (S.D. 8.1)	Median 20 weeks (S.D. 9.8)	Median 14.3 weeks (S.D. 6.7)
*Pregnancy resolution*	*N*%	*N*%	*N*%
Natural birth	(1) 2.6	(3) 17.6	(1) 25
C-section	(37) 97.3	(14) 82.3	(3) 75
*OB/GYN complications*	_	Premature birth due to severe preeclampsia 5.8%	Premature birth due to HELLP syndrome 25%
*Baby weight at birth* (median)	2,663 g (SD 449)	2,568 g (SD 424.9)	2,436 g (SD 568)
*Birth planning method*	*N*%	*N*%	*N*%
Definitive (bilateral tubal obstruction)	(25) 65.7	(12) 70.5	(1) 25
Transdermal implants	(2) 5.2	(2) 11.7	(2) 50
IUD	(6) 15.7	(0) 0	(1) 25
None	(5) 13.1	(3) 17.6	(0) 0
HPV (cervical cytology)	(4) 10.5	(6) 35.2	(1) 25

## References

[1] Centro Nacional para la prevención y el control del VIH y el sida Censida informa 2013.

[2] Eliminación de la transmisión maternoinfantil del VIH y de la Sífilis en las Américas (2015). Actualizacin 2015.

# Sub article: P034 Are we doing enough preventive primary care for older HIV-positive patients? A survey in a third-level hospital in Mexico City

**DOI:** 10.7448/IAS.19.2.21063

**Published:** 2016-04-28

**Authors:** Brenda Crabtree-Ramirez, Juan Sierra-Madero, Carlos Madrigal-Iberri, Maria Jose Reyes-Fentanes, Yanin Caro-Vega

**Affiliations:** Instituto Nacional de Ciencias Médicas y Nutrición Salvador Zubirán, Infectious Diseases, Mexico City, Mexico

## Abstract

**Introduction:**

Life expectancy of people living with HIV has increased as it has become a chronic manageable condition. Primary care preventive services play a crucial role in older patients with HIV infection given the higher incidence of co-morbidities when compared with non-HIV population. We aim to assess the frequency of screening tests for malignant and non-communicable diseases in HIV-positive patients >50 years seen at INCMNSZ in Mexico City before and after an intervention designed to improve awareness among physicians.

**Methods:**

All HIV-positive patients aged >50 years were included. The application of internationally recommended primary care screening tests [1] was reviewed in clinical charts. We evaluated the frequency of tests prescribed in the last year and at least once since patients became 50 years old. Factors associated with better screening practices (defined as more than 80% of screening performed as recommended) were analyzed. We developed a checklist of screening tests, as a reminder for physicians in each visit during a follow-up period of 12 months. Frequencies of tests before and after this intervention were compared.

**Results:**

Of 1602, 355 (22%) patients were aged >50 years, 84.4% were men with a mean age at HIV diagnosis of 45.6 years and a median time in care of 10.9 years (SD 5.94). Only 7.5% of patients had >80% of non-communicable diseases related tests compared with 90% of complete HIV-care-related tests (syphilis, vaccination and PPD) before our intervention. After reaching 50 years during care, 87% of women had at least one mammogram and 91% cervical cancer screening; 61% of MSM had anal cancer screening; colorectal cancer screening was done in 24.5%; and cardiovascular risk was calculated in <10%. Frequency of densitometries was higher in women (55% vs. 16%; *p*<0.001) (Figure 1.A). In the last year of care, only vaccination, dyslipidaemia, hypertension and diabetes screening were achieved in >90% (Figure 1.B). Those with CD4 <200 c/mm^3^ at HIV diagnosis were more likely to have better screening (*p*=0.02). Neither smoking (*p*=0.2) nor family history of cancer (*p*=0.3) was associated with better performance of screening. After the intervention, screening remained low for malignancies (52% and 24% for cervical and anal cancer, 30% for mammograms, 7.4% for colorectal cancer) and densitometries (8%). Cardiovascular risk assessment improved to 79%.

**Conclusions:**

Cancer and osteoporosis screening were underperformed. Lipids, glucose and blood pressure were done following recommendations. Only cardiovascular risk assessment improved after our intervention. Barriers to improve preventive primary care practices in older HIV-positive patients need further evaluation.

**Abstract P034–Figure 1:** Proportion of screening tests among HIV positive adults.

## References

[1] Aberg JA, Gallang JE, Ghanem KG, Emmanuel P, Zingman BS, Horberg MA, Primary Care Guidelines for the Management of Persons Infected with HIV (2013). Update by the HIV Medicine Association of the Infectious Diseases Society of America. Clin Infect Dis.

# Sub article: P035 Human papilloma virus and anal associated lesions in men who have sex with men, HIV-positive and HIV-negative: first cross-sectional study in Uruguay

**DOI:** 10.7448/IAS.19.2.21064

**Published:** 2016-04-28

**Authors:** Victoria Frantchez, Zaida Arteta, Cecilia D Albora, Dora Ruchansky, Benedicta Caserta, Ariel Cabrera, Susana Cabrera

**Affiliations:** 1 Hospital de Clínicas, Facultad de Medicina, Universidad de la República, Cátedra de Enfermedades Infecciosas, Montevideo, Uruguay; 2 Facultad de Medicina, Universidad de la República, Departamento de Bacteriología y Virología, Montevideo, Uruguay; 3 Red Integrada de Efectores Públicos de Salud, Laboratorio Conjunto de Citología, Montevideo, Uruguay

## Abstract

**Introduction:**

Gay and bisexual men, among other men who have sex with men (MSM), especially HIV-positive, have an increased risk of anal intraepithelial neoplasia, anal human papilloma virus (HPV) infection and anal cancer associated with HPV infection. Screening programme includes anal cytology (Pap test) and high resolution anoscopy and biopsy in patients with abnormal cytology findings [1,2]. Anal cytology screening is yet to be implemented as a routine in Uruguay, and there is no previous data of anal HPV infection prevalence in MSM. Our objective is to determine HPV genotypes prevalence in anal samples from HIV-positive and HIV-negative MSM and their association with anal lesions.

**Materials and methods:**

A cross-sectional study is being conducted since June 2015. Implementation period: one year. Sample: gay and bisexual men and other MSM over 18 years. Exclusion criteria: vaccinated against HPV and HPV-associated lesions previously diagnosed. Anal visual inspection is performed and two samples are taken from the anal canal for Pap cytology technique (Bethesda Classification 2001) [3], molecular diagnosis and genotyping of HPV (SerpaA). Patients with abnormal inspection or anal cytology are referred for anoscopy and eventual biopsy.

**Results:**

During the first six months of implementation: 38 patients were included. Median age: 34 years. Median age of first sexual intercourse: 18 years. Average number of sexual partners in the last six months was 14.5; 29 (76.3%) were HIV-positive, all on antiretroviral therapy, with median CD4 620/mm^3^ and 23 (79.3%) with suppressed viral load. Anal inspection was abnormal in 14 (36.8%) patients. Twenty-seven patients had a pathological cytology: 14 (36.8%) ASCUS, 12 (31.6%) LSIL, 1 (2.6%) HSIL and 9 (23.7%) normal cytology. Two samples were unrepresentative. The molecular diagnosis of HPV was positive in 23 (65.8%) patients, negative in 6 (17.1%) patients and unrepresentative in 6 (17.1%) patients. The genotypes found in 11 patients were 6, 11, 16, 18 and 54. Anoscopy was performed in 15 (55.5%) of 27 patients with indication for it: 9 without macroscopic alteration and 6 pathological (condilomatosis). HPV detection was positive in 18 (78.35) HIV-positive patients and 5 (21.7%) HIV-negative patients (*p*=0.555). Cytology was abnormal in 23 (85.2%) HIV-positive patients (14.8%) and 4 HIV-negative (*p*=0.064).

**Conclusions:**

A high prevalence of HPV infection and pathological anal cytology was found in the population studied, especially in HIV-positive patients. These results are similar to the ones found in international data and will help to develop national strategies of prevention through the implementation of screening.

## References

[1] Conley LJ, Bush TJ, Darragh TM, Palefsky JM, Unger ER, Patel P (2016). Incidence and predictors of abnormal anal cytology findings among HIV-infected adults receiving contemporary antiretroviral therapy. J Infect Dis.

[2] Machalek D, Poynten M, Jin F, Fairley CK, Farnsworth A, Garland SM (2012). Anal human papillomavirus infection and associated neoplastic lesions in men who have sex with men: a systematic review and meta-analysis. Lancet Oncol.

[3] Solomon D, Davey D, Kurman R, Moriarty A, O'Connor D, Prey M (2002). Forum Group Members; Bethesda 2001 Workshop. The 2001 Bethesda system. Terminology for reporting results of cervical citology. JAMA.

# Sub article: P036 Analysis of HIV/AIDS mortality in Mexico by state and municipal levels from 2003 to 2013

**DOI:** 10.7448/IAS.19.2.21065

**Published:** 2016-04-28

**Authors:** Enrique Bravo-García, Hilda Ortiz-Pérez

**Affiliations:** 1 Spectrum: Educación, Salud y Sociedad, A.C., President, Mexico City, Mexico; 2 Departamento de Atención a la Salud, Universidad Autonóma Metropolitana – Xochimilco, Mexico City, Mexico

## Abstract

**Introduction:**

Since 2003, Mexico has a policy of free and universal access to treatment for HIV/AIDS. At the end of 2013, a total of 89,000 persons were receiving highly active antiretroviral therapy (HAART). However, from 2003 to 2013, 54,140 persons have died from AIDS. The death rate for HIV/AIDS has not decreased as expected, reducing only from 4.4 deaths per 100,000 inhabitants in 2003 to 4.2 per 100,000 in 2013. The aim of the present study was to analyze the distribution of AIDS mortality according to state and municipal levels, with the purpose of identifying the sites where resources should be targeted to reduce HIV/AIDS mortality in Mexico.

**Materials and methods:**

We estimate the increase and decrease in the death rate from HIV/AIDS by state from 2003 to 2013 and the average annual HIV/AIDS mortality rate among 2,437 municipalities in Mexico from 2009 to 2013. Considering only the municipalities with 20,000 or more inhabitants, we selected 25 municipalities with the highest mortality rates. The information for these calculations was obtained from INEGI and CONAPO, institutions providing official figures of population and mortality, respectively.

**Results:**

In 2013, the states with the highest AIDS mortality rates were: Tabasco (10.58 per 100,000 inhabitants), Quintana Roo (9.02) and Veracruz (8.65). In contrast, Zacatecas (1.03), Guanajuato (1.43) and Hidalgo (1.92) recorded the lowest rates. The difference between the states with the highest and lowest rate (Tabasco and Zacatecas) is more than seven times. It is important to emphasize that, from 2003 to 2013, in 50% of Mexican states (16/32) the AIDS mortality rate increased, rather than decreased (Figure 1).

The 25 municipalities with the highest death rate from AIDS are located in five states, all of them in the Southeast (Veracruz, Chiapas, Tabasco, Campeche and Oaxaca). The municipality of Carlos A. Carrillo, Veracruz, ranked one with a rate of 39.6 HIV/AIDS deaths per 100,000 inhabitants (nine times higher than the national rate), and Poza Rica, Veracruz, ranked 25 with a rate of 13.4 HIV/AIDS deaths per 100,000 inhabitants (three times higher than the national rate) (Figure 2).

**Conclusions:**

Data highlight the need for enhanced HIV detection and treatment for 25 municipalities with the highest AIDS mortality rates, as well as the states most affected. Findings can help to apply interventions to link and retain in care persons until they are virologically suppressed. It is urgent to reduce AIDS mortality in Mexico.

**Abstract P036–Figure 1:** Percentage increase/decrease in AIDS mortality by state from 2003 to 2013.

**Abstract P036–Figure 2:** 25 municipalities with the highest AIDS mortality rates from 2009 to 2013.

# Sub article: P037 Significant increase in new HIV infections among young adults in Latin America

**DOI:** 10.7448/IAS.19.2.21066

**Published:** 2016-04-28

**Authors:** Carlos Gallo, Olga Lopez, Carolina Chahin, Beatriz Marincovich, Pedro Zitko, Carlos Beltran

**Affiliations:** 1 Latin American HIV Workshop Study Group, AIDS, Arica, Chile; 2 Latin American HIV Workshop Study Group, AIDS, Iquique, Chile; 3 Latin American HIV Workshop Study Group, AIDS, Temuco, Chile; 4 Latin American HIV Workshop Study Group, AIDS, Santiago, Chile

## Abstract

**Introduction:**

The WHO/UNAIDS 2.0 initiative and its 90-90-90 goal aim to reduce new HIV infections by 90% through a significant increase in diagnosis, care and ART of HIV-positive populations around the world. Most Latin American countries launched expanded access to ART a long time ago but showed low rates of diagnosis of HIV-positive people which explains, at least in part, the modest 3% reduction in new infections in the region as compared to a 38% global reduction and more than 50–75% reduction in some countries.

**Materials and methods:**

The Latin American Workshop Study Group is an expanding network of 38 HIV care centres from 11 countries of South America, Central America, the Caribbean and Mexico with clinical data from 73,431 patients up to September 2015, 7,732 of them being new 2013–2014 HIV cases. Age and gender distributions were analyzed globally and by participating centres and countries. Statistical analysis was done by chi-square test.

**Results:**

Among new HIV infections, 33.8% were 15–29 years old. This is the largest age group in all countries except Argentina with wide differences between centres in each country. 79.4% of new cases were men, with 34.5% of them 15–29 years old, and 20.6% were women, with 31.0% of them 15–29 years old (*p*<0.01). On the contrary, 14.1% of new HIV infections occurred in men older than 50 years as compared with 18.1% of new cases in women older than 50 years. In a sub-analysis in Chile, the largest increase in new cases among people younger than 30 years was observed between the age of 15 and 24 years.

**Conclusions:**

A modest reduction in new HIV infections has been reported in Latin America. Young people especially men between 15 and 24 years show an important increase in new HIV cases during 2013–2014 in most countries in spite of some differences between the centres. In the context of the 90-90-90 goal, specific policies should be implemented targeting this key population.

# Sub article: P038 Presentation to care with advanced HIV disease is still a problem in Latin America

**DOI:** 10.7448/IAS.19.2.21067

**Published:** 2016-04-28

**Authors:** Ana Paulina Celi, Maria Greco, Ernesto Martinez, Carmen Vargas, Francisco Belaunzaran Zamudio, Fernando Mejia

**Affiliations:** 1 Latin American HIV Workshop Study Group, AIDS, Quito, Ecuador; 2 Latin American HIV Workshop Study Group, AIDS, La Plata, Argentina; 3 Latin American HIV Workshop Study Group, AIDS, Cali, Colombia; 4 Latin American HIV Workshop Study Group, AIDS, San Jose, Costa Rica; 5 Latin American HIV Workshop Study Group, AIDS, Mexico City, Mexico; 6 Latin American HIV Workshop Study Group, AIDS, Lima, Peru

## Abstract

**Introduction:**

Presentation to care with advanced HIV disease, defined as first CD4 count below 200 cells/mm^3^, has been reported previously between 38 and 45% by PAHO, CCASAnet and the Latin American Workshop Study Group with significant differences between countries. The UNAIDS/WHO 2.0 initiative, beside 90-90-90 goal, aims to reduce late presentation to ART to 10% of new cases by increasing strategies to promote testing in different key populations and at a general level.

**Materials and methods:**

The Latin-American Workshop Study Group is an expanding network of 38 HIV care centres form 11 countries of South America, Central America, the Caribbean and Mexico with clinical data from 73,431 patients up to September 2015, 6,072 of them being new 2013–2014 HIV cases. Late presentation to care was analyzed globally, by gender, age and by participating centres and countries according to the first CD4 count and the clinical stage at the first visit. Statistical analysis was done by chi-square test and odds ratios.

**Results:**

Among new HIV infections, 37.0% presented to care with a CD4 count below 200 cells/mm^3^ and an additional 23.1% with a first CD4 count between 200 and 350 cells/mm^3^. 52.1% had diseases defining B or C stage. 37.5% of men and 35.1% of women presented with a CD4 count below 200 cells/mm^3^ (*p*<0.01; OR 0.26 for very late presentation among women). Late presentation was strongly associated to age at presentation. People younger than 30 years had the lowest risk for late presentation (*p*<0.01). Differences between countries remain.

**Conclusions:**

Very late presentation to care is a direct consequence of insufficient testing in Latin America. A very modest reduction in presentation to care with less than 200 cells/mm^3^ has been observed in new HIV cases in 2013–2014 in all countries in spite of some differences between centres. Women and people younger than 30 years have the lowest risk for presentation to care with advanced HIV disease. Strategies for increase in testing should address the higher prevalence in key populations but also promotion of testing in people at risk for late presentation.

# Sub article: P039 Dolutegravir-based regimens (DBRs) viral load decay at week 4 could predict sustained viral suppression at week 96

**DOI:** 10.7448/IAS.19.2.21068

**Published:** 2016-04-28

**Authors:** Romina Quercia, Steve Almond, Tia Vincent, Alicia Aylott, Michael Aboud, Jessica Lim

**Affiliations:** 1 ViiV Healthcare, Medical Department, London, UK; 2 Bayer, Mississauga, Canada; 3 Glaxosmithkline, Stockley Park, UK

## Abstract

**Introduction:**

The medium- or long-term implications of rapid viral load early-phase decay, during integrase inhibitor-based therapy, are not fully understood. This analysis was conducted to assess the predictive value of rapid virological response (RVR) at week 4 on sustained virological response (SVR) at week 96, in naïve subjects treated with dolutegravir-based regimens (DBRs).

**Materials and methods:**

Post hoc cross-sectional analysis of subjects enrolled in the naïve dolutegravir (DTG) Phase 3 clinical trials, SPRING-2 [1], SINGLE [2] and FLAMINGO [3]. RVR and SVR were assessed at weeks 4 and 96, respectively based on HIV-1 RNA<50 as determined by FDA snapshot. Positive predictive values (PPVs) and negative predictive values (NPVs) were calculated: PPVs as the proportion of subjects suppressed at week 4 who were also suppressed at week 96 and NPVs as the proportion of subjects not suppressed at week 4 who were also not suppressed at week 96.

**Results:**

A total of 2,139 subjects were analyzed including those receiving DBRs and comparator arms. The analysis revealed that 70% of the subjects receiving DBRs achieved RVR at week 4, 80% attained SVR at week 96. PPVs and NPVs of SVR in the DBR study population were 85 and 29%, respectively. The DBRs’ PPV was numerically higher than for Efavirenz (EFV) or Darunavir/ritonavir (DRV/r) plus 2 nucleosides (Nucs), and similar to that with Raltegravir (RAL) plus 2 Nucs. The NPV with RAL was numerically higher than with DBRs, reflecting that more DBR subjects without RVR ended with SVR.

**Conclusions:**

This analysis shows high rates of RVR with DBRs at week 4, translated into high PPV, suggesting potential predictability of week 4 RVR on SVR. Further analysis of long-term data would be key to support the relationship between early and long-term response. Higher RVR with DBRs, associated with high PPV for SVR, may have implications for early monitoring frequency.

**Table:** 

SVR at W96	SINGLE DTG–EFV	SPRING—2 DTG–RAL	FLAMINGO DTG–DRV/r
PPV (%)	87—73	84—85	82—70
NPV (%)	32—28	28—39	26—32

## References

[1] Raffi F, Jaeger H, Quiros-Roldan E, Albrecht H, Belonosova E, Gatell JM (2013). Once-daily dolutegravir versus twice-daily raltegravir in antiretroviral-naive adults with HIV-1 infection (SPRING-2 study): 96 week results from a randomised, double-blind, non-inferiority trial. Lancet Infect Dis.

[2] Walmsley S, Baumgarten A, Berenguer J, Felizarta F, Florence E, Khuony-Joses MA (2015). Dolutegravir plus abacavir/lamivudine for the treatment of HIV-1 infection in antiretroviral therapy-naive patients: week 96 and week 144 results from the SINGLE randomized clinical trial. J Acquir Immune Defic Syndr.

[3] Molina JM, Clotet B, van Lunzen J, Lazzarin A, Cavassini M, Henry K (2015). Once-daily dolutegravir versus darunavir plus ritonavir for treatment-naive adults with HIV-1 infection (FLAMINGO): 96 week results from a randomised, open-label, phase 3b study. Lancet HIV.

# Sub article: P040 Clinical monitoring system of people living with HIV/AIDS (SIMC): Brazilian strategy to reduce GAP treatment

**DOI:** 10.7448/IAS.19.2.21069

**Published:** 2016-04-28

**Authors:** Marihá Moura, Ana Kolling, Marcelo Freitas, Ana Roberta Pascom, Adele Benzaken, Fabio Mesquita

**Affiliations:** Department of STD/AIDS and Viral Hepatitis, Ministry of Health of Brazil, Brasilia, Brazil

## Abstract

**Introduction:**

Brazil published a normative protocol in 2013 implementing the “Treatment for All,” being the first developing country to recommend highly active antiretroviral therapy (HAART) for all people living with HIV/AIDS (PLWHA), regardless of CD4 and viral load. At the end of 2014, there were 781,000 PLWHA in Brazil, representing a prevalence rate of HIV of 0.39%. Of these, 83% were diagnosed and 52% started HAART. However, only 62% of the diagnosed PLWHA are on HAART, a major challenge of Brazil against AIDS epidemic.

**Materials and methods:**

In order to expand HAART coverage, Brazilian Ministry of Health developed in 2013 a clinical monitoring system (SIMC) from data of the logistics and laboratory control systems. SIMC shows treatment GAP and monthly monitors the inclusion of these patients into treatment. Treatment GAP means PLWHA who did CD4 and viral load tests and are not on HAART. According to the Brazilian’s Treatment Guideline, all people with HIV have indication of HAART, regardless of CD4.

**Results:**

Ministry of Health's technicians were responsible for monitoring, locally or at distance, estates and municipalities in their GAP elimination strategies.

**Conclusions:**

SIMC is an effective monitoring tool that allows services to identify GAP treatment and conduct active surveillance of patients for starting HAART. Since SIMC implementation, GAP decreased from 32.5% to 29%. A general reduction was observed in relation to age groups of PLWHA throughout the analysis period, with age group 18–24 having largest GAP (43.4%) (Figure 1). A total of 15 states retain GAP over the national average (29%) (Figure 2). These results allow focusing on upcoming interventions in places with the greatest difficulties to reduce GAP, considering the need to invest in actions aimed at young people and to scale up access to treatment.

**Abstract P040–Figure 1:** Treatment GAP of Brazilian states (aged 18 and over), 2015.

**Abstract P040–Figure 2:** Proportion of PLWHA eligible for HAART who did not start treatment (GAP) by age group. Brazil, 2009–2015.

# Sub article: P041 Low-level viraemia in people living with HIV at antiretroviral therapy

**DOI:** 10.7448/IAS.19.2.21070

**Published:** 2016-04-28

**Authors:** Marisol Valenzuela-Lara, Carlos Magis Rodríguez, Eduardo Becerril-Vargas, Eddie Antonio León Juárez

**Affiliations:** CENSIDA, DAI, Mexico City, Mexico

## Abstract

**Introduction:**

HIV viraemia is recognized as a significant prognostic indicator of disease progression in HIV-1-positive patients, and HIV viral load (VL) acts as a marker for treatment response. The goal of antiretroviral therapy (ART) is to reduce viral load to undetectable levels, below 50 copies/mL. However, these goals are not always achieved. Some patients experience persistent low-level viraemia (LLV), which may be considered as a significant prognostic indicator of virologic failure and promotes the selection of drug resistance mutations. Low-level viraemia is defined as VL between 50–200 copies/mL HIV-1 RNA.

**Materials and methods:**

The objective of this study was to estimate the prevalence of LLV in people with HIV who recently initiated antiretroviral therapy at the Ministry of Health of Mexico. A total of 3,091 patients were studied; they were under first-line therapy and reached 24 weeks under treatment in 2015 with a subsequent VL. VL results were related to clinical data retrospectively including ART-composition, and they were subdivided into non-nucleoside reverse transcriptase inhibitors (NNRTIs) (Efavirenz) and protease inhibitors (PIs) (Atazanavir, Darunavir, Lopinavir). Patients were classified into four groups:<50 copies/mL, 50–200 copies/mL, 201–500 copies/mL and =501 copies/mL.

**Results:**

Success of therapy was defined as <50 copies/mL and was observed in 407 (76.5%) women and 2,088 (81.6) men; the difference observed was statistically significant (*p*<0.0001). A bivariate analysis showed that men had a higher probability of achieving success than women (OR 1.36; CI95% 1.08 to 1.71; *p*=0.0068). The prevalence of LLV in women and men was 7.3 and 10.1%, respectively, and 9.6% for both sexes (Figure 1). The amount of patients with LLV differed significantly between NNRTI-based first-line regimens (9.17%) and PI-based regimens (11.62%) (*p*<0.0001); a bivariate analysis showed that patients on NNRTI-based regimens had a higher probability of achieving <50 copies/mL (OR 1.66; CI 95% 1.34–2.06; *p*<0.00001). When data were subdivided into ART-composition and sex, the difference between sexes were not statistically significant for NNRTI-based regimens (*p*=0.418), but they remain statistically significant for PI-based regimens (*p*<0.0001) (Figure 2).

**Conclusions:**

Regimens containing an NNRTI plus two NRTIs have been shown to suppress VL to lower HIV-1 RNA copy numbers compared to those with two NRTIs plus PIs, with important gender differences that have impact on women's ART outcomes.

**Abstract P041–Figure 1:** Low-level viraemia prevalence after 24 weeks on ART at Mexico MoH, 2015.

**Abstract P041–Figure 2:** VL after 24 weeks on ART at Mexico MoH by sex and regimen, 2015.

# Sub article: P042 Persistency of first-line ART in a real-world setting in a cohort of HIV-positive patients from Santiago, Chile

**DOI:** 10.7448/IAS.19.2.21071

**Published:** 2016-04-28

**Authors:** Leonardo Chanqueo, Fernando Bernal, Patricia Vásquez, Catalina Gutierrez, Carolina Giadalah, Michel Serri

**Affiliations:** Hospital San Juan de Dios, Servicio de Medicina, Santiago, Chile

## Abstract

**Introduction:**

Prolonging ART regimen durability is a key to achieving long-term treatment success in the management of HIV-positive patients. However, risk factors such as frequency of dosage, complexity of regimens and medication side effects may result in the discontinuation of ART. The aim of this study was to estimate the persistence of the most commonly used first-line ART regimens used in HIV-positive adults in Chile and the most common causes of changed ART in our cohort.

**Materials and methods:**

Using restrospective data from our cohort from Hospital San Juan de Dios de Santiago, Chile, we included naïve HIV patients between 2013 and 2014 who initiated ART with one of the following regimens: fixed-dose backbone (ABC/3TC, TDF/FTC or ZDV/3TC) plus EFV, ATV/r or LPV/r. The cumulative incidence of treatment change was calculated at 18 months. In addition, the distribution of the reasons for changing was calculated in patients who changed their initial ART regimen.

**Results:**

A total of 282 ART-naïve patients were included; 85% were male (80% men who have sex with men) and 15% female. A total of 104 (36.9%) started with ZDV/3TC, 99 (35.1%) with ABC/3TC and 79 (28%) with TDF/FTC as a backbone. The most commonly prescribed third drug was EFV (173, 61.3%) followed by ATV/r (59, 20.9%) and LPV/r (50, 17.7%). At 18 months of ART, 70% of the patients maintained the same regimen. Twice a day (BID) regimen was associated with a higher risk of treatment change compared with QD regimen; TDF/FTC plus (EFV or ATV/r) and ABC/3TC plus (EFV or ATV/r) regimens had a cumulative incidence of treatment change at 18 months of 0.148/0.197 and 0.214/0.250, respectively. However, BID regimens such as ZDV/3TC plus (EFV, ATV/r or LPV/r) had a cumulative incidence of treatment change of 0.483/0.400/0.516, respectively. The reasons for treatment change were toxicity/adverse event (*n*= 62, 66.1%) – hematologic toxicity (*n*=23, 25%), skin rash (*n*=13, 14%) and CNS side effects (*n*=11, 12%) – followed by simplification (*n*=12, 14%) and treatment failure/resistance (*n*=5, 6.5%).

**Conclusions:**

HIV patients initiating first-line ART with QD regimen are more likely to be persistent with the same therapy than those beginning treatment with BID regimen. Toxicity remains the main reason for discontinuing ART. Discontinuation and switching of initial ARV regimens are still frequent. Therefore, the choice of an easy and non-toxic ARV regimen for initial therapy is the most important issue in the care of our HIV patients.

# Sub article: P043 48 weeks CD4 cell recovery in HIV-positive patients on effective antiretroviral treatment

**DOI:** 10.7448/IAS.19.2.21072

**Published:** 2016-04-28

**Authors:** Guillermo Viloria, Mariana Kundro, Javier Toibaro, Marcelo Losso

**Affiliations:** J.M. Ramos Mejia, Servicio de Inmunocomprometidos, Buenos Aires, Argentina

## Abstract

**Introduction:**

The CD4+ cell counts recovery in HIV-positive individuals receiving antiretroviral therapy (ART) shows high variability. We aimed to evaluate the factors associated with successful CD4 recovery in the first year of treatment in a cohort of patients on effective ART.

**Materials and methods:**

We reviewed medical records of all naïve outpatients for whom ART was started between January 2008 and December 2014 in our unit. Pregnant women were excluded. CD4 recovery was defined as an increment >100 cells/µl in the first year of therapy, and effective treatment as the achievement of HIV-RNA <50 cop/ml. Logistic regression was used to examine the factors associated with CD4+ cells recovery.

**Results:**

A total of 463 patients started ART during the study period, of whom 418 (90%) achieved virologic suppression. Nine patients (1.9%) died. Data on CD4 recovery were available for 338 (82%) patients. Overall, 67.1% (227/338) of patients were male, median age was 41 years (IQR: 35–48), baseline CD4+ count was 183 cells/µl (IQR: 87–250) and median CD4+ gain at 48 weeks 196 cells/µl (IQR: 102-301). The proportion of patients with a successful immune recovery was 75% (256/338). It was higher in males [OR 1.88; 95%CI, 1.13-3.53, (*p*=0.01)]; whereas co-infection with HCV [OR, 0.12; 95% CI, 0.05–0.28 (*p* < 0.001)] and use of intravenous drugs (IVDU) [OR, 0.25; 95% CI, 0.07–0.85 (*p*=0.026)] were associated with a reduced likelihood of attaining a CD4+ cell gain > 100 cells/µl. Age, baseline CD4+ cell count, heterosexual (vs. homosexual) transmission, NNRTI (vs. PI)-based regimens and AIDS events were not associated with CD4+ recovery in this cohort.

**Conclusions:**

We found that a successful immune recovery is feasible in most of naïve patients at 48 weeks of effective antiretroviral therapy, even in those who start ART with low CD4+ cell counts or AIDS events. IVDU and HCV co-infection were deleterious for CD4+ reconstitution in our cohort. We did not found differences in CD4+ recovery with the use of PI- or NNRTI-based regimens.

# Sub article: P044 Prevalence of HIV-1 drug resistance associated mutations in patients experiencing first-line antiretroviral therapy failure in a cohort of HIV-positive patients from Santiago, Chile, 2012–2014

**DOI:** 10.7448/IAS.19.2.21073

**Published:** 2016-04-28

**Authors:** Fernando Bernal, Leonardo Chanqueo, Catalina Gutierrez, Patricia Vásquez

**Affiliations:** Hospital San Juan de Dios, Servicio de Medicina, Santiago, Chile

## Abstract

**Background:**

Antiretroviral therapy (ART) has dramatically decreased morbidity and mortality among HIV-1-positive patients through the durable suppression of viral replication to undetectable levels. However, the efficiency of these treatments can be compromised by the presence of drug resistance-associated mutations (DRMs), resulting in virological failure. Although international guidelines, such as those of the UK and the US, recommend a basal genotypic HIV resistance testing, in Chile this standard-of-care management test is recommended only in patients who experienced virological failure on first-line ART. The aim of this study was to estimate the prevalence of HIV-1 DRMs in patients experiencing first-line ART failure in a cohort of adults HIV-positive patients from Santiago, Chile.

**Materials and methods:**

Using restrospective data from our cohort from Hospital San Juan de Dios de Santiago, Chile, we included all the HIV genotypic testing request forms from adult HIV patients with ART failure – defined as a plasma viral load >1,000 RNA copies/ml – between 2012 and 2014 who initiated ART according to the Chilean HIV treatment guideline. The frequency of DRMs was analyzed from the HIV genotypic test reports, which were done by ViroSeq HIV-1 Genotyping System at the Chilean HIV reference laboratory (Laboratorio de Biología Molecular – Hospital Lucio Córdova, Santiago).

**Results:**

There were 20 requests for HIV genotypic resistance tests in 2012, 77 in 2013 and 79 in 2014. A total of 168 HIV genotypic resistance tests were requested, 134 (80%) were analyzed and 34 (20%) rejected because the viral load was lower than 1,000 RNA copies/ml. We determined that 99/134 (74%) patients had viruses harbouring DRMs. Among them, 69 (70%) patients had two-class resistance (nucleoside reverse transcriptase inhibitors (NRTI) and non-nucleoside reverse transcriptase inhibitors (NNRTI)). The most common NRTI and NNRTI DRMs detected were the M184V and K103N; M230L was detected only in three patients. Major PI mutations were found only in 3.82% of patients (4/99).

**Conclusions:**

Our findings emphasize the importance of using the genotypic test at the first treatment failures to guide the choice of an effective alternative regimen. In addition, our study gave insights on the distribution of DRMs in our population which are related to the initial ART regimen used. Two-class resistance (NRTI plus NNRTI) was very frequently developed, but major PI-mutations were infrequently detected and no etravirine or rilpivirine DRMs were found in patients who experienced virological failure on first-line ARV in our cohort.

# Sub article: P045 Ledipasvir/sofosbuvir (LDV/SOF) for 8W in genotype 1 treatment-naïve (TN) non-cirrhotic (NC) patients with viral load (VL) less than 6 million IU/ML; a comparative analysis of the ION-3 data to real work effectiveness

**DOI:** 10.7448/IAS.19.2.21074

**Published:** 2016-04-28

**Authors:** Jordan Feld, Peter Buggisch, Jorg Peterson, Stefan Mauss, Kris Kowdley, Michael Curry, Peter Ruane, Dani Ain, Naoky Tsai, Yoori Lee, Edward Eggleton, Macky Natha, Bruce Kretter, Diana Brainard, Nelson Cheinquer, Patrick Ingiliz

**Affiliations:** 1 Toronto Centre for Liver Disease, University of Toronto, Toronto, Canada; 2 Asklepios Klinik St. Georg, IFI Institut für Interdisziplinäre Medizin, Hamburg, Germany; 3 Center for HIV and Hepatogastroenterology, Dusseldorf, Germany; 4 Swedish Medical Center, Seattle, USA; 5 Beth Israel, Boston, USA; 6 Ruane Medical and Liver Health Institute, Los Angeles, USA; 7 Queens Medical Center, Honolulu, USA; 8 TRIO Health Analytics, Newton, USA; 9 Gilead Sciences, Los Angeles, USA; 10 Medical Affairs, Gilead Sciences, Foster City, USA; 11 Gilead, Public Health and Medical Affairs, Sao Paulo, Brazil; 12 Medizinisches Infektiologie Zentrum, Berlin, Germany

## Abstract

**Introduction and aims:**

The optimal duration of therapy to achieve sustained virological response (SVR) depends on multiple factors. Patients treated with ledipasvir/sofosbuvir (LDV/SOF) with 8–24 weeks achieved SVR12 from 94 to 100% in the ION Phase 3 studies. A decision to shorten therapy to 8 weeks is based on treatment history, cirrhosis status and baseline VL. In a post hoc analysis of the ION-3 (treatment-naïve (TN) non-cirrhotic (NC) patients) 8-week data, a viral load (VL)<6 million was shown to be the best predictor of SVR. Real work effectiveness (RWE) is often different from Phase 3 trials and there is a need to understand real-world 8-week regimens in a broader spectrum of patients.

**Methods:**

RWE 8-week LDV/SOF data are emerging from multiple single-centre and multicentre retrospective and prospective cohorts. In this analysis, the Phase-3 ION-3 data are compared with data from several diverse real-world populations and one post-marketing investigator sponsored HIV/HCV trial. Patient demographics, characteristics, SVR12 and discontinuation data have been collated and compared.

**Results:**

The ION-3 post hoc analysis reported 123 patients who were TN, NC (VL<6 million) and treated with 8 weeks of LDV/SOF. Mean age was 52 y, 22% were black, 72% had GT1a and the overall SVR12 was 97% (119/123). The overall SVR12 rate from six diverse real-world and post-marketing cohorts was also 97% (638/658). There was no significant impact of HCV genotypes or subtypes (GT1a, 1b versus GT4), prior treatment history, presence or absence of cirrhosis, high viral load (HCV VL>6 million) or HIV/HCV co-infection. All response rates are detailed in Table 1.

**Conclusions:**

LDV/SOF for 8 weeks yielded high SVR rates in ION-3. Analysis of RWE data from several diverse and heterogeneous cohorts from the United States and Europe shows SVR outcomes that were consistent with the Phase-3 ION-3 results and supports the use of 8 weeks LDV/SOF in treatment-naïve, non-cirrhotic GT1 patients with a baseline HCV VL <6 million and possibly in other populations including HIV/HCV co-infected patients. Discontinuation rates were low despite diverse patients and clinical settings. Data from the TARGET and TRIO cohorts also suggest that the 8-week regimen is under-utilized.

**Abstract P045–Table 1:** Demographic data and results from clinical trials and RWE studies using LDC/SOF for 8 weeks

Study	ION-3	TARGET	TRIO	Buggisch	GECCO	VA – Marshall	Ruane	Total (non ION3)
N/N (GT 1)	119/123	150/154	251/263	103/103	69/70	47/48	18/20	638/658
Age (mean)	52 (22-73)	58* (19-84)	57 (18-84)	50* (22-77)	52* (44-58)	61 (32-75)	52 (35-66)	n/a
HIV/HCV	0	1/1	3/3	3/3	7/7	0	18/20	32/34
VL >6 million	0	0	8/8	0	9/9	0	0	17/17
Cirrhotics	0	6/6	0	0	3/3	0	0	9/9
GT 4	0	0	0	2/2	0	0	0	2/2
Tx Exp	0	8/8	0	1/1	12/12	5/5	0	26/26
SVR12 (%)	97%	97%	95%	100%	99%	98%	90%	97%

* Median age used

# Sub article: P046 Characterization of a large hepatitis C patient cohort in a reference centre in Brazil: a descriptive cohort study

**DOI:** 10.7448/IAS.19.2.21075

**Published:** 2016-04-28

**Authors:** Rosario Quiroga, Andre Machado Luiz, Fatuma Ca Odongo, Maria Laura M De Matos, Ana Catarina Nastri, Aléia Faustina Campos, Ligia Capuani, Maria Cassia Mendes-Correa

**Affiliations:** Medical School – São Paulo University, Infectious Diseases, São Paulo, Brazil

## Abstract

**Introduction:**

Chronic Hepatitis C (CHC) affects approximately 2.7 million people in Brazil [1]. Until December 2015, the majority of patients have been treated with interferon-based therapy [2]. The use of direct acting agents (DAAs) in Brazil has been recently introduced and only for patients with severe liver disease [3].

**Objectives:**

The objectives of the present study were: 1) To describe the results of the clinical characteristics of CHC patients attended at a reference centre in Brazil; 2) To identify patients who are yet to be treated; 3) To differentiate easy-to-treat from difficult-to-treat patients.

**Materials and methods:**

We developed a retrospective and descriptive cohort study at Hospital das Clinicas, which is a public hospital and a reference centre to treat CHC in São Paulo, Brazil. The enrolled patients were selected from those registered at the Infectious Diseases Division. Medical records from all patients with CHC were reviewed in order to analyze selected variables: age, gender, HIV co-infection, HCV genotype, stage of liver fibrosis ( by using Metavir Score and/or evidence of portal hypertension) and information regarding previous hepatitis C treatment. In this study, difficult-to-treat patients were defined as follows: experienced genotype 3-infected or genotype 1-infected patients who failed first-generation protease inhibitors, cirrhotic or HIV co-infected patients.

**Results:**

Between April 2015 and December 2015, 1,757 patients with CHC were identified and included in our data bank. Of these patients, 219 (12.4%) had HIV co-infection. 893 (50.8%) were women, and mean age was 41.95±14.5 years. Genotype 1 predominated with 972 (55.3%) cases. Genotype 2, 3, 4 and 5 were 50 (2.85%), 513 (29.2%), 11 and 1 cases, respectively. Among all patients, 747 (42.5%) were F3 or F4, and 293 (16.6%) had cirrhosis. Among all patients, 1,048 (59.6%) had received previous hepatitis C treatment, with interferon-based therapy (*n*=901, 86%) or telaprevir/boceprevir-based therapy (*n*=147, 14%). Among 1,048 previously treated patients, only 480 (46%) obtained sustained virologic response (SVR) at a previous treatment and 250 were genotype 3 patients. Among all included patients, 1277 (73%) were yet to be treated and only 660 (37.5%) were considered difficult-to treat patients.

**Conclusions:**

In this Brazilian cohort from an urban tertiary medical centre, the majority of patients still wait for a proper HCV treatment. Less than half of those treated prior to DAAs had an SVR. However, the majority of patients in the cohort were considered easy-to-treat patients.

## References

[1] Pereira LM, Martelli CM, Moreira RC, Merchan-Hamman E, Stein AT, Cardoso MR (2013). Prevalence and risk factors of Hepatitis C virus infection in Brazil, 2005 through 2009: a cross-sectional study. BMC Infect Dis.

[2] Ferreira PR, Brandão-Mello CE, Estes C, Gonçales Júnior FL, Coelho HS, Razavi H (2015). Disease burden of chronic hepatitis C in Brazil. Braz J Infect Dis.

[3] Protocolo Clínico e Diretrizes Terapêuticas para Hepatite C e Coinfecç[otilde]es. http://www.aids.gov.br/publicacao/2015/protocolo-clinico-e-diretrizes-terapeuticas-para-hepatite-c-e-coinfeccoes.

# Sub article: P047 High prevalence of occult hepatitis B virus infection among blood donors with anti-hepatitis B core antibodies in the north-east region of Colombia

**DOI:** 10.7448/IAS.19.2.21076

**Published:** 2016-04-28

**Authors:** Henry Bautista-Amorocho, Yeny-Zulay Castellanos-Dominguez, Sandra-Lucía Saavedra-Cortés, Vianney Portilla-Rodríguez, Leonor Chacón-de-Mendieta, Ana-Elvira Farfán-García

**Affiliations:** 1 Universidad de Santander – UDES, Bacteriología y L.C. – CliniUDES Research Group, Bucaramanga, Colombia; 2 Laboratory of Public Health of the Province of Santander (LPHPS), Blood Bank Network, Bucaramanga, Colombia

## Abstract

**Introduction:**

Infection by hepatitis B virus (HBV) can occur through blood transfusions when routine screening of hepatitis B surface antigen (HBsAg) is negative. The aim of this study was to evaluate the seroprevalence of HBV infection and to identify occult hepatitis B infection (OBI) among donors with only antibodies against the hepatitis B core (anti-HBc).

**Materials and methods:**

A cross-sectional study was carried out in three blood banks in Bucaramanga city, Colombia, in 2013. Demographic and epidemiological information was collected from each donor, whereas HBV serological markers and DNA were investigated in serum by immunoassays and nested-PCR, respectively. A 1085 nucleotide fragment overlapping the s gene of hepatitis B virus was sequenced after cloning in a sequencing plasmid to identify the viral genotype.

**Results:**

In total, 61,188 blood donors were gathered during the study period, 45.2% were first-time volunteers and 57.9% were male. HBV serological markers were detected in 1,215 blood donors (2%), from which 0.2% were HBsAg(+) and 1.8% anti-HBc(+)/HBsAg(-). Differences were observed for types of blood donor, sex and age group with anti-HBc(+)/HBsAg(-) donors (*p*<0.001). OBI was detected in 7.3% of the donors with anti-HBc(+) antibodies from which 75% were anti-HBc(+)/anti-HBs (+) serological profile. Genotype F, subtype F3 was identified in all OBI donors. Only one mutation was found in a blood donor, specifically a serine-leucine non-conservative change in codon 143 (S143L).

**Conclusions:**

The 7.3% of OBI among donors with anti-HBc(+) antibodies in the north-east region of Colombia is the highest prevalence reported in blood banks of Latin America so far. As expected, F3 is the predominant viral subtype circulating in the country with low mutation rate inside the s gene. Molecular assays should be included in the screening for infectious diseases to reduce the risk of HBV transmission through blood donation.

# Sub article: P048 Viral hepatitis and syphilis prevalence in persons who performed premarital blood tests in Argentina

**DOI:** 10.7448/IAS.19.2.21077

**Published:** 2016-04-28

**Authors:** Patricia Angeleri, Valeria Levite, Gabriela Vidiella, Joaquin Solari, Ema Coronel, Dan Adaszko, Ariel Adaszko, Cecilia Moyano, Diosnel Bouchet, Hector Cuello, Viviana Molfese, Rosario Skarzauskas, Maria De Los Angeles Pando, Carlos Falistocco

**Affiliations:** 1 National AIDS and STIs Program, National Ministry of Health, Buenos Aires, Argentina; 2 Statistical Area, Ministry of Health, Santa Fe, Argentina; 3 HIV/AIDS & Viral Hepatitis Provincial Program, Cordoba, Argentina; 4 Central Hospital, Mendoza, Argentina; 5 HIV/AIDS & Viral Hepatitis Provincial Program, Buenos Aires, Argentina; 6 INBIRS, University of Buenos Aires, Buenos Aires, Argentina

## Abstract

**Introduction:**

Viral hepatitis and syphilis are important public health issues on which Argentina has no prevalence data in the general population. Prevalence studies are fundamental pillars to develop strategies for their control, contributing with knowledge about the needs for prevention, testing and treatment.

**Materials and methods:**

A cross-sectional study was designed with a cluster sampling strategy. People who attended health services in occasion of their premarital exams (which includes screening for syphilis) took part in the study by filling in a form and being tested for viral hepatitis. The recruitment was carried out in Buenos Aires, Greater Cordoba, Río Cuarto, Greater Mendoza, Greater Santa Fe and Rosario between September 2013 and October 2014.

**Results:**

A total of 3,835 persons participated in the study out of which 1,922 (50.6%) were women and 1,909 were men (49.4%).The estimated syphilis prevalence was 0.74% (95% CI: 0.47-1.01), finding statistically significant difference between the area where the marriages were going to take place and the educational level (lower educational level, higher prevalence). The estimated prevalence for hepatitis A was 63.9% (95% CI: 62.4-65.5) with differences regarding age (the older, the higher the prevalence), province of birth, educational level (lower educational level, higher prevalence), housing type (most prevalent in people with deficient housing) and health insurance (more prevalent in people with no health insurance). For hepatitis B, the estimated prevalence found for HBsAg was 0.26% (95% CI: 0.10–0.42) and 2% (95% CI 1.56–2.44) for anti-HBc. Finally, the estimated prevalence of hepatitis C found was 0.26% (95% CI: 0.10-0.43). The only statistically significant difference found was connected to province of birth and place of residence.

**Conclusions:**

The results are consistent with the findings in other studies and national publications. Despite differences in this population from the general population, the results found are a first step towards grasping the impact of viral hepatitis and syphilis on the young adult population of Argentina.

# Sub article: P049 A veil on the statistics of viral hepatitis: the impact of ICD-10 in mortality registration

**DOI:** 10.7448/IAS.19.2.21078

**Published:** 2016-04-28

**Authors:** Patricia Angeleri, Hernan Hernandez, Gabriela Vidiella, Ariel Adaszko, Carlos Guevel, Carlos Falistocco

**Affiliations:** 1 National AIDS and STIs Program, National Ministry of Health, Buenos Aires, Argentina; 2 Health Statistics and Information Program, National Ministry of Health, Buenos Aires, Argentina

## Abstract

**Introduction:**

Viral hepatitis (VH) B and C are a global public health problem. Their natural history implies chronicity, cirrhosis and hepatocellular carcinoma (HC). Several authors describe the difficulties in analyzing HV mortality from the underlying cause registered in the mortality reports.

**Materials and methods:**

A descriptive analysis for the period 1997–2013 of the ICD-10 codes registered on the databases of the Department of Health Statistics and Information (DEIS) of the Ministry of Health of Argentina was performed, the analyzed codes being B150, B159, B162, B169, B170, B171, B172, B178, B181, B182, B188, B189, B190, B199, B942. Bibliography was reviewed and an ICD-10 consultation was held to analyze how the underlying causes were assigned.

**Results:**

Between 1997 and 2013, 3,141 deaths were recorded in Argentina due to HV: 46.9% of the codes correspond to the event “Acute Hepatitis C (B171),” 20.6% “Acute Hepatitis B without delta agent without hepatic coma (B169)” and 11.7% “Chronic Viral Hepatitis C (B182).” These three events account for 79.2% of the total deaths (Figure 1). The revision of the index of ICD-10 showed that when the certifying physician does not indicate whether Hepatitis C is acute or chronic, the death must be coded into B171 “Acute Hepatitis type C” (8, 9). At the same time, as the ICD-10 indicates, “… Except in cases of disease by HIV, no infectious or parasitic disease may be accepted as causing a malignant tumour,” if a certificate states both HC and HV, the former must be selected as the underlying cause.

**Conclusions:**

The rules of the current ICD-10 explain the large number of deaths wrongly coded as “Acute Hepatitis type C” and the invisibility of VH as the underlying cause of HC. This implies a real obstacle to the adequate description of the impact of viral hepatitis on public health.

# Sub article: P050 Integrated analysis of emergent drug resistance through 96 and 144 weeks from clinical studies of HIV-1 treatment-naïve subjects receiving dolutegravir-based regimens

**Published:** 2016-04-28

**Authors:** Michael Aboud, Jim Demarest, Romina Quercia, Andrew Zolopa, Marty St Clair, Brian Wynne, Mark Underwood, Catherine Granier

**Affiliations:** 1 ViiV Healthcare, Brentford, UK; 2 ViiV Healthcare, Research Triangle Park, USA; 3 ViiV Healthcare, Upper Providence, PA, USA; 4 GlaxoSmithKline, Stockley Park, UK

## Abstract

**Introduction:**

The integrase inhibitor dolutegravir (DTG) plus 2 NRTIs has been evaluated in three Phase 3 studies in treatment-naïve subjects. Dolutegravir-based regimens (DBRs) achieved non-inferiority in SPRING-2 versus RAL-based regimens, while superiority was achieved in SINGLE and FLAMINGO versus Atripla (ATR) and boosted darunavir (DRV/r)-based regimens, respectively.

**Methodology:**

Genotypic and phenotypic resistance was analyzed on paired plasma from baseline and protocol-defined virologic failure (PDVF), regardless of plasma viral load (pVL) at PDVF. The frequency of reportable results with respect to pVL at PDVF was assessed.

**Results:**

In total, 1,067 patients received a DBR. Table 1 summarizes pVL at PDVF and by reportable results. The range of pVL at PDVF was similar between arms and generally decreased at the confirmatory visit. No DTG or NRTI resistance has been selected in the DBRs. Only a few subjects in the ATR (*n*=7, SINGLE) and RAL (*n*=4, SPRING-2) arms had detectable resistance at PDVF. A minority of subjects across studies had pVL >500 c/mL at PDVF and using this cut-off for resistance analysis would have detected resistance in 6/7 ATR and 1/4 RAL PDVF.

**Conclusions:**

DBRs demonstrated durable virologic suppression across three trials in treatment-naïve patients through 96 and 144 weeks of treatment. Plasma viral load at time of PDVF was comparable between arms, and reportable resistance results were obtained across a range of pVL, including pVL <500 c/mL at PDVF. No resistance to DTG or NRTIs has been detected to date in clinical trials of treatment-naïve patients receiving DBRs. Use in clinical practice will further inform the virologic characteristics of failure on a DBR.

**Abstract P050–Table 1:** Summary of pVL at PDVF and by reportable results according to class.

			PDVF		Reportable Results†		Resistance Detected#	
					
Study	*N*	Arm	*n*	Log c/mL pVL Median (Range)	*n* (IN : PR/RT)	Log c/mL pVL Median (Range)	*n* (IN : PR/RT)	Log c/mL pVL Median (Range)
**SINGLE Wk 144**	414	DTG	39	2.14 (1.72–5.61)	19:26	IN: 3.13 (1.72–5.61)/ PR/RT: 2.59 (1.82–5.61)	0 : 0	na
	419	ATR	33	2.07 (1.70–4.91)	11:16	IN:3.82 (1.75–4.91)/ PR/RT: 3.12 (1.70–4.91)	0 : 7	3.84 (2.12–4.61)
**SPRING-2 Wk 96**	411	DTG	22	1.94 (1.71–4.09)	10:14	IN: 2.20 (1.76–4.09)/ PR/RT: 2.36 (1.73–4.09)	0 : 0	na
	411	RAL	29	1.93 (1.70–4.95)	20:20	IN: 1.93 (1.70–4.95)/ PR/RT: 2.10 (1.72–4.95)	1 : 4	4.95 1.96 (1.72–4.95)
**FLAMINGO Wk 96**	242	DTG	2	3.17 (2.82, 3.36)	2:2	IN: 3.17 (2.82–3.36)/ PR/RT: 3.17 (2.82–3.36)	0 : 0	na
	242	DRV/r	4	3.62 (2.34–4.79)	4:4	IN: 3.62 (2.34–4.79)/ PR/RT: 3.62 (2.34–4.79)	0 : 0	na

† IN: Integrase, PR/RT: protease/reverse transcriptase;

# na=not applicable.

# Sub article: AUTHOR INDEX

**Published:** 2016-04-28

## A

**Table:** 

Aboud, M	P039*, P050*
Adaszko, A	P022*, P032, P048, P049
Adaszko, D	P048
Ain, D	P045
Alave, J	P007
Albora, CD	P035
Almond, S	P039
Andrade Villanueva, J	P004*, P007
Angeleri, P	P048*, P049*
Arauz, AB	O224
Arbelaez, F	P006
Arribas, J	P003
Arteta, Z	P035
Attwood, S	P030
Aylott, A	P039
Ayma, A	P032

## B

**Table:** 

Badial Hernandez, F	P018, P033
Bank, L	O225
Bastos Cabral, G	P015
Bautista-Amorocho, H	P047*
Becerril-Vargas, E	P031, P041
Bekker, L-G	P020
Belaunzaran, F	P038
Belaunzarán Zamudio, P	O222*, P005*, P025
Belloso, W	P007
Beltran, C	P037*
Bennett, S	P020
Benzaken, A	P040
Bernal, F	P042, P044
Bird, M	P021
Bonhomme, J	O313, O314
Bouchet, D	P048
Brainard, D	P008, P009, P045
Bravo-García, E	P036*
Brezzo, C	P022, P032
Brinson, C	P003, P004
Brunetta, J	P003
Buggisch, P	P045
Bulló, M	P016

## C

**Table:** 

Cabrera López, T de J	P033*
Cabrera, A	P035
Cabrera, S	P035
Callebaut, C	P010
Camilo Campos, N	P015
Campos, AF	P046
Capuani, L	P046
Caro-Vega, Y	P005, P034
Carrasco-Hernández, R	P005
Caserta, B	P035
Casillas Rodriguez, J	P018, P028*
Cassetti, I	O224*
Castellanos-Dominguez, Y-Z	P047
Castillo, A	O224
Celi, AP	P038
Cendejas, L	P021
Cerqueira, N	O223
Chacón-de-Mendieta, L	P047
Chahin, C	P037
Chanqueo, L	P042*, P044*
Cheinquer, N	P008, P009, P045
Chéret, A	P003
Chokephaibu, K	P020
Cid Vasque, H	P018
Clarke, A	P003, P004, P010
Clotet, B	P010
Clumeck, N	P003
Conde-Glez, CJ	P019
Cooper, C	P008, P009
Coronel, E	P048
Crabtree-Ramirez, B	P034
Cruz, JB	P001*, P002, P025, P029, P033
Cuello, H	P048
Curry, M	P045

## D

**Table:** 

Daar, E	P004
Das, M	P003, P010
Davis, A	P026
de Kort, J	O225
de Macedo Brígido, LF	P015
De Matos, MLM	P046
De Oliveira, T	O225
De Paz, M	P007
de Souza Cavalcanti, J	P015
Demarest, J	P050
Deschamps, MM	O313*, O314
Devieux, J	P030
Díaz, S	P017*, P033
Diego-Diaz, S	P005
Dieterich, D	P009
Domingo, R	P024
Domingues, CSB	P011*, P012*, P023*, P027*
Dorvil, N	P030*
Durán, A	P022, P032*
Duran, JA	P024

## E

**Table:** 

Eggleton, E	P045

## F

**Table:** 

Falistocco, C	P032, P048, P049
Farfán-García, A-E	P047
Feld, J	KL11*, P045*
Fernández, M	P022
Ferreya, VD	P001, P002, P029
Fonseca Pacheco, AG	O223
Fordyce, M	P003, P004, P010
Fourie, J	P020
Frantchez, V	P035*
Freitas, M	P040
Friborg, S	P004

## G

**Table:** 

Galarraga, O	P019
Gallo, C	P037
Ganley, KY	P019*
Garcia-Vega, M	P021
Gaur, A	P020
Georges Kallás, E	O223
German, P	P008, P009
Ghidinelli, M	O231*
Giadalah, C	P042
Gianna, MC	P012, P023, P027
Gladstone, D	P013
Gonzalez Rodríguez, A	P018, P025, P028, P029, P033
Gonzalez, A	P001, P002, P017
Granier, C	P050
Gras Allain, N	P018
Greco, M	P038*
Guevel, C	P022, P049
Guiteau, C	P030
Gulick, R	KL33*
Guo, S	P004
Gutierrez, C	P042, P044

## H

**Table:** 

Hakim, A	P016
Hennessey, K	P030
Hernandez, H	P049
Hirata, AH	P001, P002*, P029
Hofstra, LM	O225
Hunt, P	O221*

## I

**Table:** 

Ingiliz, P	P045
Iracheta, P	P017
Irizarry-Gonzalez, L	P006
Ivalo, S	P013, P016*

## J

**Table:** 

Jannat-Khah, D	O313, O314
Jimenez Munguia, LM	P018
Jovet-Toledo, GG	P006
Juarez, L	P017
Julma, P	O313, O314, P030

## K

**Table:** 

Kaynar, V	P032
Kelly, K	O225
Kizito, H	P020
Kleinstein, SE	P008, P009
Koenig, E	P010*, P020*
Koenig, S	P030
Kolling, A	P040
Kowdley, K	P008, P045
Kretter, B	P045
Kundro, M	O226*, P043
Kuritzkes, DR	KL31*
Kwo, P	P008

## L

**Table:** 

Lamastro, SM	P023
Langarica Naves, E	P033
Lantero, MI	O232*
Laplume, H	P007
Laurido, M	P003*, P004
Lee, Y	P045
Lenis, W	O224
León Juárez, EA	P031, P041
Levite, V	P022, P048
Lim, J	P039
Lima Hottz, P	O223
Lopez, O	P037
Losso, M	O226, P013*, P007, P016, P043
Luiz, AM	P046
Lurie, MN	P019

## M

**Table:** 

Macias, I	P017
Madrigal-Iberri, C	P034
Magis Rodríguez, C	P014, P031, P041
Marcelin, A	P030
Mellilo Sauer, M	O223
Marincovich, B	P037
Marshall, BDL	P019
Martin, H	P004
Martinez, E	P038
Mauss, S	P045
Mayer, K	O121*, P019
McNairy, M	O313, O314, P030
Medina, Y	P017
Mejia, F	P038
Mendes-Correa, MC	P046
Mesquita, F	P040
Molfese, V	P048
Molina-Martinez, D	P028
Milani da Silva, K	O223
Monsanto, H	P006
Monterio Matsuda, E	P015
Morales, M	O224
Mosqueda, L	P007
Moura, M	P040*
Moyano, C	P048
Mudrikova, T	O225

## N

**Table:** 

Nachman, S	P013
Naggie, S	P008, P009
Naik, S	P008, P009
Nardi, S	P022, P032
Nastri, AC	P046
Natha, M	P008, P009, P045
Niño, R	P017

## O

**Table:** 

Odongo, FCa	P046
Oka, S	P003
Operario, D	P019
Orengo, JC	P006
Orkin, C	P010
Orlando, M	P032
Ortega, D	P021
Ortiz Valenzuela, MA	P021
Ortiz-Pérez, H	P036

## P

**Table:** 

Pando, MDLA	P048
Pape, JW	O313, O314, P030
Parenti, P	O224
Parks, D	P004
Pascom, AR	P040
Paulino, R	P024*
Peña, P	P024
Perez, E	P022, P032
Perodin, C	O313, O314
Peterson, J	P045
Pineirua Menendez, A	P018
Piñeirúa, A	O312*, P033
Placco, AL	P023
Podzamczer, D	P010
Polon, MC	P027
Ponce de León-Rosales, S	P005
Pongelupi, SM	P023
Portilla-Rodríguez, V	P047
Prasitsuebsa, W	P020

## Q

**Table:** 

Quercia, R	P039, P050
Quirk, E	P020
Quiroga, R	P046*

## R

**Table:** 

Rael, C	P026*
Rafaela Silva, G	P015
Rakhmanina, N	P020
Ramos Alamillo, U	P033
Ramos Jr, AN	P023*
Reyes-Fentanes, MJ	P034*
Rios-Ellis, B	P021*
Rivera Reyes, M del P	P014
Rivera, V	P030
Rocabert, C	P001, P002, P029
Rodrigues, C	P015
Rodriguez Loria, G	P007*
Rodriguez, E	P017
Rodriguez, V	P001, P002, P029
Rodriguez-Diaz, CE	P006
Rodriguez-Lauzurique, M	P024
Román, E	P025*
Ron-Suarez, Y	P006
Rouzier, V	O313, O314*
Ruane, P	P045
Ruchansky, D	P035
Ruiz, V	P017

## S

**Table:** 

Saag, M	KL32*, P009*
Saavedra-Cortés, S-L	P047
Sánchez, A	P025
Sanchez Rivas, E	O225*
Santana, J	P006*, P008*, P009
Santiago-Rodriguez, EI	P006
Santos, MTF	P023
Sax, P	P003
Scalise, C	P016
Schechter, M	O223*
Sengupta, D	P010
Serri, M	P042
Severe, P	P030
Shao, Y	P020
Sierra-Madero, J	P005, P034
Silva López Lopes, GI	P015*
Silva, A	P010, P020
Silva, MA	P011, P012, P023
Skarzauskas, R	P048
Sobhie Diaz, R	O213*
Solari, J	P048
Sosa, N	O212*
Sosa-Rubi, SG	P019
Souza, TRC	P023
Sparinger, W de A	P023
St Clair, M	P050
Stamm, L	P008, P009
Sued, O	O211*
Sulkowski, M	P008, P009
Surveillance of Maternal and Infant Mortality	P012

## T

**Table:** 

Tancredi, MV	P027
Tapia, L	P024
Tashima, K	P003
Tayra, A	P011, P012, P027
Teran, R	O224
Terwel, S	O226, P016
Toibaro, J	O226, P043
Trottier, B	P004
Tsai, N	P045

## U

**Table:** 

Underwood, M	P050
Uribe Zúñiga, P	P014

## V

**Table:** 

Valenzuela-Lara, M	P031*, P041*
Vargas González, H	P018*
Vargas, C	P038
Vásquez, P	P042, P044
Vega, EH	P001, P002, P025, P029*
Vidiella, G	P048, P049
Vila, M	P022, P032
Villafuerte Garcia, A	P014*
Viloria, G	O226, P007, P043*
Vincent, T	P039
Volkow, ND	KL12*

## W

**Table:** 

Walmsley, S	O311*
Wensing, A	O225
Wohl, D	P003
Wolf, J	P008, P009
Wynne, B	P050

## Z

**Table:** 

Zhong, L	P003
Zitko, P	P037
Zolopa, A	P050
Zullo, AR	P019
